# DNA Prime/Adenovirus Boost Malaria Vaccine Encoding *P. falciparum* CSP and AMA1 Induces Sterile Protection Associated with Cell-Mediated Immunity

**DOI:** 10.1371/journal.pone.0055571

**Published:** 2013-02-14

**Authors:** Ilin Chuang, Martha Sedegah, Susan Cicatelli, Michele Spring, Mark Polhemus, Cindy Tamminga, Noelle Patterson, Melanie Guerrero, Jason W. Bennett, Shannon McGrath, Harini Ganeshan, Maria Belmonte, Fouzia Farooq, Esteban Abot, Jo Glenna Banania, Jun Huang, Rhonda Newcomer, Lisa Rein, Dianne Litilit, Nancy O. Richie, Chloe Wood, Jittawadee Murphy, Robert Sauerwein, Cornelus C. Hermsen, Andrea J. McCoy, Edwin Kamau, James Cummings, Jack Komisar, Awalludin Sutamihardja, Meng Shi, Judith E. Epstein, Santina Maiolatesi, Donna Tosh, Keith Limbach, Evelina Angov, Elke Bergmann-Leitner, Joseph T. Bruder, Denise L. Doolan, C. Richter King, Daniel Carucci, Sheetij Dutta, Lorraine Soisson, Carter Diggs, Michael R. Hollingdale, Christian F. Ockenhouse, Thomas L. Richie

**Affiliations:** 1 US Military Malaria Vaccine Program, Naval Medical Research Center, Silver Spring, Maryland, United States of America; 2 US Military Malaria Vaccine Program, Walter Reed Army Institute of Research, Silver Spring, Maryland, United States of America; 3 Radboud University Nijmegen Medical Center, Nijmegen, The Netherlands; 4 GenVec, Inc., Gaithersburg, Maryland, United States of America; 5 USAID, Washington, D. C., United States of America; Aeras, United States of America

## Abstract

**Background:**

Gene-based vaccination using prime/boost regimens protects animals and humans against malaria, inducing cell-mediated responses that in animal models target liver stage malaria parasites. We tested a DNA prime/adenovirus boost malaria vaccine in a Phase 1 clinical trial with controlled human malaria infection.

**Methodology/Principal Findings:**

The vaccine regimen was three monthly doses of two DNA plasmids (DNA) followed four months later by a single boost with two non-replicating human serotype 5 adenovirus vectors (Ad). The constructs encoded genes expressing *P. falciparum* circumsporozoite protein (CSP) and apical membrane antigen-1 (AMA1). The regimen was safe and well-tolerated, with mostly mild adverse events that occurred at the site of injection. Only one AE (diarrhea), possibly related to immunization, was severe (Grade 3), preventing daily activities. Four weeks after the Ad boost, 15 study subjects were challenged with *P. falciparum* sporozoites by mosquito bite, and four (27%) were sterilely protected. Antibody responses by ELISA rose after Ad boost but were low (CSP geometric mean titer 210, range 44–817; AMA1 geometric mean micrograms/milliliter 11.9, range 1.5–102) and were not associated with protection. *Ex vivo* IFN-γ ELISpot responses after Ad boost were modest (CSP geometric mean spot forming cells/million peripheral blood mononuclear cells 86, range 13–408; AMA1 348, range 88–1270) and were highest in three protected subjects. ELISpot responses to AMA1 were significantly associated with protection (p = 0.019). Flow cytometry identified predominant IFN-γ mono-secreting CD8+ T cell responses in three protected subjects. No subjects with high pre-existing anti-Ad5 neutralizing antibodies were protected but the association was not statistically significant.

**Significance:**

The DNA/Ad regimen provided the highest sterile immunity achieved against malaria following immunization with a gene-based subunit vaccine (27%). Protection was associated with cell-mediated immunity to AMA1, with CSP probably contributing. Substituting a low seroprevalence vector for Ad5 and supplementing CSP/AMA1 with additional antigens may improve protection.

**Trial Registration:**

ClinicalTrials.govNCT00870987.

## Introduction

According to the World Health Organization, malaria caused an estimated 216 million clinical cases and 655,000 deaths in 2011 [1], underscoring the urgent need for an effective vaccine [2]. Developing a vaccine should be feasible, based on evidence of durable sterile immunity induced in humans by the bites of *Plasmodium falciparum*-infected mosquitoes. In these models, sporozoites are injected into the human host but development is aborted in the liver by prior irradiation of the infected mosquitoes [3] or in the blood by co-administering a drug selectively active against blood stage parasites such as chloroquine [4], [5]. While the immunological mechanisms underlying the high grade protection induced by these whole parasite vaccines remain unclear, animal studies demonstrate dependence on cell-mediated immunity (CMI), in particular cytotoxic CD8+ T cells [6], [7] which release cytotoxins to lyse the malaria-infected hepatocyte or interferon gamma (IFN-γ) to trigger nitric oxide production [8], [9]. Trials are in progress to evaluate the feasibility of immunizing humans by needle injection of purified, cryopreserved, irradiated sporozoites [10]. At the same time, vaccine developers are striving to provide equal protection via a cell-mediated mechanism using subunit vaccines.

The most advanced subunit candidate vaccine is RTS,S, containing the immunodominant sporozoite surface antigen circumsporozoite protein (CSP) fused to hepatitis B surface protein. RTS,S provides 50% protection against controlled human malaria infection, mediated primarily by the induction of potent antibody responses targeting sporozoites [11], [12]. RTS,S does not appear to induce CD8+ T cell responses, limiting its ability to control the intracellular hepatic stages of *Plasmodium*. Gene-based technologies are an attractive alternative to induce CD8+ T cell responses targeting these stages [13]. Many gene-based vaccines are licensed for use in veterinary medicine, but only the live-attenuated yellow fever-Japanese encephalitis chimeric vaccine (IMOJEV) has been licensed for human use (Australia) [14]. Microbial genes are inserted into a DNA plasmid with expression controlled by a promoter sequence activated within the host cell. Alternatively, the genes are inserted into a viral vector, which efficiently transports the DNA into the host cell. With either plasmids or viral vectors, parasite proteins are expressed within the cytoplasm rather than supplied exogenously as in the case of RTS,S. This leads to activation of MHC Class I (endogenous) antigen presentation, generating CMI including cytotoxic CD8+ T cells.

This trial was designed to test the gene-based approach to subunit malaria vaccines, supported by encouraging results of DNA- and virally-vectored constructs in mice, non-human primates, and humans [15], [16], [17], [18], [19]. Based on improved protection in animal models with heterologous prime-boost regimens [20],[21], subjects were primed three times with a mixture of two DNA plasmids (DNA) and boosted once with a mixture of two non-replicating recombinant human serotype 5 adenovirus vectors (Ad). CSP was chosen as one antigen because of its expression by sporozoites and early liver stage parasites coupled with its protective role in animal models and in the RTS,S vaccine, and apical membrane antigen-1 (AMA1) was chosen as the second antigen because of expression by sporozoites and liver stage parasites [22]. In addition, AMA1 carries the potential to induce a second line of defense, as it is expressed by blood stage parasites, can protect against blood stage malaria in animal studies [23] and is associated with clinical immunity to malaria in humans in endemic areas [24].

Two antigens were used on the premise that immune responses effectively targeting multiple parasite antigens and stages could induce complementary and potentially synergistic immune responses. CSP is carried into hepatocytes following sporozoite invasion [25] and this leads to the expression of peptides derived from CSP on the surface of the infected hepatocytes in the context of MHC Class I, allowing recognition by CSP-specific CD8+ T cells [26]. AMA1, like CSP, is involved in hepatocyte invasion [22], and peptides derived from this antigen may likewise be expressed on the surface of infected hepatocytes. Having peptides derived from both antigens expressed by infected hepatocytes may facilitate their targeting by effector cells. The trial demonstrated that DNA priming/Ad boosting (DNA/Ad) induced sterile immunity in 27% of volunteers, better protection than previously reported for other prime-boost malaria vaccines [17],[27] and with evidence of a contribution from both antigens supporting this contention. As predicted, protection was significantly associated with CMI. There was no apparent contribution by antibodies.

## Methods

The protocol for this trial and supporting CONSORT checklist are available as supporting information; see Protocol S1 and Checklist S1.

### Objectives

The primary objective of this study was to assess the safety of a heterologous prime-boost vaccine regimen (DNA/Ad) in healthy malaria-naïve adults. Secondary objectives were to assess protective efficacy against sporozoite challenge by *P. falciparum*-infected mosquitoes and to look for an association between protection and humoral responses measured by enzyme linked immunosorbent assay (ELISA) and cellular responses measured by enzyme linked immunospot assay (ELISpot) and flow cytometry/intracellular cytokine staining (ICS). Exploratory objectives were to measure humoral responses to sporozoites by immunofluorescence assay (IFA) and, due to the inclusion of AMA1 in the vaccine, to blood stages by growth inhibition assay (GIA), and to measure the effect of pre-existing neutralizing antibodies to adenovirus serotype 5 (NAb) on immunogenicity and protection.

### Ethics

The study protocol for the clinical trial was approved by the Institutional Review Boards at the Walter Reed Army Institute of Research (WRAIR) and the Naval Medical Research Center (NMRC). The study was conducted at the WRAIR Clinical Trials Center in accordance with: the principles described in the Nuremberg Code and the Belmont Report; all federal regulations regarding the protection of human participants as described in 32 CFR 219 (The Common Rule) and instructions from the Department of Defense, the Department of the Army, the Department of the Navy and the Bureau of Medicine and Surgery of the United States Navy; and the internal policies for human subject protections and the standards for the responsible conduct of research of the US Army Medical Research and Materiel Command (USAMRMC) and the Naval Medical Research Center (NMRC). WRAIR holds a Federal Wide Assurance from the Office of Human Research Protections (OHRP) under the Department of Health and Human Services as does NMRC. NMRC also holds a Department of Defense/Department of the Navy Federal Wide Assurance for human subject protections. All key personnel were certified as having completed mandatory human research ethics education curricula and training under the direction of the WRAIR IRB or the NMRC Office of Research Administration (ORA) and Human Subjects Protections Program (HSPP). All potential study subjects provided written, informed consent before screening and enrollment and had to pass an assessment of understanding.

### Study Design

**Figure 1:**
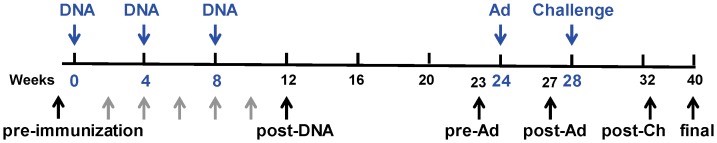
Trial design. Subjects were immunized week 0, 4, 8 and 24 and challenged week 28 (blue arrows). Samples for measuring cell-mediated immunity (ELISpot assay and flow cytometry) were collected at six time points (black arrows), and for measuring antibody levels (ELISA, IFA and growth inhibition assay) at similar time points plus after the DNA immunizations (gray arrows). See text for details.

This study was an open-label, Phase 1 trial, with controlled human malaria infection (CHMI) to assess protection. Volunteers received DNA at weeks 0, 4, and 8 and Ad at week 24 and were monitored for adverse signs and symptoms, laboratory abnormalities, and humoral and cellular immune responses (Figure 1). Fifteen immunized volunteers, plus six unimmunized infectivity controls, were challenged with *P. falciparum* (strain 3D7) via five infectious mosquito bites at week 28. Blood was collected and Giemsa-stained malaria smears read by certified microscopists on days 6 through 21 post-challenge, then every other day through day 28 in volunteers remaining smear negative. Positive volunteers were treated with 1500 mg chloroquine base over three days and followed daily until three consecutive negative smears had been documented. Quantitative polymerase chain reaction (qPCR) was performed after the trial on blood samples collected twice daily (morning and evening) from days 6 to 16 post challenge [28].

### Study Subjects and Eligibility

Enrollment was limited to healthy adults age 18–50 years who passed screening by medical history, physical examination, electrocardiogram and laboratory testing (criteria in the supplement). Cardiac risk screening was conducted to identify and exclude individuals at moderate or high risk of developing symptomatic coronary artery disease during the next 5 years, based on gender, blood pressure, body mass index, smoking history and presence or absence of diabetes [29]. This was done to avoid the physiologic stress of malaria infection in individuals with occult coronary artery disease. Pre-existing anti-adenovirus serotype 5 neutralizing antibodies [30] (NAb) (90% neutralization titer) were measured during screening and also prior to Ad immunization to assess potential effects on vaccine potency.

### Vaccines

The combined prime boost regimen, the NMRC-M3V-D/Ad-PfCA Vaccine, contains a priming component, the NMRC-M3V-D-PfCA Vaccine (Naval Medical Research Center, multi-antigen, multi-stage malaria vaccine, DNA-vectored, *P. falciparum* CSP and AMA1 antigens), and a boosting component, the NMRC-M3V-Ad-PfCA Vaccine (Naval Medical Research Center, multi-antigen, multi-stage malaria vaccine, adenovirus [serotype 5]-vectored, *P. falciparum* CSP and AMA1 antigens). This study was conducted under IND Number BB-IND 13977, allowed on April 07, 2009.

**Figure 2:**
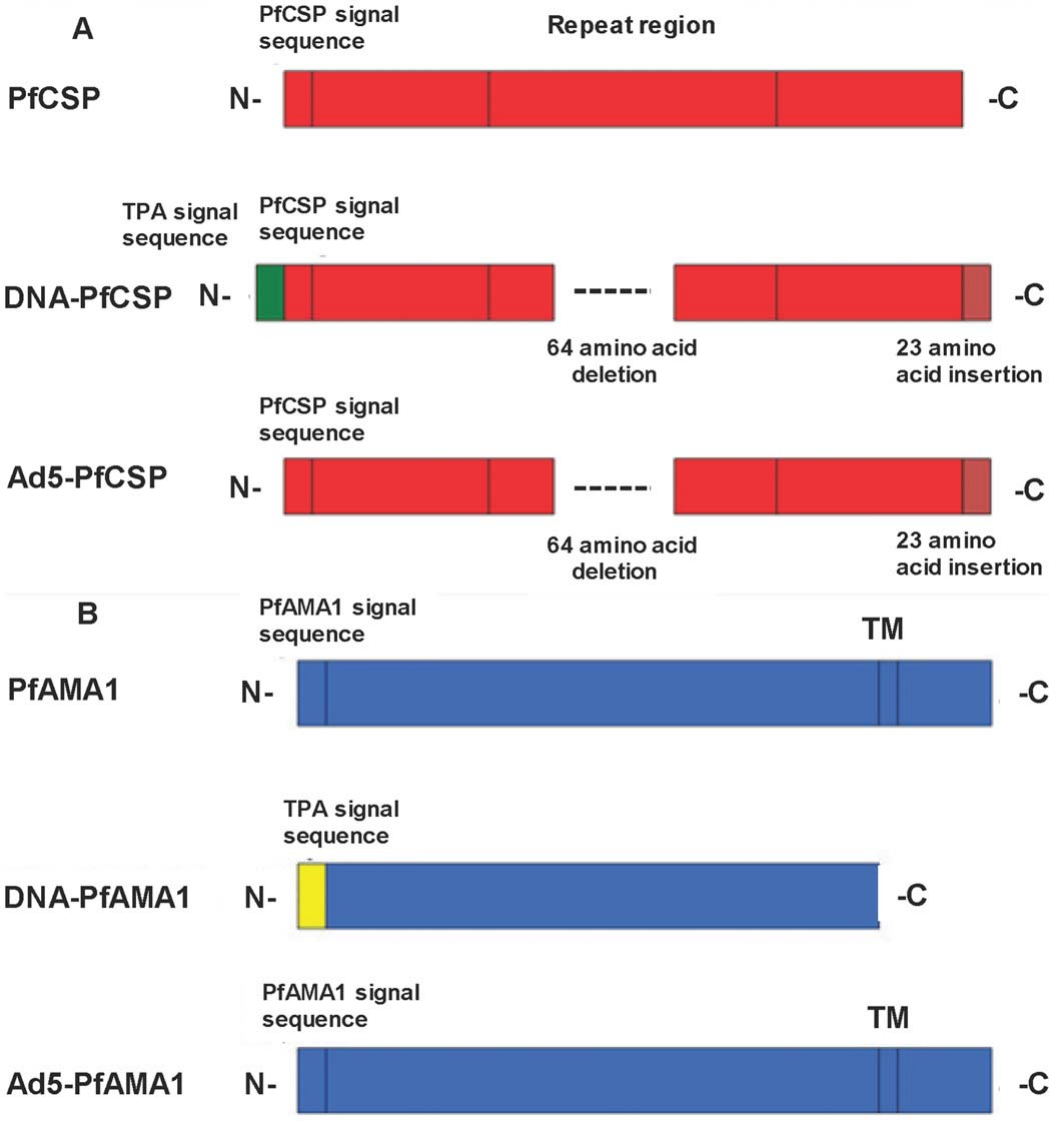
Schematic of DNA and Adenovirus CSP and AMA1 vaccines. Each panel presents the native protein (top of each panel) and the protein expressed by the DNA or Ad construct (middle and bottom of each panel) for the CSP (Panel **A**) and AMA1 (Panel **B**) vaccine antigens. N = amino terminus; C = carboxy terminus; TPA = human tissue plasminogen activator signal sequence; TM = transmembrane domain. See text for explanation. Identical colors indicate identical sequences. Not represented is a single amino acid substitution (G → R) in the AMA DNA construct at position 143.

NMRC-M3V-D-PfCA (DNA) (Figure 2, Panel A): CSP and AMA1 genes from 3D7 strain were codon-optimized for expression in mammalian cells and inserted into plasmid VR1020 (Vical, Inc., San Diego, CA). The CSP gene was modified by deletion of 16 of the central repeat sequences (64 amino acids), by adding a human tissue plasminogen activator (TPA) signal sequence to the native signal sequence at the N terminus (to increase expression in mammalian cells) and a 23 amino acid segment from the transcriptional terminator of bovine growth hormone at the C terminus (also appears to increase expression), while the AMA1 gene expressed the AMA1 ectodomain and was modified by replacing the native signal sequence with a TPA signal sequence. Expression was controlled by the promoter/enhancer of the human cytomegalovirus immediate early (CMV IE) gene. Each plasmid contained two open reading frame sequences: one encoded the kanamycin resistance protein which is expressed in bacterial cells and the other encoded a human tissue plasminogen activator protein (hTPA) leader/malaria fusion protein which is expressed in mammalian cells. The plasmids were produced under cGMP from bacterial cells in kanamycin selective media. The two DNA plasmid constructs were manufactured, mixed and vialed by Vical, Inc. (San Diego, CA). They were administered intramuscularly at 2 mg per dose (1 mg each construct) by needle-free jet injection (Biojector 2000®, Bioject, Inc., Tualatin, OR) as two concurrent 1 mL injections, one to each deltoid muscle (concentration of mixed plasmid DNA 1 mg/mL). The clinical testing of a similar DNA-CSP vaccine (non-codon-optimized) has been previously reported [18],[31],[32],[33].

NMRC-M3V-Ad-PfCA (Ad) (Figure 2, Panel B): As with the DNA, the CSP and AMA1 3D7 genes were codon-optimized for expression in mammalian cells and were inserted into the E1 region of the adenovirus vector under the transcriptional control of a modified human cytomegalovirus promoter (CMV-IE). The CSP gene was identical to the DNA construct but lacked the TPA signal sequence, while the AMA1 gene was full length, including the transmembrane and cytoplasmic domains in addition to the ectodomain, and also contained the native signal sequence rather than the TPA signal sequence. The serotype 5 Ad vector was derived from GV11D (GenVec, Inc., Gaithersburg, MD) and was missing the E1 and E4 regions required for replication as well as part of the E3 region. The two constructs were vialed separately and were mixed prior to intramuscular administration by needle as a single 1 mL deltoid injection at 2×10^10^ particle units (pu) per dose (1×10^10^ pu each construct). The clinical testing of the NMRC-M3V-Ad-PfCA Vaccine has been previously reported [15],[16].

### Safety and Tolerability

Adverse events (AEs) were recorded after each immunization to evaluate safety, tolerability and reactogenicity. Solicited AEs were recorded on days 0, 1, 2 and 7, unsolicited AEs on days 0, 1, 2, 7, 14 and 28 and laboratory tests (complete blood count, aspartate aminotransferase (AST), alanine aminotransferase (ALT), creatinine and total bilirubin) on days 0, 2, 7 and 28 following each immunization. Monitoring for serious AEs (SAEs) was performed until the week 40 termination of the study.

### Immunological Endpoints

Samples for measuring cell-mediated immunity (ELISpot assay and flow cytometry) were collected pre-immunization, 28 days post the third DNA (post-DNA), 105 days post the third DNA/seven days prior to Ad (pre-Ad), 22/23 days post Ad/five or six days pre-challenge (post-Ad), 28 days post challenge (post-Ch) and 84 days post challenge (post-Ch final). Antibody levels (ELISA, IFA and growth inhibition assay) were measured at similar time points and also at 14 and 28 days after each DNA immunization (Figure 1).

#### Antibody responses

Anti-CSP and AMA1 antibodies were measured by enzyme-linked immunosorbent assay (ELISA) against the CSP repeat region using a hexameric synthetic peptide (NANP)_6_ and AMA1 using recombinant ectodomain protein [16], and immunofluorescent antibody assay (IFA) using air-dried sporozoites [34]. Neutralizing antibodies to Ad5 (Nab) were measured as previously described [30].

#### Interferon-gamma Enzyme Linked Immunospot Assays (IFN-γ ELISpot Assays)

T cell responses were measured by IFN-γ ELISpot assay [15] using fresh peripheral blood mononuclear cells (PBMC). Peptides used for ELISpot assays were synthesized by Mimotopes, VIC, Australia (80% purity). The full length *P. falciparum* 3D7 CSP sequence (GenBank no. X15363) and *P. falciparum* 3D7 AMA1 sequence (GenBank no. XM1347979) were covered by a series of 15 amino acid (aa) peptide sequences overlapping by 11 aa. CSP 15mers were combined into 9 pools (Cp1-Cp9) each containing three to 12 peptides, and AMA1 15mers were combined into 12 pools (Ap1-Ap12) each containing 10–13 peptides. PBMC were stimulated for 36 hours with the 9 individual CSP or the 12 individual AMA1 peptide pools using previously described methods [15]. A positive response was defined as a significant difference (p = <0.05) between the average of the number of spot forming cells (sfc) in test wells and the average of negative control wells (Student’s two tailed *t*-test), plus at least a doubling of sfc in test wells relative to negative control wells, plus a difference of at least ten sfc between test and negative control wells.

#### Characterization of IFN-γ -producing cells by cell depletion or enrichment studies

ELISpot assays were carried out with PBMC after depletion of T cell subsets using anti-human CD4+- or anti-CD8+-coated Dynabeads M-450 (Dynal, Great Neck, NY) following the manufacturer’s instructions and as previously reported [15]. Mock depletion was done by using Dynabeads coated with sheep anti-mouse IgG. Flow cytometry confirmed that T-cell subset depletions were >99% in all experiments. The data are presented as the percent change in activity after T cell subset depletion.

#### Flow cytometry with Intracellular Cytokine Staining (ICS)

Frozen PBMC taken at the same time points were stimulated with a single CSP or AMA1 megapool (rather than individual pools as in the ELISpot assay), each consisting of all the peptides contained within the peptide pools (Cp1-Cp9; Ap1-Ap12) for each antigen, using previously described methods [15]. Control stimulants were medium alone and the CEF peptide pool (Anaspec, San Jose, CA). Cells were phenotyped as CD4+ and CD8+ T cells and stained for IFN-γ, TNF and IL-2. Data for peptide pools were corrected for media response at each time point. A positive response was defined as a frequency of cytokine-stained CD4+ or CD8+ cells exceeding the geometric mean +3 standard deviations of the medium stimulated controls (0.03%).

### Statistical Analyses

A mixed linear model with compound covariance structure was used to compare geometric means between baseline and post-immunization antibody, ELISpot and ICS responses, adjusting comparisons between baseline and post-immunization using Dunnette’s method. All antibody responses were log_10_ transformed. Box plots [35] were used to display antibody and T cell responses. The lower quartile (25^th^ percentile), median and upper quartile (75^th^ percentile) are the base, transecting line and top of each box (defining the interquartile range or “likely range of variation”), and the upper and lower bars represent the maximum and minimum values unless outliers or suspected outliers are present (see figures). Suspected outliers and outliers are defined as exceeding 1.5 times and 3.0 times the interquartile range, respectively, above or below the box. When outliers are present, the bar is set at 1.5 times the interquartile range, leaving the outliers or suspected outliers beyond the bar. For the non-protected volunteers, suspected outliers and outliers are represented as open and filled dots, respectively. Vaccine efficacy was represented by a Kaplan-Meier plot and evaluated using log rank test. The Accelerated Failure Time model was used to determine the relationship between immune measures and time to parasitemia (delay in onset of parasitemia indicating partial protection), censoring the four fully protected volunteers on day 28. Tests for the relationship between immune measures and time to parasitemia were corrected for the 12 comparisons performed (Bonferroni correction). Rank correlations (Pearson Moment Correlations using log_10_ transformed values) determined the relationship between NAb and immunogenicity. Two-sided *p*<0.05 was considered significant in all tests.

## Results

### Study Flow

**Figure 3:**
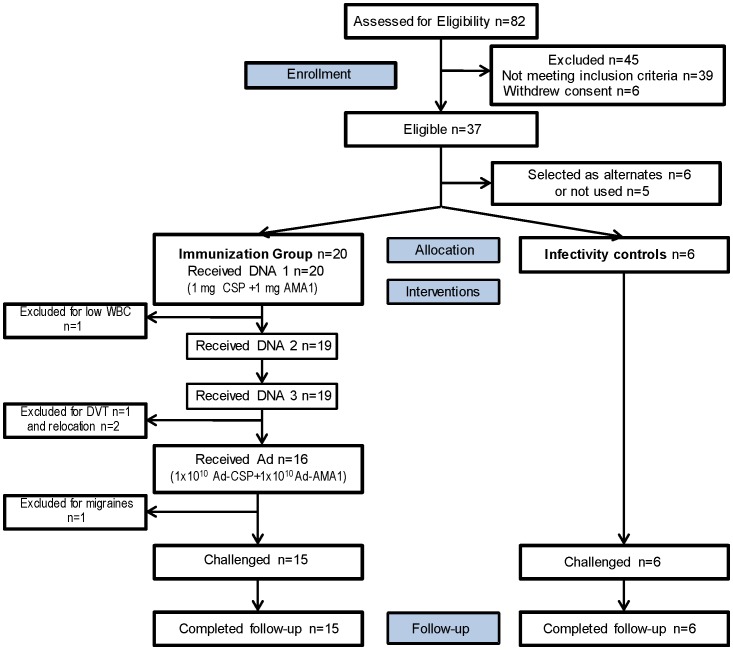
Flow diagram of immunized and control volunteers. Thirty-seven volunteers met all eligibility criteria and were allocated to the immunization group (n = 20) and infectivity controls (n = 6), and 11 were either alternates (n = 6) or not used. WBC = white blood count; DVT = deep venous thrombosis. See text for explanation.

Participant flow is shown in Figure 3. Recruitment for vaccine recipients took place at the WRAIR Clinical Trials Center between April – June 2009 and for infectivity controls between October – November 2009. Eighty-two healthy, malaria-naïve, civilian and military adult men and women, aged 18–50 years, were assessed for eligibility and 45 were excluded. The remaining 37 volunteers who met all screening criteria were assigned to the vaccine group (n = 20) and the infectivity controls (n = 6) (with eleven available as alternates or not used). The demographics of both groups were approximately balanced in gender, age and ethnic background (Table 1). Of the 15 volunteers in the immunized group who were challenged and whose immunogenicity data are presented here, six were negative for NAb (titer <12), four had low activity (12–500) and five had high activity (>500). Twenty volunteers received the first DNA immunization (Figure 3). One was excluded after the first DNA immunization due to a low neutrophil count (684/microliter) identified in a blood sample drawn immediately prior to the first DNA immunization (screening white blood counts having been normal), leading subsequently to a diagnosis of benign ethnic leukopenia; three were excluded after the third DNA immunization, two due to relocation unrelated to the trial and one for deep venous thrombosis (see Safety and Tolerability below); one was excluded after Ad administration due to migraine headache developing within a few hours of immunization and described as typical for this volunteer’s previously undisclosed migraine history. The low leukocyte count and history of migraines led to withdrawal of these two volunteers in order to adhere to the exclusion criteria, although both remained healthy. The volunteer with venous thrombosis was excluded due to the risks associated with anticoagulation during the period of recovery. Fifteen fully immunized volunteers and six infectivity controls underwent standardized controlled human malaria infection (CHMI) by bites of five *P. falciparum* (3D7) infected mosquitoes [36].

**Table 1:** Study subjects demographics.

	Immunized	Infectivity Controls
	n = 20	n = 6
**Male**	6 (30%)	3 (50%)
**Female**	14 (70%)	3 (50%)
**Age+standard deviation**	35+11.2	28+9.2
**African-American**	11 (55%)	4 (67%)
**Caucasian**	6 (30%)	2 (33%)
**Asian**	3 (15%)	0 (0%)
	**n = 15**	**n = 6**
**Ad5 neutralizing antibody**	<12, <12, <12, <12, <12, <12	
**titers**	45, 57, 169, 343	
	783, 1343, 1622, 1846, 2820	

Twenty volunteers were enrolled into the immunization group; five dropped out prior to CHMI (see Figure 3). Infectivity controls were enrolled later, in time for CHMI on week 28. NAb titers are provided for the 15 study subjects who were challenged (included in the immunogenicity analysis); these were measured just prior to Ad boost.

### Safety and Tolerability

All 58 DNA immunizations and 16 Ad immunizations in 20 volunteers were included in the safety analysis. During the seven days following each immunization, 155 solicited local and systemic adverse events (AE) were recorded as definitely, probably or possibly related to immunization (Table 2). Of these, 150 (96.8%) were mild (Grade 1) and four were moderate (Grade 2) and volunteers were able to perform daily activities). These AEs were erythema (36% of all AEs), induration (28%) and pain (21%) at the injection site, and fatigue (5%), headache (3%), diarrhea (2%), chills/rigors (1%), myalgia (1%), abdominal pain (1%) and nausea (1%). Only one AE (0.6%) was severe (Grade 3), preventing daily activities. This consisted of diarrhea starting a few hours after the first DNA immunization and ending within 48 hours, and was recorded as possibly related to immunization. No volunteer experienced the three other solicited AEs: objective fever, arthralgia or vomiting. There were nine transient laboratory test abnormalities deemed possibly related to immunization; these were asymptomatic and resolved spontaneously (Table 2).

**Table 2:** Numbers of volunteers experiencing local, systemic and laboratory adverse events (days 0–7 post each immunization).

Sign or Symptom	DNA 1 (n = 20)		DNA 2 (n = 19)		DNA 3 (n = 19)		Ad (n = 16)		Total AE’s
	(% of vol’s)		(% of vol’s)		(% of vol’s)		(% of vol’s)		(% of all AE’s)
	Gr1	Gr2/3	Gr1	Gr2/3	Gr1	Gr2/3	Gr1	Gr2/3	
**LOCAL**									
Pain/Tenderness	13(65)%)	0	7 (37%)	0	8 (42%)	0	5 (32%)	0	33(21%)
Erythema	14(70)%)	0	15(79%)	0	16(84%)	0	11(69%)	0	56(36%)
Induration/Swelling	9 (45%)	0	7 (37%)	0	17(89%)	0	10(63%)	0	43(28%)
**Total Local AEs**	**36**	**0**	**29**	**0**	**41**	**0**	**26**	**0**	**132(85%)**
**SYSTEMIC**									
Headache	1(5%)	0	1(5%)	0	1(5%)	0	1(6%)	1(6%)^1^	5(3%)
Fever	0	0	0	0	0	0	0	0	0
Chills/Rigor	0	0	0	0	0	0	2(13%)	0	2(1%)
Myalgia	0	0	1(5%)	0	0	0	0	1(6%)^1^	2(1%)
Arthralgia	0	0	0	0	0	0	0	0	0
Nausea	0	0	1(5%)	0	1(5%)	0	0	0	2(1%)
Vomiting	0	0	0	0	0	0	0	0	
Fatigue	4(20%)	1(5%)^1^	1(5%)	0	0	0	1(6%)	0	7(5%)
Diarrhea	1(5%)	1(5%)^2^	0	0	0	0	1(6%)	0	3(2%)
Abdominal pain	1 (5%)	1 (5%)^1^	0	0	0	0	0	0	2 (1%)
**Total Systemic AEs**	**7**	**3**	**4**	**0**	**2**	**0**	**5**	**2**	**23 (15%)**
**Total All AEs**	**43**	**3**	**33**	**0**	**43**	**0**	**31**	**2**	**155 (100%)**
**LABORATORY**									
Decreased platelets	1 (5%)								
Decreased WBC	1 (5%)		1 (5%)						
Elevated WBC	1 (5%)		1 (5%)		1 (5%)				
Elevated ALT			1 (5%)				1 (5%)		
Decreased Hb					1 (5%)				
**Total**	**3**		**3**		**2**		**1**		**9**

All local AE’s were considered definitely related to immunization, all systemic AE’s were considered probably related to immunization, except for diarrhea that was possibly related, and all laboratory AE’s were considered possibly related to immunization, Solicited local and systemic adverse events were recorded on days 0, 1, 2 and 7 and laboratory tests were recorded on days 0, 2, 7 and 28 after each immunization. Severity classification for signs and symptoms: Gr1 = adverse event does not interfere with daily activities; Gr2 = interferes with but does not prevent daily activities; Gr3 = prevents daily activities. Severity classification for decreased platelets: Gr1 = <lower limit of normal, >75,000/ul; decreased WBC: Gr1 = <lower limit of normal, >3,000/ul; elevated WBC: Gr1 = >upper limit of normal, <15,000/ul; elevated ALT: Gr1 = >upper limit of normal, <3 times upper limit of normal; decreased Hb: Gr1 = <lower limit of normal, >10.0 g/dL. All adverse events in the table are Gr1 (mild) unless noted otherwise.

1 = Gr2 (moderate);

2 = Gr3 (severe). All local adverse events occurred in the arm ipsilateral to the injection site.

Twelve unsolicited AEs (eight at the site of injection, four systemic) were recorded during the 28 days after each immunization and classified as definitely, probably or possibly related (Table 3). All resolved rapidly without sequelae.

**Table 3:** Number of volunteers experiencing unsolicited adverse events during 28 days following each immunization.

	Post DNA1	Post DNA2	Post DNA3	Post Ad	Total
	(n = 20)	(n = 19)	(n = 19)	(n = 16)	
**Unsolicited local adverse event**					
Bruise left arm injection site	Definite, Gr1				**1**
Bruise left arm injection site	Definite, Gr1				**1**
Bruise left arm injection site	Definite, Gr1				**1**
Right axillary pain	Probable, Gr1				**1**
Radiating pain down right arm	Definite, Gr1*	Definite, Gr1*			**2**
Bruise right arm injection site		Definite, Gr1			**1**
Right axillary furuncle			Possible, Gr1		**1**
**Total**	**5**	**2**	**1**	**0**	**8**
**Unsolicited systemic adverse event**					
Intermittent headaches	Possible, Gr1				**1**
Abdominal cramps		Possible, Gr1			**1**
Wheezing				Possible, Gr1	**1**
Subjective fever				Definite, Gr1	**1**
**Total**	**1**	**1**	**0**	**2**	**4**
**Total all AE’s**	**6**	**3**	**1**	**2**	**12**

Unsolicited adverse events were recorded on days 0, 1, 2, 7, 14 and 28 after each immunization. Severity classification: (a) bruising at the injection site: Gr1 = <5 cm in diameter; (b) all other signs and symptoms: Gr1 = adverse event does not interfere with daily activities; Gr2 = interferes with but does not prevent daily activities; Gr3 = prevents daily activities. All local adverse events occurred in the arm ipsilateral to the injection site. In addition to the AEs recorded in this table, there was one SAE recorded after the 3^rd^ DNA immunization – see text.

* The same volunteer experienced radicular pain immediately post jet injection for DNA1 and DNA2 but not DNA3.

In addition to the unsolicited AEs listed in Table 3, one serious and unexpected AE was reported six days after the third DNA immunization, a saphenous venous thrombosis leading to pulmonary embolism. This volunteer had been in good health with no significant past medical history. He experienced (but did not report) right calf tenderness starting six days after the third DNA immunization and two days after back-to-back round trips from Maryland to Maine (by car) and Florida (by plane). Six weeks later, he reported to the research team that he had been hospitalized due to a pulmonary embolism associated with leg swelling. He was withdrawn from further participation by the principal investigator, to assure the study subject’s safety.

### Efficacy

**Figure 4:**
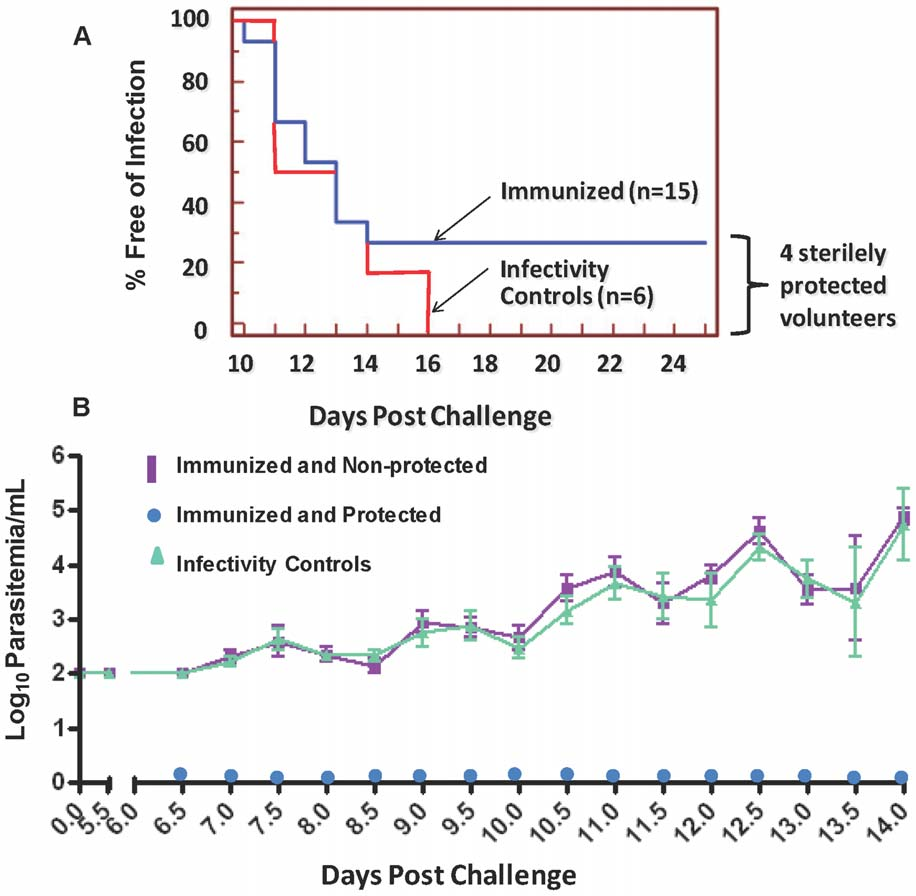
Development of parasitemia in the immunized and infectivity control subjects. **Panel A**: Parasitemia-free survival curves (Kaplan-Meier) for immunized volunteers and infectivity controls based on microscopic examination of peripheral blood smears. **Panel B**: Quantitative(q)-PCR measurements of parasitemia in immunized and challenge controls (error bars show standard deviation) (see reference 28).

**Figure 5:**
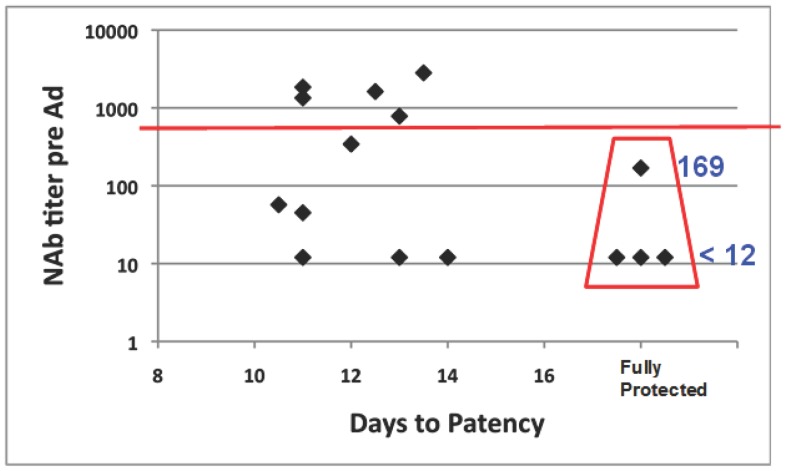
Pre-existing NAb to Ad5 may interfere with protection. Pre-existing NAb titers to Ad5 were measured prior to Ad immunization and are compared with days to patency by microscopy after CHMI. Three of six volunteers who were seronegative (NAb titer <12) (50%), and one of four subjects who were weakly positive (NAb titer 12–500) (25%) were protected (red box). All subjects with NAb titer >500 (above horizontal red line) became patent at a rate similar to subjects with NAb titer <500 (below horizontal red line).

All six infectivity controls developed parasitemia detected by qPCR between days 7 and 8 (mean 7.1) and by blood smear between days 11 to 16 (mean 12.3), indicating the mosquitoes were infective (Figure 4). Four of 15 immunized volunteers (v6, v10, v11 and v18) were fully protected, demonstrated by absence of parasitemia by qPCR [28] and by microscopic examination of Giemsa-stained thick smears (Figure 4). The remaining 11 immunized volunteers became parasitemic by qPCR between days 7 and 9 (mean 7.6) and by blood smear between days 10.5 to 14 (mean 12.1). The multiplication rate in non-protected immunized volunteers was similar to the multiplication rate in infectivity controls [28]. Likewise, non-protected immunized volunteers showed no evidence of delay in diagnosis by microscopy relative to infectivity controls. NAb titers were <1∶12 prior to immunization in protected volunteers v10, v11 and v18 and 1∶169 in protected volunteer v6; none of five volunteers with NAb titer >1∶500 was protected (Figure 5). Fisher’s exact test to investigate a potential inverse relationship between pre-existing NAb titer >1∶500 and protection was not statistically significant (p = 0.23).

### Immunogenicity

#### Antibody responses

**Figure 6:**
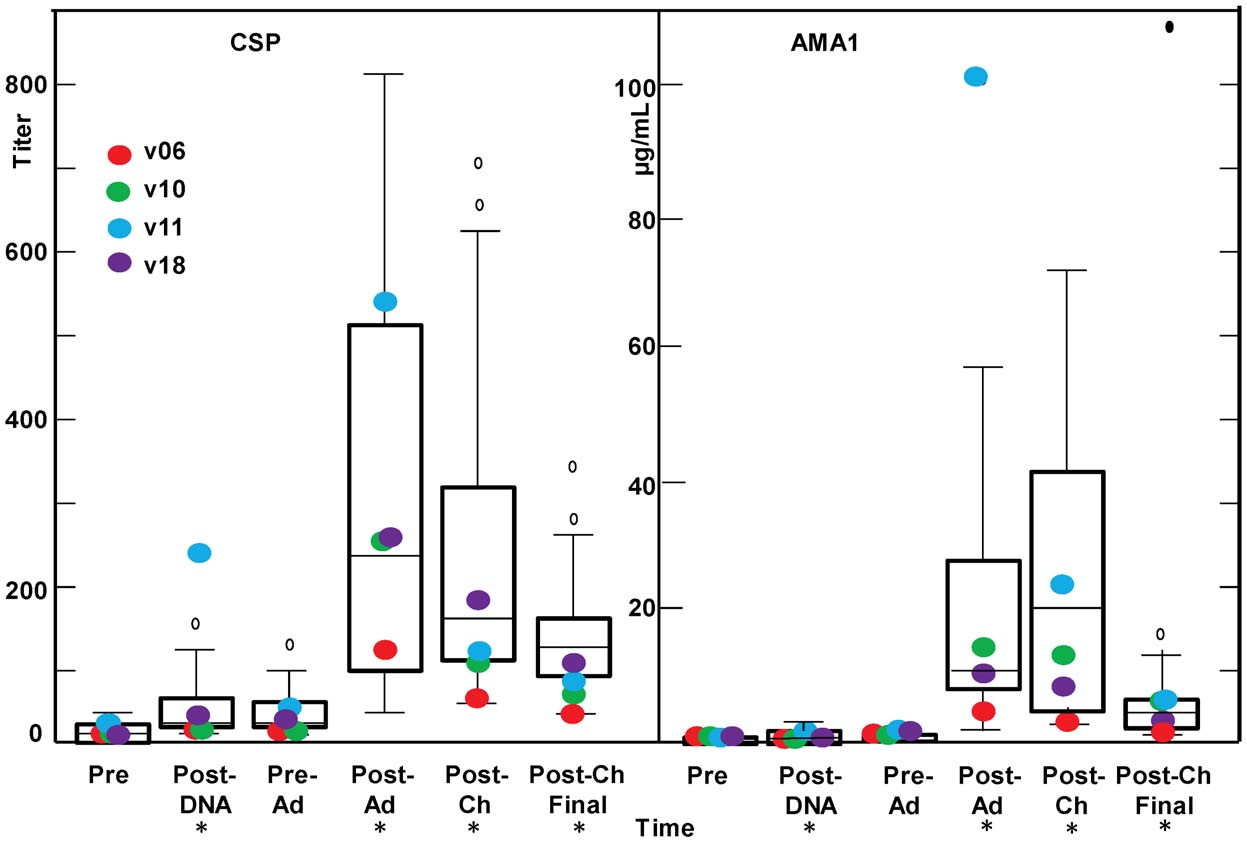
Antibody responses by ELISA to CSP and AMA1. The box plots (see Statistical Analysis section for description) represent anti-CSP titers and anti-AMA1 antibody concentration in µg/mL by ELISA for all 15 challenged volunteers. The time points on the x-axis are described in Figure 1. Four protected volunteers are shown as larger, color-coded dots. For the protected volunteers, the antibody titer to CSP of v11 post-DNA and the antibody concentration to AMA1 of v11 post-Ad are box plot outliers. Group geomean CSP and AMA1 ELISA activities for the fifteen recipients were significantly higher than baseline (*) post-DNA, post-Ad, post-Ch and post-Ch final relative to pre-immunization levels (p = <0.0001, mixed linear model).

Antibody responses (Figure 6) measured by ELISA to CSP and AMA1 rose significantly but were still negligible 28 days after DNA immunization (geometric mean CSP titer 28, range <10–253; AMA1 0.24 µg/mL, range 0.04–2.42 µg/mL). Antibody responses increased further following Ad immunization but were still relatively low (CSP 210, range 44–817; AMA1 11.9 µg/mL, range 1.5–102 µg/mL). Sporozoite IFA responses were also low post-DNA (data not shown) and post-Ad (geomean titer 160, range 10–1280). No growth inhibition was seen when purified immunoglobulin from volunteer sera was added to *P. falciparum* blood stages in culture (<15% inhibition, data not shown). There was no statistical association between antibody levels and protection for CSP and AMA1 (Bonferroni corrected), although the volunteer with the highest anti-AMA1 antibody concentration at the time of challenge (v11, 102 µg/mL) was protected (Figure 6, blue dot).

### *Ex vivo* T cell IFN-γ Activities by ELISpot for CSP and AMA1

**Figure 7:**
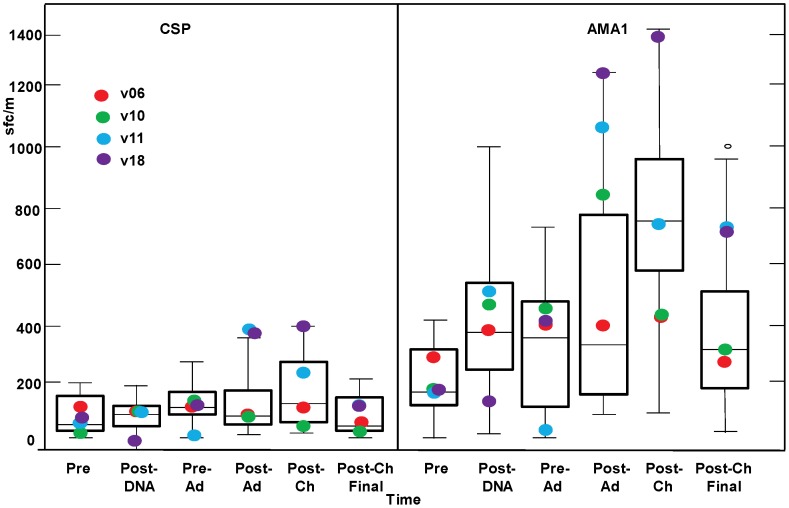
*Ex vivo* T cell IFN-γ activities by ELISpot Assay for CSP and AMA1. The box plots (see Statistical Analysis section for description) represent CSP and AMA1 IFN-γ ELISpot responses (summed peptide pool-specific responses) in spot forming cells per million PBMCs for all 15 challenged volunteers. The time points on the x-axis are described in Figure 1. For the protected volunteers, the IFN-γ ELISpot responses to CSP of v11 and v18 post-Ad are box plot suspected outliers. Group geomean CSP and AMA1 IFN-γ ELISpot activities for the fifteen recipients were not significantly higher than baseline post-DNA, pre-Ad, post-Ad, post-Ch or post-Ch final relative to pre-immunization levels (CSP p = 0.057, AMA1 p = 0.16, mixed linear model).

Group geomeans of the summed ELISpot responses to individual peptide pools to CSP (Figure 7) at pre-immunization (63 sfc/m, range 13–209 sfc/m), post-DNA (70 sfc/m, range 3–197 sfc/m), pre-Ad (100 sfc/m, range 6–279 sfc/m) and post-Ad (86 sfc/m, range 13–408 sfc/m) were similar and not significantly higher than pre-immunization levels, indicating that many volunteers did not respond to the vaccine regimen. Four of the 15 volunteers met ELISpot positivity criteria for CSP at the post-Ad time-point (six days prior to challenge), with the two highest responses in protected v11 (408 sfc/m) and v18 (398 sfc/m), whereas protected v6 (80 sfc/m) and v10 (55 sfc/m) were negative (Figure 7). When ELISpot responses to AMA1 were measured, one volunteer (v03) had very high activity at pre-immunization (4110 sfc/m) that declined after DNA and Ad immunizations to within the ranges of other volunteers; the reason for this activity is not known. Geomeans of ELISpot responses to AMA1 (Figure 7) pre-immunization (154 sfc/m, range 18–421 sfc/m, excluding v03), post-DNA 295 sfc/m, range 6–1009 sfc/m), pre-Ad (243 sfc/m, range 13–733 sfc/m) and post-Ad (348 sfc/m, range 88–1270 sfc/m) were similar, as shown for CSP (Figure 7). However, geomean ELISpot activities to AMA1 were higher than to CSP at pre-Ad (243 sfc/m vs. 100 sfc/m) and post-Ad (348 sfc/m vs. 86 sfc/m) (Figure 7). Twelve of 15 individual volunteers met positivity criteria at the post-Ad time-point for AMA1, with the three highest responses in protected v10 (810 sfc/m), v11 (1046 sfc/m) and v18 (1270 sfc/m); protected v6 was also positive (312 sfc/m). ELISpot responses to AMA1 were significantly associated with protection (p = 0.019), while responses to CSP were not (p = 0.23) (Bonferroni corrected).

### ELISpot Depletion Studies

ELISpot depletions were performed at post-Ad with the four protected and three of the non-protected volunteers (Table 4). CSP responses were reduced after depletion of CD4+ and CD8+ T cells (v11 protected and v03 non-protected), CD4+ T cells only (v12 non-protected) and CD8+ T cells only (v18 protected). AMA1 responses were reduced after depletion with CD4+ and CD8+ T cells (v10 and v11 protected, v12 and v15 non-protected), CD4+ T cells only (v06 protected) and CD8+ T cells only (v18 protected). Therefore protected volunteers v10 and v11 developed a CD4+ and CD8+ T cell dependent response to AMA1 (v10) or to both CSP and AMA1 (v11), v18 developed a CD8+ dependent response to CSP and AMA1, and v06 developed a CD4+ dependent response to AMA1. The frequencies of CD4+ and CD8+ T cell responses were more fully quantified using flow cytometry.

**Table 4:** IFN-γ ELISpot Assay: Depletion of CD4+ and CD8+ T cells.

	Protected Volunteers									Non-protected Volunteers					
		v06		v10		v11		v18		v03		v12			v15
		CD4	CD8	CD4	CD8	CD4	CD8	CD4	CD8	CD4	CD8	CD4	CD8	CD4	CD8
**CSP**						−100	−80	−17	−76	−74	−99	−97	−15		
**AMA1**		−91	+76	−94	−83	−99	−90	+2	−94	−100	+247	−100	−51	−81	−63
		−94	−12	−82	−66	−59	−49								
						−78	−70								

Protected volunteers v06, v10, v11 and v18, and non-protected volunteers v03, v12 and v15 were tested for ELISpot activity to CSP and AMA1 peptides after depletion of CD4+ or CD8+ T cells. Percent reduction in the number of spot forming cells per 1,000,000 PBMC following depletion of CD4+ T cells or CD8+ T cells is shown for each volunteer tested. A positive effect was defined as >20% reduction (See Reference 15). v06 and v10 were tested twice with AMA1, and v11 was tested three times with AMA1.

### Total IFN-γ T cell Responses by Flow Cytometry/intracellular Cytokine Staining (ICS) for CSP and AMA1

**Figure 8:**
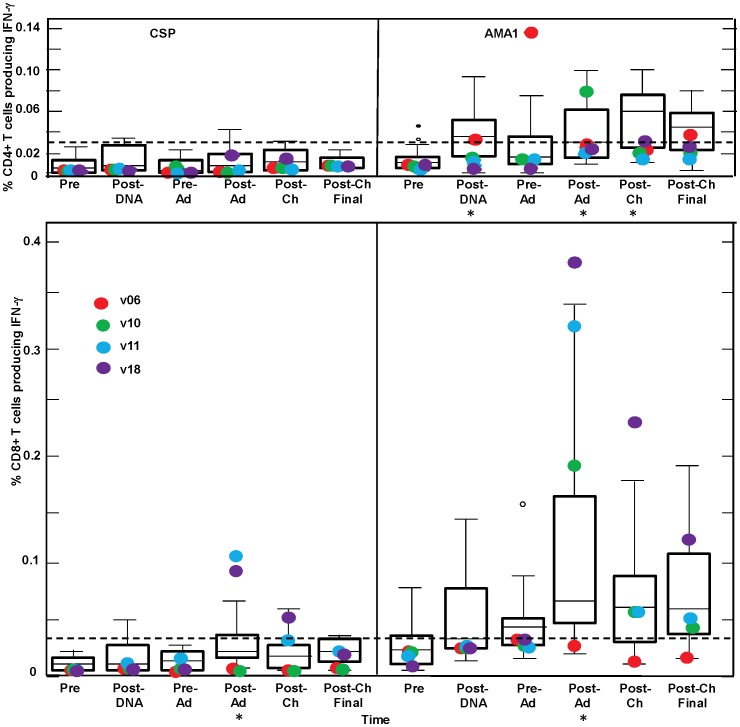
IFN-γ activities by flow cytometry for CSP and AMA1. The box plots (see Statistical Analysis section for description) represent IFN-γ -producing CD4+ or CD8+ T cell frequencies as percentage of gated CD4+ or CD8+ T cells, measured by flow cytometry assays after stimulation with a single CSP or AMA1 megapool containing all individual peptide pools for each antigen, for all 15 challenged volunteers. The time points on the x-axis are described in Figure 1. The four protected volunteers are shown as larger, color-coded dots. For the protected volunteers, the CD4+ T cell AMA1 response of v06 at pre-Ad is a box plot outlier, and the CD8+ T cell CSP responses of v11 and v18 post-Ad, and the CD8+ T cell AMA1 responses of v18 post-Ad and post-Ch are box plot suspected outliers. The dotted lines represent positive cutoff (0.03% as described in Methods). IFN-γ -producing CD4+ T cell frequencies were significantly higher than baseline (*) post-DNA (p = 0.047), post-Ad (p = 0.0097) and post-Ch (p = 0.004) for AMA1 (mixed linear model). IFN-γ -producing CD8+ T cell frequencies were significantly higher than baseline (*) post-Ad for CSP (p = 0.007) and post-Ad for AMA1 (0.002) (mixed linear model).

Total CD4+ or CD8+ T cell IFN-γ responses were measured. This included IFN-γ +IL2+TNF+, IFN-γ +IL2-TNF+, IFN-γ +IL2+TNF-, and IFN-γ +IL2-TNF- containing cells (Figure 8). As in previous studies of Ad vaccines [15],[37],[38] both CD4+ and CD8+ T cell IFN-γ responses were induced. CD4+ T cell responses to CSP were positive with only one non-protected volunteer at the post-Ad time point. CD8+ T cell responses to CSP were positive with 3 volunteers at the post-Ad time point with protected v11 and v18 showing the highest responses (0.10% and 0.09% of gated CD8+ T cells, respectively), similar to ELISpot responses.

Positive responses to AMAI were more frequent than to CSP. Positive CD4+ T cell responses developed in six volunteers pre-Ad and eight volunteers post-Ad, where the highest response was with a non-protected volunteer. Positive CD8+ T cell responses developed in five volunteers post-DNA, eight volunteers pre-Ad, and 12 volunteers post-Ad where CD8+ T cell responses, like ELISpot responses, were highest in protected v10, v11 and v18 (0.18%, 0.32% and 0.38%, respectively) (Figure 8). The largest component of the CD8+ T cell IFN-γ response was single IFN-γ -secreting cells (IFN-γ+IL2- TNF-α-) for both CSP (v11 and v18, 100% and 96% respectively) and AMA1 (v10, v11 and v18, 66%, 70% and 95% respectively).

CD8+ T cell responses to AMA1 were associated with protection (p = 0.0492 Bonferroni corrected), while no association was seen for CSP (p = 0.23 Bonferroni corrected). In contrast, IFN-γ production by CD4+ T cells showed no statistical association with protection for either antigen (p = 0.99 for AMA1, p = 0.99 for CSP, Bonferroni corrected). However, v6, the only protected volunteer not showing strongly elevated CD8+ T cell IFN-γ responses to CSP or AMA1 at post-Ad, exhibited the highest CD4+ IFN-γ and IL2 T cell responses to AMA1 found in the trial, at the pre-Ad time point (0.14% for IFN-γ Figure 8; 0.23% for IL2, data not shown).

### Effect of Pre-existing NAb on ELISA, ELISpot and ICS Activity

Rank correlations were used to examine the relationship of NAb titers measured prior to Ad immunization and immunogenicity (IFN-γ ELISpot, total IFN-γ CD4+ T cell ICS, total IFN-γ CD8+ T cell ICS, ELISA, sporozoite IFA) (Table 5). The statistical power to identify associations was limited in this small study. NAb did not show significant correlations with any immune measures except for showing a negative association with AMA1 ELISA (p = 0.038). Trends were observed for negative associations with CSP and AMA1 ELISpot responses (p = 0.053, 0.102, respectively) and for IFA (p = 0.078).

**Table 5:** Rank correlations between pre-existing anti-Ad5 NAb titers and ELISpot, CD4+ T cell and CD8+ T cell IFN-γ activities, ELISA and Sporozoite IFA titers.

	Immune Measure Correlations								
	ELISpot		CD4		CD8		ELISA		IFA
	CSP	AMA1	CSP	AMA1	CSP	AMA1	CSP	AMA1	SPZ
**r**	−0.51	−0.44	0.194	−0.22	−0.24	−0.058	0.013	−0.54	−0.468
**p**	0.053	0.102	0.489	0.428	0.395	0.837	0.962	**0.038**	0.078

Pre-existing Ad5 NAb titers measured just prior to Ad immunization were tested for negative correlations with CSP and AMA1 activities by IFN-γ ELISpot, total IFN-γ CD4+ T cell ICS, total IFN-γ CD8+ T cell ICS, ELISA and sporozoite IFA for all volunteers (n = 15). r = rank correlation coefficient, and p = p value for the null hypothesis that the correlation is zero (two-tailed). Significant correlations are shown in bold.

## Discussion

### Interpretation

Gene-based vaccines have been used for years in veterinary medicine to protect multiple animal species against a variety of pathogens [39],[40],[41]. In contrast, with one exception, no gene-based vaccines have been licensed for human use, despite their potential for inducing strong CMI against diseases lacking effective vaccines, such as HIV/AIDS, tuberculosis and malaria. Adenovectors have proven especially effective at inducing robust CD8+ T cell responses [37],[42],[43]. In this first clinical assessment of a DNA/Ad prime boost regimen for malaria, we induced sterile immunity in four of 15 research subjects that was significantly associated with IFN-γ ELISpot responses to one of the vaccine antigens, AMA1 (p = 0.019, Bonferroni corrected). The second antigen (CSP) likely contributed to protection in two of the volunteers but did not show a significant association in the group as a whole (p = 0.23, Bonferroni corrected). The association between IFN-γ responses to AMA1 was also found when CD8+ T cells were examined (p = 0.0492, Bonferroni corrected), but not CD4+ T cells. In contrast, antibody responses showed no association with protection.

To our knowledge, there are no examples of licensed human vaccines thought to protect solely on the basis of CMI [44]; thus our trial may be the first to demonstrates the feasibility of targeting human pathogens solely by this mechanism. It also provides support for a subunit approach to reproduce the high grade immunity induced by irradiated sporozoites, genetically-attenuated sporozoites or intact sporozoites co-administered with chloroquine, all of which appear to rely primarily on CMI as the mechanism of protection.

While all four protected volunteers developed high levels of cell-mediated immunity (CD8+ T cells in three, CD4+ T cells in one), responses were lower in the eleven non-protected volunteers. This may explain why there was no delay to parasitemia in the immunized, non-protected volunteers, contrasting to what has been seen in other malaria vaccine trials [45]. This may reflect differences in protective mechanisms, or it could be that in this small trial, due to genetic restriction, no volunteers developed responses of intermediate magnitude to the protective epitopes and for this reason no delays in the onset of parasitemia were seen. However, all non-protected volunteers shared at least one HLA A or B supertype allele with protected volunteers (data not shown). Supplementing the vaccine with additional antigens could address this potential limitation, by increasing the likelihood of HLA-malaria peptide matches. Alternatively, protective immunity may have been suppressed in non-protected volunteers by regulatory T cell responses [46], which were not measured in this trial.

Five of the non-protected volunteers had high pre-existing NAb (titer >1∶500). While this association was not statistically significant, and pre-existing immunity only marginally adversely affected AMA1 ELISA responses (p = 0.04), the trend is concerning for adults since prior exposure to wild type adenovirus 5 is common in the USA and developing countries [47]. However, NAb titers are low in six-to-twelve month-old infants including in sub-Saharan Africa [48], providing a vaccination window early in life when neutralizing antibodies should have minimal impact.

Importantly, both DNA and Ad components of the vaccine appeared safe and well tolerated, with primarily mild adverse events observed. The one serious adverse event (a saphenous venous thrombosis after extended travel) appeared unlikely related to immunization. A migraine headache in another subject, possibly precipitated by Ad administration, was consistent with prior history of migraines (unreported at the time of screening). While most clinical trials of gene-based vaccines have concluded that they are safe, in one trial of an Ad5-vectored HIV vaccine, there was an increased risk of HIV infection in Ad5-seropositive, uncircumcised men engaging in HIV risk behaviors [49]. Such observations underscore the importance of ongoing safety monitoring.

### Generalizability

This study supports the potential value of the DNA/Ad prime-boost strategy to induce protective cell mediated responses, particularly CD8+ T cells critical for killing intracellular pathogens, and thus its application to other infectious diseases particularly tuberculosis [50] and HIV [51],[52],[53] where CMI is likely important.

### Limitations

The main finding of this study that CD8+ T cell responses to AMA1 and potentially CSP are associated with protective efficacy, may not apply to other malaria antigens. A second limitation is the generally poor antibody responses, confirming earlier studies by our laboratory when the Ad vaccines were used alone [15],[16] and suggesting that although priming with DNA induced protection it did not improve antibody responses. As previously discussed [15], it may be difficult to induce both humoral and cellular responses using this prime-boost combination. In addition, CD8+ T cell responses were low in the majority of vaccinees. Finally, pre-existing NAb were associated with reduced antibody responses to AMA1 and cellular ELISpot responses to both antigens, but not CD8+ T cell responses detected by flow cytometry, and this was consistent with protection only being achieved when NAb titers were <500. Whether Ad5 vectors could achieve protection in infants and young children who are largely seronegative, or whether alternative Ad vectors will be required, remains to be investigated.

### Overall Evidence

In summary, our trial demonstrated the sterile protection of 27% of volunteers against *P. falciparum* malaria, the highest protection achieved in humans against a parasite using a gene-based vaccine. Protection was associated with IFN-γ ELISpot and CD8+ T cell responses to AMA1, with possible contribution by CD4+ T cell responses to AMA1 and CD8+ T cell responses to CSP. Approaches to strengthen the vaccine include adding antigens to broaden HLA coverage, electroporating the DNA to improve cellular uptake, substituting highly immunogenic adenovectors derived from non-prevalent serotypes to avoid NAb, formulating DNA and/or Ad in adjuvants, including plasmids or adenovectors encoding immunomodulatory cytokines, or using prime-boost combinations with other vaccine platforms such as recombinant proteins to strengthen antibody responses. We have prioritized adding a third antigen and testing alternative rare-serotype adenovectors as the next steps in development.

## Supporting Information

Checklist S1 CONSORT Checklist.
(DOC)

Protocol S1 Trial Protocol.
(DOC)
