# Supplementary material for: DNA Prime/Adenovirus Boost Malaria Vaccine Encoding P. falciparum CSP and AMA1 Induces Sterile Protection Associated with Cell-Mediated Immunity
Source: PLoS One. 2013 Feb 14;8(2):e55571. doi: 10.1371/journal.pone.0055571 (PMC3573028; doi:10.1371/journal.pone.0055571)
Supplement: Protocol S1 — Trial Protocol. (DOC) [file pone.0055571.s002.doc]

CLINICAL TRIAL PROTOCOL

**Clinical Trial on Safety, Immunogenicity, and Efficacy of a Prime Boost Regimen of DNA- and Adenovirus-vectored Malaria Vaccines Encoding *Plasmodium falciparum* Circumsporozoite Protein and Apical Membrane Antigen 1 in Malaria-Naïve Adults in the United States**

| IND Number | BB-IND 13977, approved on 07 APR 09 |
| --- | --- |
| Sponsor | Office of the Surgeon General of the Army (OTSG)  Department of Defense (DoD)  5109 Leesburg Pike, Suite 672  Falls Church, Virginia 22041-3258 |
| Institutional Review Board of Record (IRB) | Walter Reed Army Institute of Research (WRAIR) IRB, Division of Human Subject Protection (DHSP) |
| Other Human Subject Review Boards | 1. Human Subjects Research Review Board (HSRRB), Human Research Protection Office (HRPO), United States Army Medical Research and Materiel Command (USAMRMC) 2. Naval Medical Research Center (NMRC) IRB |
| Protocol Number | WRAIR IRB # 1550; HRPO #A-15350; NMRC.2009.0004 |

| Principal Investigator | Ilin Chuang, MD, MPH  Commander (CDR), Medical Corps (MC), US Navy (USN)  Principal Investigator  U.S. Military Malaria Vaccine Program (USMMVP)  Naval Medical Research Center/Walter Reed Army Institute of Research (NMRC/WRAIR)  503 Robert Grant Avenue, 3W41  Silver Spring, MD 20910-7500  301-319-9889 (NMRC)  [ilin.chuang@med.navy.mil](mailto:ilin.chuang@med.navy.mil) |
| --- | --- |

| Sponsor and Sponsor’s Representative | Office of the Surgeon General of the Army (OTSG)  Department of Defense (DoD)  5109 Leesburg Pike, Suite 672  Falls Church, Virginia 22041-3258  Robert E. Miller, Ph.D., RAC  Director, Division of Regulated Activities and Compliance  OTSG Sponsor's Representative  U. S. Army Medical Materiel Development Activity  1430 Veterans Drive Fort Detrick, MD 21702  Telephone: 301-619-0042  Fax: 301-619-0197 |
| --- | --- |

| **Sponsor’s Medical Expert** | Thomas L. Richie, Captain (CAPT), MC, USN |
| --- | --- |

| **Regulatory Affairs** | United States Army Medical Materiel Development Activity (USAMMDA)  1430 Veterans Drive, Ft Detrick, MD 21702-9232  Telephone: 301-619-7645, DSN: 343-7645  USMMVP  Santina Maiolatesi 301-319-7314 |
| --- | --- |

| **Regulatory Monitor** | Anna Marie Seale  Clinical Operations and Quality Branch, USAMMDA  1430 Veterans Drive, Ft. Detrick, MD 21702-9232  Telephone: 301-619-3774  Fax: 301-619-0371 |
| --- | --- |

| **Medical Monitor** | Arthur Lyons, MD, PhD, MC, USA  Chief, Clinical Research Unit  Division of Viral Diseases  Walter Reed Army Institute of Research  503 Robert Grant Ave.  Silver Spring, MD 20910  301-319-9021 |
| --- | --- |

| **Associate Investigators** | *Clinical Investigators*  Thomas L. Richie, CAPT, MC, USN, USMMVP, NMRC  Christian F. Ockenhouse, Colonel (COL), MC, US Army (USA), USMMVP, WRAIR  Cindy Tamminga, CDR, MC, USN, USMMVP, NMRC  Michele Spring, MD, MSPH, USMMVP, WRAIR  Judith Epstein, CDR, MC, USN, USMMVP, NMRC  Mark Polhemus, LTC, MC, USA, USMMVP, WRAIR  James Cummings, LTC, MC, USA, Clinical Trials Center (CTC), WRAIR  Jason Bennett, Cpt, MC, USA, BAMC  Melanie L. Guerrero, LTC, MC, USA, CTC, WRAIR  *Laboratory Investigators*  Martha Sedegah, PhD, USMMVP, NMRC  Noelle Patterson, MS, USMMVP, NMRC  Student Investigators  Marla Brunell, MAJ, USMMVP, NMRC  Kari Hunter, CPT, USMMVP, NMRC |
| --- | --- |

| **Trial Location:** | WRAIR Clinical Trials Center, Silver Spring, MD |
| --- | --- |

| **Challenge Location** | Walter Reed Army Institute of Research Insectary  503 Robert Grant Avenue  Silver Spring, MD 20910-7500  Telephone: 301-319-9557 |
| --- | --- |

| Clinical Laboratory | Quest Diagnostic Inc. |
| --- | --- |

| Research Laboratory | U.S. Military Malaria Vaccine Program  503 Robert Grant Avenue  Silver Spring, MD. Phone: 301-319-7586 |
| --- | --- |
|  | Ad 5 neutralizing antibody titer  Bob Bailer, Ph.D., NVITAL/HJF  Vaccine Immunogenicity Testing Program, Director Vaccine Research Center-NIH/NIAID 40 Convent Drive, MSC 3022 Building 40, Room 3508 Bethesda, MD 20892  Telephone: 301-594-8481  Telephone: 301-208-6813 (NVITAL) |
|  | HLA Typing  Dr. Jennifer Ng, C. W. Bill Young/DoD Marrow Program  11333 Woodglen Drive  Rockville, MD 20852  Telephone: 301-998-8900 |

| Study Conduct: | The study will be conducted according to the protocol and in compliance with International Conference on Harmonization (ICH), Good Clinical Practice (GCP), and other applicable regulatory and Department of Defense (DoD) requirements. |
| --- | --- |

| **Protocol Approval:** | SRC, conditional approval, 16JAN2009; final approval, 30JAN09  IRB, conditional approval, 11 FEB 09  HRPO, conditional approval, 25 FEB 09 |
| --- | --- |

| **Protocol History:** | Version 1.0, to SRC on 5 JAN 09  Version 2.0, to IRB on 21 JAN 09  Version 3.0, to IRB on 10 MAR 09  Version 3.1, to IRB on 25 MAR 09  Version 4.0, amendment # 2, 29 APR 09  Version 4.0, amendment # 2 with response to FDA comments, 06MAY09  Version 4.1, amendment #4, 15 JUL 09 |
| --- | --- |

**FOR OFFICIAL USE ONLY** Information and data included in this protocol contain privileged and or proprietary information, which is the property of the United States Army. No person is authorized to make it public without express written permission of the United States Army. These restrictions on disclosure will apply equally to all future information, which is indicated as privileged or proprietary.

**STUDY SYNOPSIS**

Title of Study

Clinical Trial on Safety, Immunogenicity, and Efficacy of a Prime Boost Regimen of DNA- and Adenovirus-vectored Malaria Vaccines Encoding *Plasmodium falciparum* Circumsporozoite Protein and Apical Membrane Antigen 1 in Healthy Malaria-Naïve Adults in the United States

**Study Site**

WRAIR Clinical Trials Center. Challenges will be conducted at the WRAIR Insectary (WRAIR/NMRC Building 503) Silver Spring, MD.

Period of Trial

The projected trial period spans from the start of recruitment (estimated March 1, 2009) through the last follow-up contact with the last subject five years from the time of first immunization.

Study Objectives

**Primary Objectives**

- Assess the safety of a heterologous prime-boost vaccine regimen (DNA-Ad) consisting of a DNA vaccine prime (NMRC-M3V-D-*Pf*CA) and adenovirus type 5 vaccine boost (NMRC-M3V-Ad-*Pf*CA) in healthy malaria-naïve adults

**Secondary Objectives**

1. Assess the protective efficacy against sporozoite challenge with Plasmodium falciparum (*Pf*), 3D7

2. Assess the humoral immunogenicity of this prime-boost regimen to CSP and AMA1 antigens by enzyme linked immunosorbent assay (ELISA)

3. Assess the cellular immunogenicity of this prime-boost regimen to CSP and AMA1 antigens by enzyme linked immunospot assay (ELISpot) and intracellular staining (ICS)

**Exploratory Objectives**

1. Measure host immune response by using oligonucleotide microarrays to assess breadth and magnitude of immune response
2. Measure the functional humoral immune response by Growth Inhibition Assay (GIA),
3. Analysis of immunofluorescence assay (IFA) titers against sporozoites and blood stage parasites
4. To determine if a specific antibody or cellular response, or gene expression profile (as assessed by microarray analysis) correlates with protection
5. Compare the safety, immunogenicity, and protective efficacy between groups with and without pre-existing neutralizing antibodies against adenovirus type 5 (Ad5+/Ad5-)
6. Assess the Ad5 serology kinetics in both Ad5+ and Ad5- subjects following immunization with Ad

**Primary Endpoints and Assessment of Endpoints**

- - - 1. The vaccine will be considered safe and well-tolerated if there are no serious adverse events (SAE) related to vaccine administration or if any severe events are relatively benign (e.g. erythema meeting criteria for severe due to its dimensions but not significantly affecting the activities of daily living for the subject) or brief in duration (e.g. less than 48 hours), and the same event occurring in no more than 20% of the volunteers. The AEs will be assessed according to the method below.

a. Occurrence, severity, and duration of any solicited symptoms starting on the day of

immunization through day 7 after each immunization

b. Occurrence of any unsolicited symptoms, abnormal physical findings, and laboratory

values starting from the day of immunization through day 28 after each immunization

c. Occurrence of any SAEs, as defined in 21 CFR 312.32, during the five-year study period

**Secondary Endpoints**

1. The DNA-Ad vaccine is considered efficacious if it offers any degree of sterile or partial protection that reaches statistical significance (p<0.05). The vaccine efficacy will be determined by two parameters: 1) sterile protection for 28 days after challenge, 2) partial protection as determined by delay to parasitemia by thick smears and PCR. More specifically, the degree of sterile protection will be assessed by comparing the number of immunized subjects who did not develop malaria infection after 28 days of challenge and the control subjects over the same time period. The presence of partial protection will be determined by observance of statistical difference between the timing of the onset of parasitemia between immunized and control subjects.
2. The DNA-Ad vaccine is considered to confer humoral immunity if the increase of antibody response for CSP or AMA1 by ELISA post immunization is statistically significant (p<0.05) compared to the levels before immunization.
3. The DNA-Ad vaccine is considered to confer cellular immunity if a positive ELISpot response is detected after immunization which will have the following 3 characteristics: (1) a statistically significant difference between the number of spot forming cells in triplicate test wells and triplicate control wells; (2) at least a doubling of spot forming cells in test wells relative to control wells; and (3) a difference of at least 5 spots between test and control wells.

**Exploratory Endpoints**

1. Evidence of vaccine effect on prepatent period as determined by PCR and thick blood smear collected at closely spaced intervals at least daily from day 6 post challenge to the last of the three consecutive negative smears.
2. Evidence of vaccine effect on rate of development of blood stage parasites as determined by PCR and thick blood smears collected at closely spaced intervals at least daily from day 6 post challenge to the last of the three negative smears.
3. Measurement of host immune response using oligonucleotide microarrays to assess breadth and magnitude of immune response
4. Measurement of the degree of functional antibody by GIA
5. Measurement of antibody response by IFA
6. Determination of correlates of protection are made by analyzing 1) antibody titers, 2) ELISpot responses, 3) gene expression profile to known malaria antigens after challenge to observe if there are parameters with a positive association with protection, and the extent of such associations.
7. Compare safety, immunogenicity and vaccine efficacy between the Ad5 + and Ad5 – groups to see if there is any degree of difference that reaches statistical significance
8. Ad5 kinetics following immunization will be characterized by descriptive analysis of Ad5 titers in both Ad5 + and Ad5 – groups after the Ad5 dose is given

Study Design

This study is an open-label, multi-centered Phase 1/2a study designed to assess the safety, immunogenicity, and efficacy of a DNA-Ad vaccine in healthy adults who are Ad5 seropositive or seronegative. The vaccinated study group will consist of up to 20 healthy, malaria-naïve adults aged 18 to 50 years, who have been previously screened to meet inclusion and exclusion criteria and will receive three priming doses of DNA at 0, 4, and 8 weeks and a single dose of the boosting component, Ad, at 24 weeks. Follow up visits will occur 1, 2, 7, 14, and 28 days after each immunization. The control group who were screened according to the same inclusion and exclusion criteria consists of six non-immunized subjects that will participate in a challenge to assure that vaccinated subjects were indeed exposed to *P. falciparum*. At around 28 weeks (2-4 weeks after the Ad dose), subjects in both the immunized and control cohorts will receive malaria challenge. Subjects will be assessed for development of parasitemia by blood smears at least daily from day 6 to day 19 and will be closely observed in hotel from day 10 to day 19. Subjects will then be followed periodically and have the final in-person visit twelve weeks after the challenge, followed by annual contact by phone, email, or mailings up to five years after the first dose of immunization per FDA recommendation.

| Group  (N) | Week  **Ad 5^§^**  **Status** | 0 | 4 | 8 |  |  | 24 | 28 |
| --- | --- | --- | --- | --- | --- | --- | --- | --- |
| 1 (20) | Ad 5 (-/+) | 2 mg  DNA | 2 mg  DNA | 2 mg  DNA |  |  | 2x10^10^ *pu*  Ad | sporoz.  challenge |
| Infectivity Controls (6) | | | | | | | | |

Ad5 titer is negative, “-“ if < 500 and positive, “+” if > 500.

**Study Population**

Males and females between ages of 18-50, who are healthy and from any geographic location will be actively recruited primarily from the local national capital area.

**Number of Subjects**

The total number of subjects to be enrolled is up to 26 as in the table below. The immunized group will consist of up to 20 but no fewer than 16 subjects on the day of the first DNA immunization. An additional six subjects will serve as alternates and will only be enrolled as needed.

|  | Planned N | Alternates |
| --- | --- | --- |
| DNA-Ad vaccine | 20 | 3 |
| Control | 6 | 3 |

**Duration of Subject Participation**

Each immunized subject will actively participate up to approximately 52 weeks (screening, immunization, challenge, and follow-up). Annual telephone/email/mail follow-ups will occur for up to 260 weeks from the time of first immunization. Control subjects will participate up to 24 weeks including screening, challenge, treatment, and follow-up.

**Test Article**

The test article consists of three doses of DNA (lot number 11040330), the priming portion of the vaccine, followed by one dose of Ad (NMRC-MV-Ad-*Pf*C, lot number FPCSP-002 in 1:1 blend with NMRC-MV-Ad-*Pf*A, lot number FPAMA1-002), the boosting portion of the vaccine.

**Drug Form, Route of Administration, and Dose Regimen**

The DNA portion will be given as a 2 mg total dose (1 mg per construct in a volume of 1 mL) as two intramuscular (IM) injections, one in each arm, via Biojector® at 0, 4, and 8 weeks. This will be followed by the Ad portion of the vaccine which is a total of 2 x 10^10^ particle units (*pu*) (1 x 10^10^ *pu* per each of 2 constructs including CSP and AMA1 respectively). A single blended 1 mL IM injection will be given using a syringe at 24 weeks.

**Reference Product and Dose Regimen**

No reference product or regimen will be used for comparison other than controls for infectivity during the sporozoite challenge.

Table of Contents

[List of Abbreviations 11](#__RefHeading___Toc225743283)

[GLOSSARY OF TERMS 13](#__RefHeading___Toc225743284)

[1 Background 14](#__RefHeading___Toc225743285)

[1.1 Medical Application and Status 14](#__RefHeading___Toc225743286)

[1.2 Military Relevance 14](#__RefHeading___Toc225743287)

[1.3 Life cycle of Malaria 14](#__RefHeading___Toc225743288)

[1.4 Study Vaccine and Administration 14](#__RefHeading___Toc225743289)

[1.5 Rationale for Vaccine Design and Antigen Selection 16](#__RefHeading___Toc225743290)

[2 Study Objectives and EnDpoints 19](#__RefHeading___Toc225743291)

[**2.1** **Primary Objectives** 19](#__RefHeading___Toc225743292)

[**2.2** **Secondary Objectives** 19](#__RefHeading___Toc225743293)

[**2.3** **Exploratory Objectives and Methods of Assessment** 19](#__RefHeading___Toc225743294)

[**2.4** **Primary Endpoints and Assessment of Endpoints** 20](#__RefHeading___Toc225743295)

[**2.5** **Secondary Endpoints** 20](#__RefHeading___Toc225743296)

[3 Study Design Overview 21](#__RefHeading___Toc225743297)

[**3.1** **Overall Study Design** 21](#__RefHeading___Toc225743298)

[**3.2** **Study Event Schedule** 22](#__RefHeading___Toc225743299)

[4 General Study Aspects 25](#__RefHeading___Toc225743300)

[4.1 Ethics and Regulatory Considerations 25](#__RefHeading___Toc225743301)

[4.2 Risk Benefit Considerations 25](#__RefHeading___Toc225743302)

[4.2.1 Benefits 25](#__RefHeading___Toc225743303)

[4.2.2 Potential Risks to Subjects and Mitigation 26](#__RefHeading___Toc225743304)

[4.2.3 Risks to Study Personnel 28](#__RefHeading___Toc225743305)

[4.2.4 Transmission of Malaria to the Community 28](#__RefHeading___Toc225743306)

[**4.3** **Measures to Minimize Bias** 29](#__RefHeading___Toc225743307)

[**4.4** **Justification of Sample Size** 29](#__RefHeading___Toc225743308)

[5 Subjects and Cohorts 30](#__RefHeading___Toc225743313)

[**5.1** **Recruitment** 30](#__RefHeading___Toc225743314)

[**5.2** **Informed Consent** 30](#__RefHeading___Toc225743315)

[**5.3** **Screening** 30](#__RefHeading___Toc225743316)

[**5.4** **Inclusion Criteria** 32](#__RefHeading___Toc225743317)

[**5.5** **Exclusion Criteria** 32](#__RefHeading___Toc225743318)

[**5.6** **Elimination Criteria** 33](#__RefHeading___Toc225743319)

[**5.7** **Subject Completion** 34](#__RefHeading___Toc225743320)

[**5.8** **Subject Dropout** 34](#__RefHeading___Toc225743321)

[6 Conduct of the Study 35](#__RefHeading___Toc225743322)

[6.1 Investigational Product Preparation, Storage, and Disposition 36](#__RefHeading___Toc225743323)

[**6.2** **Infecting Mosquitoes with *P. falciparum* Parasites** 37](#__RefHeading___Toc225743324)

[**6.3** **Detailed Description of Study Phases and Visits** 37](#__RefHeading___Toc225743325)

[**6.4** **The Day of the Challenge** 47](#__RefHeading___Toc225743326)

[**6.5** **Determination of Parasitemia** 47](#__RefHeading___Toc225743327)

[**6.6** **Management of Subjects Post-challenge** 48](#__RefHeading___Toc225743328)

[**6.7** **Concomitant Medications and Treatment** 49](#__RefHeading___Toc225743329)

[**6.8** **Sample and Data Handling** 50](#__RefHeading___Toc225743330)

[**6.9** **Holding Rules** 50](#__RefHeading___Toc225743331)

[**6.10** **Stopping Rules** 50](#__RefHeading___Toc225743332)

[7 Additional Safety Considerations 51](#__RefHeading___Toc225743333)

[8 Adverse Events 51](#__RefHeading___Toc225743334)

[**8.1** Solicited and Unsolicited Adverse Events 51](#__RefHeading___Toc225743335)

[**8.1.1** Adverse Event Definition 51](#__RefHeading___Toc225743336)

[**8.1.2** Surveillance Period for Occurrence of Adverse Events 52](#__RefHeading___Toc225743337)

[**8.1.3** Recording and Reporting adverse events 52](#__RefHeading___Toc225743338)

[**8.1.4** Solicited Adverse Events 53](#__RefHeading___Toc225743339)

[**8.1.5** Unsolicited Adverse Events 54](#__RefHeading___Toc225743340)

[**8.1.6** Assessment of Intensity 54](#__RefHeading___Toc225743341)

[**8.1.7** Assessment of Causality 57](#__RefHeading___Toc225743342)

[8.1.8 Adverse Event Follow-up 58](#__RefHeading___Toc225743343)

[**8.2** **Serious and Unexpected Adverse Events** 58](#__RefHeading___Toc225743344)

[**8.2.1** Definition of a Serious Adverse Event 58](#__RefHeading___Toc225743345)

[**8.2.2** Surveillance Period for Occurrence of Serious Adverse Events 59](#__RefHeading___Toc225743346)

[**8.2.3** Reporting a Serious or Unexpected Adverse Event 59](#__RefHeading___Toc225743347)

[**8.3** **Pregnancy** 61](#__RefHeading___Toc225743365)

[**8.4** **Treatment of Adverse Events** 61](#__RefHeading___Toc225743366)

[9 Data Evaluation 62](#__RefHeading___Toc225743367)

[**9.1** **Data Inclusion Criteria** 62](#__RefHeading___Toc225743368)

[9.1.1 Cohort for analysis of safety 62](#__RefHeading___Toc225743369)

[9.1.2 Cohort for analysis of immunogenicity 62](#__RefHeading___Toc225743370)

[**9.2** **Analysis of Demographics** 62](#__RefHeading___Toc225743371)

[**9.3** **Analysis of Primary Endpoints** 62](#__RefHeading___Toc225743372)

[**9.3.1** Analysis of Safety and Tolerability 62](#__RefHeading___Toc225743373)

[**9.3.2** Analysis of Efficacy 63](#__RefHeading___Toc225743374)

[**9.4** **Analysis of Secondary Endpoints** 63](#__RefHeading___Toc225743375)

[**9.4.1** Analysis of Immunogenicity 63](#__RefHeading___Toc225743376)

[**9.4.2** Subgroup Analysis 65](#__RefHeading___Toc225743377)

[**9.5** **Final Report** 66](#__RefHeading___Toc225743378)

[**9.6** **Future Plans** 66](#__RefHeading___Toc225743379)

[10 Administrative Matters 66](#__RefHeading___Toc225743380)

[**10.1** **Publication Policy** 66](#__RefHeading___Toc225743381)

[11 References 66](#__RefHeading___Toc225743382)

Appendix 1: INFORMED CONSENT FOR IMMUNIZED SUBJECTS

**APPENDIX 2: INFORMED CONSENT FOR non-immunized SUBJECTS**

Tables

*Table 1a: Study Event Schedule: Timeline and Procedures through Day 210…………………….………………….21*

*Table 1b: Study Event Schedule: Timeline and Procedures from day of challenge through end of the study……22*

*Table 2: Post challenge malaria symptoms……………………………….…………………..…………….…………….49*

*Table 3a: Solicited local and general adverse events…………………….…………………..…………….……………55*

*Table 3b: Solicited cardiac adverse events post challenge.…………….…………………..…………….…………….55*

*Table 4: Intensity grading of LOCAL solicited adverse events…………………………………………….................56*

*Table 5: Intensity grading of Fever and SYSTEMIC solicited adverse events…………………………………..56-57*

*Table 6: Intensity grading of other adverse events………………………………………………………………………57*

*Table 7: Serum biochemistry lab value grading…………………………………………………………………………57*

*Table 8: Hematology lab value grading……………………………………………………………………..............57-58*

*Table 9: Relationship between the adverse event and the study event for causality assessment………………….58*

**Attachments**

ATTACHMENT A: PRINCIPAL INVESTIGATOR SIGNATURE PAGE FOR IRB OF RECORD

ATTACHMENT B: ADMINISTRATIVE MATTERS

ATTACHMENT C: OVERVIEW OF THE RECRUITMENT PLAN

ATTACHMENT D: LIST OF STUDY SPECIFIC PROCEDURES

ATTACHMENT E: CONSENT FOR HIV ANTIBODY BLOOD TEST

ATTACHMENT F: BLOOD DONATION CONSENT FORM

ATTACHMENT G: Informed Consent Comprehension Assessment

ATTACHMENT H: SUPERVISOR APPROVAL FOR ACTIVE MILITARY PERSONNEL

ATTACHMENT I: LIST OF INVESTIGATORS AND THEIR ROLES AND RESPONSIBILITIES

ATTACHMENT J: CONSENT TO USE STUDY SUBJECT PHOTOS

ATTACHMENT K: SAFETY AND IMMUNOGENICITY DATA FOR THE ongoing usmmvp ad5 vaccine trial with the identical ad5 vaccine

ATTACHMENT L: Gaziano study for assessment of cardiac risk factor by non-laboratory methods

ATTACHMENT M: RECRUITMENT BRIEFING

ATTACHMENT N: RECRUITMENT FLYER 1

ATTACHMENT O: RECRUITMENT FLYER 2

ATTACHMENT P: CASE REPORT FORMS

attachment q: subject registry database sheet

attachment R: emergency notification card

ATTACHMENT S: temperature log

**List of Abbreviations**

| AE | Adverse event | HRPO | Human Research Protection Office |
| --- | --- | --- | --- |
| AFRIMS | Armed Forces Research Institute of Medical Sciences | HSRRB | Human Subjects Research Review Board |
| Ag | Antigen | IAW | In accordance with |
| AI | Associate investigator | ICH | International Conference on Harmonization |
| ALT | Alanine aminotransferase | ICS | Intra-cytokine staining |
| AMA1 | Apical membrane antigen 1 | IFA | Indirect fluorescent antibody |
| AST | Aspartate aminotransferase | IFN-γ | Interferon gamma |
| AR | Army Regulation | IRB | Institutional Review Board |
| β-HCG | Beta-Human Choriogonadotropin | MIDRP | Military Infectious Disease Research Program |
| BUMED | Navy Bureau of Medicine and Surgery | NNMC | National Naval Medical Center |
| BUN | Blood Urea Nitrogen | NMRC | Naval Medical Research Center |
| CBC | Complete Blood Count | O(H)RP | Office for (Human) Research Protections |
| CI | Confidence Interval | PU | Particle Units |
| CMI | Cell Mediated Immunity | PBMC | Peripheral Blood Mononuclear Cells |
| CRF | Case Report Form | *Pf, P. falciparum* | *Plasmodium falciparum* |
| CSP | Circumsporozoite protein | PI | Principal investigator |
| DoD | Department of Defense | *Pv, P. falciparum* | Plasmodium *falciparum* |
| DHSP | Division of Human Subject Protection | qRT-PCR | Quantitative Real-Time Polymerase Chain Reaction |
| ELISA | Enzyme linked immunosorbent assay | SAE | Serious adverse event |
| ELISpot | Enzyme linked immunospot assay | SECNAV | Secretary of the Navy |
| GCP | Good Clinical Practices | SOP | Standard Operating Procedure |
| GIA | Growth Inhibition Assay | USAMRMC | United States Army Medical Research and Materiel Command |
| HBsAg | Hepatitis B surface antigen | WRAIR | Walter Reed Army Institute of Research |
| HIV | human immunodeficiency virus | WRAMC | Walter Reed Army Medical Center |
| HLA | Human Leukocyte Antigen | μL | Microliter |

**GLOSSARY OF TERMS**

| **Subject(s)** | Term used throughout the protocol to denote the enrolled individual(s) to be challenged unless specified. Excludes blood donors who are covered by a separate human use protocol under execution at AFRIMS. |
| --- | --- |
| **Medical Monitor** | A qualified medical doctor able to fulfill the unique requirements and responsibilities of a Department of Defense (DoD) medical monitor as outlined in section 4.4.3 of DoD Directive 3216.02, March 25, 2002: Protection of Human Subjects and Adherence to Ethical Standards in DoD-Supported Research. In brief, the DoD requires an independent medical monitor for greater than minimal risk research [as defined in 32 CFR 219.102(i), reference (c)]. Medical monitors serve as the subject advocates. |
| **Study Monitor** | An individual who is responsible for assuring proper conduct of a clinical study. |
| **Eligible** | Subjects who have signed informed consent, screened and found to be qualified for enrollment into the study based upon strict adherence to inclusion/exclusion criteria. |
| **Enroll** | To officially assign eligible subjects who undergo the first study-specific procedure to a study cohort |
| **Protocol amendment** | Any change in a clinical protocol that affects the safety of subjects, the scope, design, assessments or scientific validity of the clinical investigation, e.g., dose change, duration of treatment, number of subjects, control group(s), the assessments. |
| **Study Specific Procedures (SSP)/Work Instructions (WI)** | Specific written guidance for execution of procedures unique to this study and not covered by institutional SOPs. SSPs/WIs should be written and approved by the PI before study implementation, reviewed by the Quality Assurance Office at the local institution and filed with the study regulatory documents. Copies should be provided to the sponsor upon request. |

# Background

## Medical Application and Status

Malaria is a major public health problem worldwide. There are an estimated 300-500 million clinical cases annually which cause between one and three million deaths [1], so a vaccine to prevent this disease or lessen the severity of the illness would be widely beneficial to both military and civilian populations. Increasing drug resistance of the parasite and insecticide resistance of the vector further emphasizes the importance of developing an effective malaria vaccine which will circumvent the various forms of resistance.

## Military Relevance

A soldier or sailor with malaria can be incapacitated from one to three weeks, and a *P. falciparum* infection can rapidly become life threatening if not promptly diagnosed and treated. In addition, military personnel can be exposed to more than one malaria species in today’s complex military operation. The mission-altering evacuation of 32 U.S. Marines from Liberia in 2003 exemplifies the impact malaria can have even on present day military operations and personnel [2].

## Life cycle of Malaria

Natural transmission of malaria occurs through exposure of the host to the bite of an infective female *Anopheles* mosquito. *Plasmodium* sporozoites in the salivary glands are inoculated into the peripheral circulation of the host and travel through the bloodstream to invade hepatocytes (usually within 30 minutes) in the liver. Within the hepatocyte, sporozoites undergo amplification for five to ten days, each producing as many as 30,000 merozoites per infected hepatocyte [3]. There are no clinical symptoms of malaria during the liver-stage infection. Hepatic merozoites are then released into the bloodstream, and each parasite invades an erythrocyte initiating a cycle of asexual stage amplification. This stage of infection is responsible for the clinical symptoms of malaria. Erythrocytes containing mature schizonts of *P. falciparum* rupture 48 hours after erythrocyte invasion, releasing 15 to 30 merozoite progeny, which bind to and enter uninfected erythrocytes to begin a new cycle. This process takes about 2 days following release from the liver. Alternatively, some merozoites differentiate into male or female gametocytes which are long-lived and motile. When the gametocytes are ingested by a biting female mosquito they become activated and continue the sexual phase of the parasite life cycle within the mosquitoes. Sporozoites are produced then migrate to the mosquito’s salivary glands and await inoculation into a human host.

## Study Vaccine and Administration

- - 1. Description of the Study Vaccine

The study vaccine, which will be studied under an IND allowance provided by the FDA (BB-IND 13977), contains two components: a DNA priming component and an adenovirus-vectored boosting component termed DNA and Ad respectively. Combined they form the DNA-Ad vaccine.

DNA vaccine, manufactured by Vical Inc (San Diego) is a combination of two recombinant plasmids encoding 2 *Plasmodium falciparum* (*Pf*) antigens, CSP (circumsporozoite protein) and AMA1 (apical membrane antigen 1). The plasmid encoding *Pf*CSP contains a full-length synthetic *Pf*CSP gene except for a sequence coding 64 amino acids which is about 1/3 of the repeat region, and is codon-optimized for optimal expression in human cells. The plasmid encoding *Pf*AMA1 contains a synthetic *Pf*AMA1 gene, also codon-optimized with missing genes coding for the transmembrane and intracellular C-terminal domains of the *Pf*AMA1 protein as well as removal of native signal sequence from the N-terminus. This method of not synthesizing hydrophobic regions is a common practice in making recombinant proteins and has not been associated with reduced immunogenicity. This plasmid contains a human tissue plasminogen activator (TPA) signal sequence, the *Pf*CSP and *Pf*AMA1 genes are cloned in-frame with the TPA signal sequence which helps *Pf* protein to be expressed efficiently. Since the PfAMA1 construct expressed by the DNA vector does not contain a signal sequence, the TPA signal sequence was added to replace the native signal sequence in the *Pf*AMA1 plasmid and is upstream of the native signal sequence in the *Pf*CSP plasmid, a circumstance that facilitates expression and trafficking of the protein within the cell.

The DNA vaccine is vialed as a mixture of plasmids and phosphate-buffered saline (PBS). Each vial contains 1 mg of each plasmid (2 mg total), and the vaccine is administered as two injections; one into the deltoid muscle of each arm by jet injection. Each injection is 1 mL total containing 0.5 mg of each of two plasmids. Each of the DNA vaccine vials is labeled for human administration and includes the following statement: “New Drug - Limited by Federal or United States Law to Investigational Use.”

The Ad vaccine, manufactured by GenVec Inc., contains two replication-deficient adenovirus vectors expressing *Pf* CSP and *Pf* AMA1. The GV11 adenovirus backbone was chosen due to its high level transgene capacity (7-8 kb, much larger than the size of our malaria gene inserts which are less than 3 kb including promoter) and multiple areas of deletions in the E1, E3, and E4 regions, which significantly reduces the risk of replication-competent adenovirus resulting from recombination events or via mutation during manufacturing. The *Pf* genes (codon-optimized CSP and AMA1 genes from 3D7 strain) in the two vectors are inserted into the E1 region (selected for higher level of antigen expression) with transcription initiated by a human cytomegalovirus (CMV) immediate-early promoter and terminated by a SV40 polyadenylation sequence, which are used in various eukaryotic expression systems and well-known for resulting in high levels of antigen expressions. The CSP gene in the adenovector is the same as the CSP gene in the plasmid except that since there is a native signal sequence; no TPA signal sequence is added. The AMA1 gene in the adenovector is the same as the AMA1 gene in the plasmid except that it encodes the full length AMA1 protein, including the N-terminal native signal sequence, the transmembrane domain, and the C-terminal intracellular domain, and it contains a native signal sequence (hence the lack of need for TPA signal sequence).

The two adenovectored antigens, NMRC-MV-Ad-*Pf*C and NMRC-MV-Ad-*Pf*A, are vialed separately in buffered saline called final formulation buffer (FFB) and then mixed into a third vial prior to administration. This vaccine is also given intramuscularly; 1 x 10^10^ *pu* of each adenovectored antigen (2 x 10^10^ *pu* total in 1ml) is injected by needle into the deltoid muscle. Each of the Ad vaccine vials is labeled for human administration and includes the following statement: “Caution: New Drug Limited by Federal Law to Investigational Use.”

## Rationale for Vaccine Design and Antigen Selection

- - 1. Study Rationale

The goal of this study is to evaluate if the DNA-Ad vaccine that targets both the liver and blood stages of the malaria life cycle is safe and efficacious, as a part of the goals of USMMVP to develop a vaccine to prevent infection and/or lessen the severity of disease caused by the *P. falciparum* malaria parasite. More specifically, this DNA-Ad vaccine contains a liver stage antigen (CSP) and an antigen that is present in both the liver and blood stages (AMA1) designed to prevent infection by killing the majority of developing parasites in the liver and to prevent severe disease and death should break-through blood stage infections occur.

- - 1. Rationale for CSP Antigen Selection

The CSP is the major coat protein of the *P. falciparum* sporozoite [4]. Antibody and T cell responses directed against the CSP are found in clinically immune adult residents of malaria endemic areas and also found in human subjects protected from malaria by immunization with radiation attenuated *P. falciparum* sporozoites [5-10]. A large number of clinical trials have been conducted with CSP-based vaccines using a variety of vaccine delivery systems and adjuvants. To date, the most promising CSP vaccine tested has been RTS,S, a recombinant protein based vaccine that has a protective efficacy of 22% to 56% in challenge trials [11-13] and up to 66% in field exposure trials [14-19]

- - 1. Rationale for AMA1 Antigen Selection

Several lines of evidence suggest that AMA1 is critical for parasite survival and hence, is an excellent vaccine target. AMA1 synthesis, stage-specific processing, and localization coincide with the timing of hepatocyte invasion by sporozoites and erythrocyte invasion by merozoites [20-22] which may explain activities of AMA1 for both liver and blood stages. Attempts to knock out the *P. falciparum* AMA1 gene have not yielded viable parasites, indicating that the protein plays a vital role in the parasite life cycle [23]. Clinical studies in humans have shown that AMA1, when presented as a recombinant protein in two different formulations, was safe and well tolerated ([24], [25], [26]). Malkin [24] demonstrated functional humoral immune responses based on ELISA, confocal microscopy and growth inhibition assay (GIA). Both pre-clinical and clinical evidence suggest that AMA1 would be an excellent and safe antigen candidate for malaria vaccine.

- - 1. Rationale for DNA Priming

In almost a decade of use in human clinical trials for various diseases, DNA has been shown to be safe and immunogenic [27-34] but lacking in efficacy as a sole vaccine platform against malaria [31]. DNA plasmids combined with viral vectors in a heterologous prime-boost approach have the potential to induce a more potent immune responses than either platform alone [33, 34, 36, 37] and may present a way of bypassing the effects of pre-existing immunity to the viral vectors. Also the existence of pre-existing neutralizing antibodies to Ad5, whether present because of wild type exposure or possibly from an Ad5 vaccine, poses a potential concern for the use of Ad5 alone as a vaccine platform due to the possibility of vaccine neutralization. DNA priming offers the possibility of bypassing Ad5 neutralizing antibodies either by allowing for only one dose of Ad5 in a multi-dose regimen so that no Ad5 neutralizing antibodies are generated by the prime or by enhancing the immune response to a point that it can overcome the effects of pre-existing antibodies on the Ad5 boost. Genetically-based vaccines such as DNA plasmids and viral vectors induce high levels of CD4+ and CD 8+ T cells, which are believed capable of mediating sterile protection following exposure to malaria [30, 35, 38].

- - 1. Rationale for Recombinant Adenovirus Serotype 5

Studies in animal models and human trials of malaria [39-45] have established that recombinant adenovirus vectors are potent inducers of antigen-specific immune responses and protective immunity against pathogen challenge. Data demonstrated that recombinant adenovirus vaccines administered in either homologous or heterologous immunization regimens are very effective at both priming and boosting CD8+ and CD4+ T cell and antibody responses and can confer sterile protection against parasite challenge (unpublished NMRC data). An ongoing USMMVP study, the current Ad clinical trial, is a two-part, Phase 1/2a, open-label, dose-escalating trial of the Ad vaccine administered intramuscularly in healthy malaria-naïve adult subjects, involving a dose-escalation part (part A) and a challenge part (part B). The initial immunogenicity data from the ongoing Ad-alone trial at USMMVP at day 10 and day 28 post immunization have shown some of the highest ELISpot responses ever recorded following immunization with the CSP and AMA1 antigens using any platform (Sedegah, presented at the Malaria Vaccine for the World, September 2007, London, UK). Safety results obtained indicate that both the 2 x 10^10^ *pu* and 1 x 10^11^ *pu* doses are safe, well-tolerated, and immunogenic in individuals who were not previously exposed to Ad5, and 2 x 10^10^ *pu* is also safe, well-tolerated, and immunogenic after two doses were given 16 weeks apart to individuals who were both exposed or not exposed to Ad5 previously. For more details on the latest safety and immunogenicity results of the currently ongoing USMMVP Ad5 vaccine trial, please refer to Attachment K.

- - 1. Rationale for Dosing Regimen and Intervals

Recent studies have defined two subsets of memory T cells, effector memory cells and central memory cells, the timing of the maturation of these subsets may influence development of protective immunity [47-50]; central memory cells may be better mediators of long term protective immunity [50]. An increased interval between immunizations may allow time for effector memory cells to differentiate into central memory cells and thereby improve the amnestic response and enhance vaccine efficacy and duration of protection. Studies using the *P. yoelii* murine model have shown that increasing the interval between immunizations resulted in enhancement in the magnitude and frequency of antigen-specific CD8+ T cell IFN-γ responses and TNF-α responses, antigen-specific CD4+ T cell IFN-γ and IL-2 responses, antigen-specific and parasite-specific antibody responses, and protective efficacy against sporozoite challenge (Doolan, unpublished data). This finding is supported by the *P. knowlesi* rhesus model, involving DNA priming and recombinant poxvirus boosting, in which the parasitemia levels in the six-month interval group, but not the one-month interval group, were significantly different from the control group (p<0.05). Based on these studies, we have chosen a four month interval between priming and boosting immunizations in the clinical trial.

- - 1. Rationale for Dose Selection and Number of Doses

Immunologic data from a human clinical trial was reported by Wang et al [35] using a single antigen plasmid encoding CSP revealed that peptide-specific, genetically restricted, and CD8+ T cell-dependent CTL responses were induced by as little as two 20 μg doses of DNA; however, immunization with either 500 or 2500 μg of DNA induced significantly better CTL response and in higher numbers of subjects than lower doses. There was no significant difference between 500 and 2500 μg dosages overall. This study also revealed that CTL responses, as measured by chromium release assays two weeks after the third immunization, were significantly greater after the second immunization (p=0.026). This study supported a desirable range of DNA dosage between 500 μg (or 0.5 mg) and 2500 μg (or 2.5 mg) per plasmid with three doses of DNA. Based on these data and the formulation constraints of this DNA vaccine, 1 mg per plasmid (1 mg for CSP and 1 mg for AMA1) for a combined vaccine dose of 2 mg administered on each of three immunization days for a total of 6 mg of DNA per subject.

- - 1. Rationale for Dosage of Adenovirus Vaccine Boost

Since part of the current Ad clinical trial, demonstrated that the higher dose did not yield superior cellular or humoral immune response, the lower total dose of 2 x 10^10^ *pu* for both antigens was recommended by the Safety Monitoring Committee to be used for part B. Subjects in part B have received two doses of Ad5 vaccines given 16 weeks apart and the safety data collected up to day 7 after the second dose continues to support the conclusion that the vaccine is safe and well-tolerated.

1. **Study Objectives and EnDpoints**
   1. **Primary Objectives**

- Assess the safety of a heterologous prime-boost vaccine regimen (DNA-Ad) consisting of a DNA vaccine prime (NMRC-M3V-D-*Pf*CA) and adenovirus type 5 vaccine boost (NMRC-M3V-Ad-*Pf*CA) in healthy malaria-naïve adults
  1. **Secondary Objectives**

1. Assess the protective efficacy against sporozoite challenge with Plasmodium falciparum (*Pf*), 3D7,

2. Assess the humoral immunogenicity of this prime-boost regimen to CSP and AMA1 antigens by enzyme linked immunosorbent assay (ELISA)

3. Assess the cellular immunogenicity of this prime-boost regimen to CSP and AMA1 antigens by enzyme linked immunospot assay (ELISpot) and intracellular staining (ICS)

- 1. **Exploratory Objectives and Methods of Assessment**

1. Assess evidence of vaccine effect on prepatent period as determined by PCR and thick smear collected at closely spaced intervals at least daily from day 6 to the last of the three consecutive negative smears.
2. Assess evidence of vaccine effect on rate of development of blood stage parasites as determined by PCR and thick blood smears collected at closely spaced intervals at least daily from day 6 post challenge to the last of the three consecutive negative smears.
3. Measure host immune response by using oligonucleotide microarrays to assess breadth and magnitude of immune response
4. Measure the functional humoral immune response by Growth Inhibition Assay (GIA)
5. Analysis of immunofluorescence assay (IFA) titers against sporozoites and blood stage parasites
6. To determine if a specific antibody or cellular response, or gene expression profile (as assessed by microarray analysis) correlates with protection. Determination of correlates of protection are made by analyzing 1) antibody titers, 2) ELISpot responses, 3) gene expression profile after challenge to observe if there are parameters with a positive association with protection, and the extent of such associations.
7. Compare the safety, immunogenicity, and protective efficacy between groups with and without pre-existing neutralizing antibodies against adenovirus type 5 (Ad5+/Ad5-)
8. Assess the Ad5 serology kinetics in both Ad5+ and Ad5- subjects following immunization with Ad
   1. **Primary Endpoints and Assessment of Endpoints**

The vaccine will be considered safe and well-tolerated if there are no severe or serious adverse events (AE) related to vaccine administration or if any severe events are relatively benign (e.g. erythema meeting criteria for severe due to its dimensions but not significantly affecting the activities of daily living for the subject) or brief in duration (e.g. less than 48 hours). The AEs will be assessed according to the method below.

a. Occurrence, severity, and duration of any solicited symptoms starting on the day of

immunization through day 7 after each immunization

b. Occurrence of any unsolicited symptoms, abnormal physical findings, and laboratory

values starting from the day of immunization through day 28 after each immunization

c. Occurrence of any serious adverse events, as defined in 21 CFR 312.32, during the five-year study period

- 1. **Secondary Endpoints**
     - 1. The DNA-Ad vaccine is considered efficacious if it offers any degree of sterile or partial protection that reaches statistical significance (p<0.05). The vaccine efficacy will be determined by two parameters: 1) sterile protection for 28 days after challenge, 2) partial protection as determined by delay to parasitemia by smears and PCR. More specifically, the degree of sterile protection will be assessed by comparing the number of immunized subjects who did not develop malaria infection after 28 days of challenge and the control subjects over the same time period. The presence of partial protection will be determined by observance of statistical difference between the timing of the onset of parasitemia between immunized and control subjects.
       2. The DNA-Ad vaccine is considered to confer humoral immunity if the increase of antibody response for CSP or AMA1 by ELISA post immunization is statistically significant (p<0.05) compared to the levels before immunization.
       3. The DNA-Ad vaccine is considered to confer cellular immunity if a positive ELISpot response is detected after immunization which will have the following 3 characteristics: (1) a statistically significant difference between the number of spot forming cells in triplicate test wells and triplicate control wells; (2) at least a doubling of spot forming cells in test wells relative to control wells; and (3) a difference of at least 5 spots between test and control wells.

1. **Study Design Overview**
   1. **Overall Study Design**

This is a Phase 1/2a, open-label, multi-centered controlled trial that includes one study cohort and one infectivity control cohort. The control cohort consists of six healthy non-immunized subjects who will undergo a sporozoite challenge. The study cohort will consist up to 20 (no fewer than 16) healthy subjects on the day of the first DNA immunization with or without serologic evidence of prior adenovirus serotype 5 infections. They will receive three doses of DNA, given at intervals of 4 weeks, and one dose of Ad given at 24 weeks. Subjects will be seen at either the WRAIR Clinical Trials Center after each immunization for evaluation and will be questioned on the occurrence, severity, and duration of solicited symptoms.

After the study cohort receives all four immunizations, both cohorts will undergo sporozoite challenge. *Anopheles stephensi* mosquitoes infected with *P. falciparum* will be allowed to feed on the forearms of the subjects in the WRAIR insectary. Subjects will be observed following the challenge and will be required to stay overnight at a hotel from day 10 to day 19 where the subjects will be closely monitored post-infection (thick blood films will be made beginning on day 6 after challenge) and treated appropriately as soon as infection becomes patent in the peripheral blood. The follow-up visits will occur in two stages, the active stage involves in-person visits which begins after the first immunization until 12 weeks after the challenge (approximately 305 days after the first immunization); the long-term stage involves contact between subjects and CTC by telephone, email, or mail on an annual basis beginning after the last in-person visit until 5 years after the first immunization. Study schedules for the active stage are provided in section 3.2.

- 1. **Study Event Schedule**

**Table 1a. Study timeline and procedures through day 210**

| **Immunization** | **Screen-**  **ing^a^** | **DNA # 1** |  | | | | **DNA # 2** |  | | | | **DNA # 3** |  | | | | | | **Ad** |  | | | |
| --- | --- | --- | --- | --- | --- | --- | --- | --- | --- | --- | --- | --- | --- | --- | --- | --- | --- | --- | --- | --- | --- | --- | --- |
| **Study Day** | -90 to  -3 | 0 | 1 | 2 | 7 | 14 | 28 | 29 | 30 | 35 | 42 | 56 | 57 | 58 | 63 | 70 | 84 | 126 | 168 | 169 | 170 | 175 | 182 |
| **Study Day relative to each vaccine** |  | 0 | 1 | 2 | 7 | 14 | 0 | 1 | 2 | 7 | 14 | 0 | 1 | 2 | 7 | 14 | 28 | 70 | 0 | 1 | 2 | 7 | 14 |
| **Study Visit #** | 1 | 2 | 3 | 4 | 5 | 6 | 7 | 8 | 9 | 10 | 11 | 12 | 13 | 14 | 15 | 16 | 17 | 18 | 19 | 20 | 21 | 22 | 23 |
| **Informed Consent** | ● |  |  |  |  |  |  |  |  |  |  |  |  |  |  |  |  |  |  |  |  |  |  |
| **Review criteria & contraindications** | ● | ● | ● | ● | ● | ● | ● | ● | ● | ● | ● | ● | ● | ● | ● | ● | ● | ● | ● | ● | ● | ● | ● |
| **Review of history & medications** | ● | ● | ● | ● | ● | ● | ● | ● | ● | ● | ● | ● | ● | ● | ● | ● | ● | ● | ● | ● | ● | ● | ● |
| **Review & recording of AE/SAE** |  | ● | ● | ● | ● | ● | ● | ● | ● | ● | ● | ● | ● | ● | ● | ● | ● | ● | ● | ● | ● | ● | ● |
| **Vital signs: BP, pulse & temp** | ● | ● | ● | ● | ● | ● | ● | ● | ● | ● | ● | ● | ● | ● | ● | ● | ● | ● | ● | ● | ● | ● | ● |
| **Physical examination^b^** | ● | ● | ● | ● | ● | ● | ● | ● | ● | ● | ● | ● | ● | ● | ● | ● | ● | ● | ● | ● | ● | ● | ● |
| **β- HCG –urine (women only)^c^** | ● | ● |  |  |  |  | ● |  |  |  |  | ● |  |  |  |  |  |  | ● |  |  |  |  |
| **Screening labs^d^ (14 mL) + EKG** | ● |  |  |  |  |  |  |  |  |  |  |  |  |  |  |  |  |  |  |  |  |  |  |
| **Safety labs (7 mL)** |  | ● |  |  | ● |  | ● |  |  | ● |  | ● |  |  | ● |  | ● | ● | ● |  |  | ● |  |
| **Humoral immunity (ELISA, IFA, GIA) (10ml)** |  | ● |  |  |  | ● | ● |  |  |  | ● | ● |  |  |  | ● | ● |  | ● |  |  |  |  |
| **Ad 5 titer (5 mL)** |  | ● |  |  |  |  |  |  |  |  |  |  |  |  |  |  |  |  | ● |  |  |  | ● |
| **Cellular immunity (ELISpot, ICS) (125 mL)** |  | ● |  |  |  |  |  |  |  |  |  |  |  |  |  |  | ● |  | ● |  |  |  |  |
| **Microarray (8 mL)** |  | ● |  |  |  |  |  |  |  |  |  | ● | ● | ● |  |  |  |  | ● |  | ● |  |  |
| **Blood draw volume per visit (mL)** | 14 | 155^d^ | 0 | 0 | 7 | 10 | 17 | 0 | 0 | 7 | 10 | 25 | 8 | 8 | 7 | 10 | 142 | 7 | 160 | 0 | 8 | 7 | 10 |
| **Cumulative blood volume (mL)** | 14 | 214 |  |  | 221 | 231 | 248 |  |  | 255 | 265 | 290 | 298 | 306 | 313 | 323 | 465 | 472 | 632 |  | 640 | 647 | 657 |
| **Compensation** | 25 | 100 | 50 | 50 | 100 | 100 | 100 | 50 | 50 | 100 | 100 | 100 | 100 | 100 | 100 | 100 | 100 | 100 | 100 | 50 | 100 | 100 | 100 |

^a^ Some or all of the Study Dates in this section represent a window of dates in which all the subjects must be seen. Screening visits apply to both immunized and control subjects.

^b^Exam will occur at indicated visits, as well as any visit where it is felt to be indicated by an investigator.

^c^Pregnancy test on the day of each immunization and challenge. Repeat testing may be requested by the PI and performed at unscheduled visits prior to the challenge phase to ensure subject eligibility.

^d^Screening labs: CBC with diff (3 mL), AST, ALT, Cr, Glu, total bilirubin (total 3.5ml), HIV (3.5 mL), hepatitis B and C (3.5 mL). The same screening timeline (day -90 to -3) and the same tests are done for the control subjects as well except that immunized subjects will also have Ad5 titer done. Ad5 titers (5 mL) will be drawn on day 0, before the Ad dose (the day of Ad immunization), day 14 after Ad, day of challenge, (the last 2 time points may become 1 if the challenge occurs 14 days post challenge). HLA typing will be done on day 0 for immunized volunteers and on day of challenge for control volunteers.

**Table 1b. Study timeline and procedures from the Day of Challenge to the end of the study for immunized (*imm*) and control subjects**

|  | **C**hallenge^e^ | **Post Challenge Week 1** | | | | | **Post Challenge Hotel Phase** | | | | | | | | | | **Additional Visits**^h^ | **Post Challenge Follow-up^a^** | | | **Final Visit** | |  |
| --- | --- | --- | --- | --- | --- | --- | --- | --- | --- | --- | --- | --- | --- | --- | --- | --- | --- | --- | --- | --- | --- | --- | --- |
| **Days post challenge** | 0 | 3 | 6 | 7 | 8 | 9 | 10 | 11 | 12 | 13 | 14 | 15 | 16 | 17 | 18 | 19 | 20-28 | 21 | 23 | 28 | | 84 | |
| **Study day, *imm*** | 196 | 199 | 202 | 203 | 204 | 205 | 206 | 207 | 208 | 209 | 210 | 211 | 212 | 213 | 214 | 215 | 216-224 | 217 | 219 | 224 | | 280 | |
| **Study visit #, *imm*** | 24 | 25 | 26 | 27 | 28 | 29 | 30 | 31 | 32 | 33 | 34 | 35 | 36 | 37 | 38 | 39 | 40+ | 40 | 41 | 42 | | 43 | |
| **Study visit #, *control*** | 2 | 3 | 4 | 5 | 6 | 7 | 8 | 9 | 10 | 11 | 12 | 13 | 14 | 15 | 16 | 17 | 18+ | 18 | 19 | 20 | | 21 | |
| **Criteria & contraindications check** | ● |  | ● | ● | ● | ● | ● | ● | ● | ● | ● | ● | ● | ● | ● | ● | ● | ● | ● | ● | | ● | |
| **Review of history & medications** | ● |  | ● | ● | ● | ● | ● | ● | ● | ● | ● | ● | ● | ● | ● | ● | ● | ● | ● | ● | | ● | |
| **Review and recording of AE/SAE** | ● |  | ● | ● | ● | ● | ● | ● | ● | ● | ● | ● | ● | ● | ● | ● | ● | ● | ● | ● | | ● | |
| **Vital signs: BP, pulse & temp** | ● |  | ● | ● | ● | ● | ● | ● | ● | ● | ● | ● | ● | ● | ● | ● | ● | ● | ● | ● | | ● | |
| **Physical examination ^b^** | ● |  | ● | ● | ● | ● | ● | ● | ● | ● | ● | ● | ● | ● | ● | ● | ● | ● | ● | ● | | ● | |
| **HCG –urine (women only)^c^** | ● |  |  |  |  |  |  |  |  |  |  |  |  |  |  |  |  |  |  |  | |  | |
| **Safety labs (7 mL)** | ● |  |  |  |  |  |  |  |  |  |  |  |  |  |  |  |  |  |  | ● | | ● | |
| **Malaria smear, PCR (4ml)^f^** | f |  | f | f | f | f | f | f | f | f | f | f | f | f | f | f | f |  |  |  | |  | |
| **Humoral immunity (ELISA, IFA, GIA) (10ml)** | ● |  |  |  |  |  |  |  |  |  |  |  |  |  |  |  |  |  |  | ● | | ● | |
| **Ad 5 titer (5 mL)** | ● |  |  |  |  |  |  |  |  |  |  |  |  |  |  |  |  |  |  |  | |  | |
| **Cellular immunity (ELISpot, ICS, 125 mL)** | ● |  |  |  |  |  |  |  |  |  |  |  |  |  |  |  |  |  |  | ● | | ● | |
| **Microarray (8 mL)** | ● | ● |  |  |  |  |  |  |  |  |  |  |  |  |  |  |  |  |  |  | |  | |
| **Direct Observation Therapy^g^** |  |  |  |  |  |  | g | g | g | g | g | g | g | g | g | g | g |  |  |  | |  | |
| **Sporozoite Challenge in insectary** | ● |  |  |  |  |  |  |  |  |  |  |  |  |  |  |  |  |  |  |  | |  | |
| **Blood draw volume per visit (mL)^i^** | 159^d^ | 8 | 4 | 4 | 4 | 4 | 4 | 4 | 4 | 4 | 4 | 4 | 4 | 4 | 4 | 4 | 20 | 0 | 0 | 142 | | 142 | |
| **Cumulative blood volume (mL)** | 816 | 824 | 829 | 833 | 837 | 841 | 845 | 849 | 853 | 857 | 861 | 865 | 869 | 873 | 877 | 881 | 901 |  |  | 1023 | | 1165 | |
| **Compensation ($)^j^** | 250 | 100 | 100 | 100 | 100 | 100 | 200 | 200 | 200 | 200 | 200 | 200 | 200 | 200 | 200 | 200 | 100 | 50 | 50 | 100 | | Up to 250 | |

^e^The day of challenge could be between 2 to 4 weeks after the Ad dose; 4 week is assumed here. The labs indicated in table 1b are for both immunized and control volunteers.

^f^Only PCR will be collected on day of challenge (2ml). Smear and PCR will be collected between one to three times daily starting from day 6 through day 28 or until 3 days after subject becomes parasitemic.

^g^Direct Observation Therapy: will conduct DOT for all doses of chloroquine unless determined by investigators otherwise.

^h^Additional visits will held every other day (excluding holiday and weekend) between study days 20 and 28 for subjects who remain negative for malaria during the entirety of the hotel phase. After day 28, if these individuals remain asymptomatic they will resume and complete the remainder of the follow-up visits as scheduled. For the scenario in which all additional visits are required the total blood collection between study days 20 and 28 will be 16 mL, and the overall blood collection for such subjects during the study will be 1185 mL rather than the plotted 1165 mL.

^i^For the sake of calculation of blood draw amount, it is assumed that the token subject becomes parasitemic on day 14, has positive smear for 2 more days (daily smear until 3 negative smears are noted).

^j^Maximum amount of compensation is assumed here for day 10 to day 19 post challenge for twice-daily blood draw until volunteer develops parasitemia then blood draw becomes daily

1. **General Study Aspects**

This trial will take place at the WRAIR Clinical Trial Center (CTC), Silver Spring, Maryland. The study will enroll up to 20 subjects in the immunized group (no fewer than 16) on the day of the first DNA immunization, six subjects in the control group, with three alternates in each group. Recruiting will take place at the WRAIR CTC and will be done according to applicable U.S. Army regulations (i.e. AR 70-25 and AR 40-38) where applicable. The challenge will be conducted at the WRAIR insectary. Additional study sites may be added as necessary. Alternate volunteers will be enrolled on either the day of first DNA immunization or challenge (as appropriate); they cannot be enrolled at other time points.

The first dose of the DNA and the Ad vaccines will be given in a staggered fashion as a safety precaution. Two volunteers will receive the vaccine first; the rest will receive the vaccine no earlier than 3 hours after the initial two volunteers or in the next day.

Compensation: In general, compensation for completing the screening process will be $25 for all subjects. Compensation for study visits involving blood draw will be $100 per visit and $50 per visit for visits without blood draw for civilian subjects. For active duty subjects, compensation is limited to $50 and can only be given for visits in which blood is drawn, unless the subject is on approved leave, or the visit occurs outside of normal duty hours. In these cases, the active duty subject will be eligible for the same compensation as civilian subjects. Individuals can also receive compensation for recruitment of additional subjects, and they should direct potential persons interested in the study to WRAIR recruiters. Individuals will receive $25 for each recruitee that then attends a screening session and meets all inclusion and exclusion criteria. The compensation is independent of the recruited subject’s decision to enroll, but the ultimate decision for dispensation of recruiting compensation lies with the investigator or the director of the Clinical Trials Center. This study will be funded by United States Agency for International Development (USAID).

## Ethics and Regulatory Considerations

The study will be conducted according to the protocol and in compliance with Good Clinical Practices (GCP), Belmont Principles, and other applicable regulatory and Department of Defense (DoD) requirements. All identified study personnel will be trained to perform their roles and will carry out their responsibilities in accordance with the International Conference on Harmonization (ICH)/GCP guidelines. Roles and responsibilities of the study staff are presented in Attachment I.

## Risk Benefit Considerations

### *4.2.1 Benefits*

Subjects could receive an indirect benefit given that they will be screened for human immunodeficiency virus (HIV), hepatitis B virus (HBV), hepatitis C virus (HCV), EKG and cardiac risk factor screening, and will also receive a general medical check-up as part of the screening. If an abnormality is found on screening, including HIV, they will be referred to their primary physician or other health care provider where they can receive counseling and further medical attention earlier than if they did not know of their disease status. There could also be monumental benefit to humanity if a successful malaria vaccine is developed.

### *4.2.2 Potential Risks to Subjects and Mitigation*

Risks associated with Immunization

1. DNA Vaccine

For the DNA vaccine the most relevant prior clinical experience derives from another Vical-manufactured plasmid encoding the identical CSP protein (identical amino acid sequence) called the *Pf*CSP DNA vaccine. The transgene is identical except it is not optimized for mammalian expression; the backbone of *Pf*CSP DNA is likewise nearly identical to that in the DNA vaccine. The *Pf*CSP DNA vaccine (native sequence) has been tested in four clinical trials in which 75 research subjects received the vaccine [29, 30, 34-36, 49]. All four studies confirm that the DNA vaccine is safe and well-tolerated, no severe or a serious adverse event was noted by any of these studies; the majority of the AEs are local reactions and lasting less than 48 hours.

2. Ad5 Vaccine

To date, in our current trial of Ad alone (NMRC.2006.0001 - HSRRB: A-13453 - NNMC: B06-099) a total of 27 subjects have received intramuscular immunization with the Ad vaccine either at a dose of 2 x 10^10^ pu (21 of 27, six received one dose, 15 received two doses 16 weeks apart), or the higher dose of 1 x 10^11^ pu (6 of 27). The vaccine is found to be safe and well tolerated with few vaccine-related Grade 3 (severe) and no vaccine-related Grade 4 (serious) adverse events. The most frequent adverse reactions include tenderness and pain at the site of injection, and systemic symptoms such as headache, myalgia, nausea, malaise, fever, and chills. Both the local as well as the systemic reaction were mostly mild. One subject in the high dose group experienced a Grade 3 decrease in her absolute neutrophil count (ANC=936) and a Grade 1 decrease in WBC on day 2, both normalized by day 8. Further detailed study of the ANC and WBC trend in part B of the study indicated also a transient phenomenon of lowering ANC and WBC, which is compatible with expected immune response to vaccines. Due to lack of superior immunogenicity results in the higher dose group, only the lower dose (total 2 x 10^10^ particle units (*pu*), 1 x 10^10^ *pu* per each of two constructs) was selected for the regimen comparison phase of that trial, which underlies the choice of the lower dose of the Ad5 immunization for this DNA-Ad trial as well. Even after two doses have been given to subjects at the lower dose, no reduction of ANC has been observed. Therefore, along with the data above on the DNA vaccine, we anticipate that the prime-boost DNA-Ad vaccine will be safe and well-tolerated.

Risks associated with Phlebotomy

- Mild discomfort and pain
- Bruising at the venipuncture sites may occur, which usually disappears within seven days.
- Rarely, local infection can occur. Subjects will be counseled to return to the enrollment site if infection or any other unexpected outcome is suspected.
- Phlebotomy will be done by trained staff using aseptic technique, to reduce the risks of complications.
- Accumulative blood loss.
- The amount of blood loss will be monitored closely to meet the guideline from the American Association of Blood Bank that no more than 525 mL will be collected in any eight-week period. The total estimated amount of blood loss for all the scheduled study visits is approximately 1165 mL for immunized volunteers over a 43-week period and 522 mL for control volunteers over a 16-week period.

Risks associated with *P. falciparum* malaria challenge

- Local inflammatory reactions to mosquito bites including immediate type (wheal and flare) and delayed type (pruritic papules)
- Assessment of the arm where mosquitoes bit
- Topical medication will be provided as needed.
- Systemic allergic reaction to mosquito bites, which is rare
- Trained personnel with emergency equipment and medications are available in the WRAIR insectary and CTC.
- Development of *P. falciparum* malaria infection
  - Transient abnormalities, such as fatigue, fever, chills, rigors, headache, myalgias, arthralgias, abdominal pain, nausea, vomiting, splenomegaly, and hepatic tenderness, are expected consequences of malaria
  - Untreated *P. falciparum* malaria can, in rare cases, lead to severe anemia, cerebral malaria or kidney, liver, heart, or brain damage, which can be life-threatening.
- Close observation, trained personnel, medications to treat infection and symptoms associated with malaria in a timely fashion has prevented such complications among more than 1000 subjects challenged at WRAIR/NMRC during the last 20 years.
- Death (exceedingly unlikely given the safeguards and controls in place).
- In challenges involving nearly 1000 study subjects over the last 20 years at the WRAIR/NMRC, there has never been a case of mortality or excess morbidity requiring inpatient hospitalization.
- Walter Reed Army Medical Center are alerted and provided with the name of specific subjects once they are challenged.
- Failure to completely eradicate the malaria infection with antimalarial drugs carries a risk of recrudescent malaria and/or one or more relapses before the natural healing of the disease
- Treatement discussed more fully in section 6.6
- Close follow-up including timely and direct observation therapy for all malaria treatment by trained personnel.
  - - Side effects from chloroquine or atovaquone/proguanil
  - Chloroquine can cause nausea, vomiting, stomach upset, cramps, loss of appetite, [diarrhea](http://www.medicinenet.com/script/main/art.asp?articlekey=1900), tiredness, weakness, ringing in the ear, or [headache](http://www.medicinenet.com/script/main/art.asp?articlekey=20628);
  - Atovaquone/proguanil can cause nausea, vomiting, diarrhea, headaches, blurred vision, pruritus, tinnitus, or photosensitivity

Risks associated with confidentiality

There is also the risk of a breach in confidentiality; however precautions will be taken to minimize this risk. All study communications and lab samples will be identified by a study code. These documents will not contain any study subject names or identifiable information. The subjects who agreed to be screened will be first assigned an alphanumeric screen code which includes their three initials and last four digits of social security number. If the subject is found to be eligible, they will be assigned a “subject code” which consists of the study number and a two digit number looking like “study number- ___ ___.” The same format for the codes for screened or enrolled subject will be used for lab samples sent to Quest Diagnostics, Inc.. Study information and records will be maintained in a secure storage area at the CTC for the duration of the study and then archived and eventually destroyed in a secure fashion as described in Attachment B.

In a few select circumstances, complete confidentiality cannot be guaranteed for subjects. These will include required reporting of transfusion-transmissible diseases (such as HIV, hepatitis, syphilis) to applicable military and public health agencies. All subjects will be informed of these exceptions to confidentiality during the consent process.

### *4.2.3 Risks to Study Personnel*

The principal risk in the clinical setting is in the handling of needles that may be contaminated with hepatitis, HIV, or other human pathogens. Adherence to universal precautions for working with infectious agents will reduce the risk of exposure to these individuals. There are no known risks to the environment other than those associated with the generation of biohazardous waste. Biohazardous waste will be discarded in a biohazard containment container and disposed of according to local, state, and federal regulations.

### *Transmission of Malaria to the Community*

The risk of transmitting malaria to the community will be negligible. The infected mosquitoes will be raised and the challenge will be conducted in the secure insectary at WRAIR. Any accidently exposed individuals will be presumptively treated with a standard malaria treatment.

- - 1. *Unknown Risks Associated with the Ad Vaccine*

There may be unknown or unanticipated risks associated with the study vaccine which have not been reported to the scientific community. If any new risk is reported, the subjects will be notified in a timely and comprehensive fashion. An example of unknown risk would be from a recent HIV vaccine study by Merck Inc. using adenovirus type 5 (Ad5) as the vaccine backbone. This HIV vaccine modifies the Ad5 in different ways (includes only two deletions in different locations) than the Ad malaria vaccine used for this study, and it produced HIV proteins not malaria proteins. This HIV study showed that particular HIV vaccine did not protect those engaged in very high risk behaviors (with up to 20 concurrent sexual partners and many are men having sex with men) from getting HIV. In addition, this HIV study suggested that subjects who had pre-existing Ad5 immunity and if they are also males who had not been circumcised may have a higher chance of getting HIV infections. This study did not provide a definitive assessment to the risk of getting HIV among immunized persons who do not engage in high risk behaviors. We also do not know about the risk for getting HIV infection from a non-HIV Ad 5 vaccine, more specifically a malaria vaccine with different transgene and different properties of the Ad5 vaccine. To avoid any potential risk of getting HIV, we strongly encourage the subjects who wish to participate in this malaria vaccine study to avoid participating in any high risk behaviors of getting HIV. The high risk behaviors will be assessed with the volunteers through one-on-one counseling by the clinical investigators. Those who have in the past or are currently engaging in high risk behaviors for getting HIV will be excluded from participation in this study. Subjects will be notified promptly if relevant risk information is learned in the future about the adenovirus vector and HIV infection.

- 1. **Measures to Minimize Bias**

Since there is only a single study group and lack of placebo vaccines, measures to minimize bias, such randomization or blinding, are not applicable and will not be used for this protocol.

- 1. **Justification of Sample Size**

There will be 20 subjects in the treatment group. If none of these subjects experience severe or serious vaccine related adverse events, then we have a confidence level of approximately 70% that the true rate of these events in the general population would be less than 5% (assuming everyone in the general population received the vaccine). These figures are determined by using an upper bound calculation for no events based on “The Rule of Three”: given no events in n trials, the 95% upper bound on the rate of occurrence is 3/n.[52] If the study group were further divided into those who are seropositive and seronegative, based on the assumption that there will be close to 1:1 ratio between these two groups, the level of confidence to detect true rate of adverse reactions that are less than 5%, 10%, and 20% are 45%, 70%, and 95% respectively. If the subject Ad5 serology status changes, it will be noted and analyzed primarily as cross-over (according to the new Ad5 level), but may be analyzed as intention-to-treat (according to the screening Ad5 level) as well. The cohort size of six was designed to correspond to previous malaria vaccine challenge studies performed at WRAIR/NMRC. This size was based upon prior statistical validation and the logistical limitations (e.g., physical space requirements) of the challenge model.

1. **Subjects and Cohorts**
   1. **Recruitment**

Subjects will be recruited from the general population in the greater Washington DC area. Recruitment will be in a non-coercive manner, and recruiting will take place either through the WRAIR CTC. Contact will be made with subjects already enrolled in the WRAIR CTC database as well as by advertising mechanisms such as word of mouth, flyers, emails, postings on public listservs, and posters aimed at adults living in the greater Baltimore-Washington region. Email announcements will include the same information as the flyer. The WRAIR CTC may place generic television advertisements recruiting subjects for participation in WRAIR CTC studies without mentioning specific studies. Materials used for recruitment will be prepared and submitted for review and approval by WRAIR IRB and the United States Army Medical Research and Materiel Command Human Subject Research Review Board (USAMRMC HSRRB) prior to initial use; any subsequent changes will need to be reviewed only by WRAIR IRB. See Attachments C, M, N, and O for recruitment plan including recruiting script, briefing, and flyers respectively.

Active duty military subjects will require approval from their supervisor through the division director using the Statement of Supervisor’s Approval (Attachment H).

- 1. **Informed Consent**

Subjects interested in participating will need to provide informed consent. They will meet with a member of the study team, review the details of the protocol, and ask and receive answers to any questions. Time will be provided to review the written informed consent document. When ready, the subject will sign the informed consent documents, including the HIV testing and blood donation consent forms (Appendix, Attachments E and F), and will complete an Assessment of Understanding (Attachment G). Subjects must score at least 80% correct on the multiple-choice Assessment of Understanding (14 questions for vaccinees and 10 questions for controls). If they do not score 80% on the initial quiz, the protocol information will be reviewed with them to ensure comprehension, and they will have the opportunity to retest. If a subject fails to correctly answer eight of the ten questions after three attempts they will be excluded from the study.

- 1. **Screening**

Subjects who have signed an informed consent form and who have successfully completed the Assessment of Understanding will provide a medical history and undergo a physical examination and routine laboratory screening tests which include the following:

- Complete blood count (CBC) with diff
- Serum chemistry profiles: glucose, creatinine, alanine aminotransferase (ALT), aspartate aminotransferase (AST), and total bilirubin
- Serological assays: antibody against hepatitis C and HIV (with reflex confirmatory testing done at Quest Diagnostics, Inc if the screening test is positive), hepatitis B surface antigen (HBsAg)
- Urine β-HCG (female subjects only)
- Electrocardiogram

The screening labs need to be done within 90 days prior to enrollment and repeated if that time is exceeded. All screening tests will be performed prior to entry into the study and at any other time during the course of the trial if judged necessary by the investigators. Screening and follow-up diagnostic laboratory studies will be performed in an accredited clinical laboratory, Quest Diagnostics, Inc. Screening history, physical examination, and laboratory findings will be recorded in the source documents for screening data. Subjects found to have significant abnormality will be counseled by the investigator, referred to a health care provider for further evaluation, and excluded from the study. The same screening procedure will be conducted for subjects to be enrolled in either the immunized or control groups.

There was a case in Holland who suffered heart disease after receiving an investigational malaria vaccine, treated with a medication that is not FDA-approved and underwent malaria challenge. That volunteer recovered uneventfully. There is no sufficient data to know for sure whether it could be due to any or all of the articles and procedures that volunteer experienced. To exercise extra precaution, we have voluntarily decided to assess the volunteers regarding their cardiac risk factors based on the Gaziano study [53] (attachment L) and obtain an electrocardiogram (EKG) on screening. Although the Gaziano group studied a much wider age range of 25-74 years, the study determined age 35 is the minimum age for formal assessment of potential elevated cardiac risks. However, as we do encounter volunteers younger than 35 years of age that are obese or have other risk factors, to be even more conservative from safety’s perspective, we will treat volunteers younger than 35 years of age as if they are 35 years old and evaluate all volunteers with the same methods provided in the Gaziano study. All volunteers need to be in no or low risk categories and have a normal EKG to be included in the study. Normal EKG is defined as absence of any clinically significant EKG changes, which may include normal variants. The EKG will be read on site by the investigators, then sent to a subject matter expert (a cardiologist or an internist with advanced training such as fellowship in cardiology). The EKG will contain only subject code, age, and gender; the latter two pieces of information will enhance accuracy of interpretation of EKG.

- 1. **Inclusion Criteria**

Subjects must meet these criteria for inclusion in the study:

- Healthy adults 18 to 50 years of age (inclusive)
- Women who are not pregnant by a current negative pregnancy test or of non-childbearing potential
- Willing to use an FDA approved birth control method including condoms, birth control pills, sterility surgery, or intrauterine devices among others, from time of enrollment until 6 months after the end of the active phase of the study
- Able to provide free and willing written informed consent to participate
- Score at least 80% correct on Assessment of Understanding (14 questions for vaccinees and 10 questions for controls)
- No plans to travel to a malaria endemic area during the course of the study
- Free of significant health problems as established by medical history and clinical examination completed prior to the study
- Available to participate and reachable for duration of study (up to five years)
- Only subjects with no or low cardiac risk factors according to the Gaziano study [53] and a normal EKG will be included in the study
  1. **Exclusion Criteria**

Subjects cannot meet any of these criteria below during the study period. If any exclusion criteria were noted before any immunization, the subject will be removed from the study and alternate subjects will be enrolled instead.

- Pregnant (positive β-HCG) or nursing at screening or plans to become pregnant or nurse from the time of enrollment until 6 months after sporozoite challenge
- Any past history of malaria
- History of receipt of malaria vaccine
- Plans to travel to malarious areas during the study period
- Use of any investigational or non-registered drug or vaccine within 30 days prior to enrollment
- Seropositive for HIV, hepatitis C virus (antibodies to HIV and HCV), and/or HBsAg
- Subjects in the immunized group who engage in high-risk behaviors for acquiring HIV
- Allergy to antimalarials or significant (e.g. systemic) hypersensitivity reactions to mosquito bites (local hypersensitivity reactions at the site of a mosquito bite are not an exclusion criterion)
- History of psoriasis (given its interaction with chloroquine)
- Use or planned use of any drugs with significant anti-malarial activity, such as doxycycline, clindamycin, azithromycin, or trimethoprim/sulfamethoxazole among others during the study period (subjects can withhold the use of these medications during the study period if approved by their primary care physicians, at the minimum starting from four weeks before challenge until four weeks after becoming parasitemic)
- Any confirmed or suspected immunosuppressive or immunodeficient condition, including HIV infection and history of splenectomy
- Administration of chronic (defined as more than 14 days) immunosuppressants or other immune-modifying drugs within six months of challenge
  - For corticosteroids, this is defined as prednisone, or equivalent, 0.5 mg/kg/day;
  - Inhaled and topical steroids are allowed
- A family history of congenital or hereditary immunodeficiency
- Chronic or active neurologic disease including seizure disorder
- Acute or chronic, clinically significant pulmonary, cardiovascular, hepatic, or renal functional abnormality, as determined by medical history, physical examination, or abnormal baseline laboratory screening tests:
  - ALT above normal range (table 7)
  - Creatinine above normal range (table 7)
  - Hemoglobin below normal range (table 8)
  - Platelet count below normal range (table 8)
  - Total white cell count below normal range (table 8)
- Abnormal baseline EKG obtained at screening
- Acute disease at the time of enrollment
  - Acute disease is defined as the presence of a moderate or severe illness with or without fever
  - Challenge can be administered to persons with a minor illness, such as diarrhea or mild upper respiratory infection without fever (i.e., oral temperature < 38°C/100.4°F)
- Hepatomegaly, right upper quadrant abdominal pain or tenderness: noted by physical exam during the screening process.
- Administration of immunoglobulins and/or any blood products within the three months preceding immunization during the study period
- Allergy to kanamycin or related antibiotics
- Suspected or known current alcohol abuse/drug abuse as obtained by history and physical examination
- Inability to make follow-up visits
- Any other significant finding that in the opinion of the investigator would increase the risk of having an adverse outcome from participating in this study
  1. **Elimination Criteria**

The following criteria should be checked at each visit after the screening visit and any subject meeting any of these criteria will be withdrawn from the study:

- Use of any investigational or non-registered product (drug or vaccine)
- Use or planned use of any drugs with significant anti-malarial activity
- Administration of chronic (defined as more than 14 days) immunosuppressants or other immune-modifying drugs one month prior to any immunization or challenge
- Administration of immunoglobulins and/or any blood products during the study period
- Clinical evidence of drug abuse, intoxication during follow-up visits, or other medical condition that would increase the risk of an adverse event or possible harm to the subject from further participation in this study
- Poor reliability or inability to keep appointments
- Becoming pregnant or failure to practice an effective form of birth control
- Travel to malarious areas during the study period
- Any other concern of the PI or other investigator that precludes subject involvement in the study

If the above elimination criteria were noted after receipts of any immunization or after the challenge, the subject will still complete the rest of the visits which are intended to monitor the safety of the subjects. The data from these subjects will be analyzed separately especially if it was one of the first four criteria that occurred since these four conditions will affect the immunologic response and the outcome of the study.

- 1. **Subject Completion**

When all the follow-up visits for the subjects are completed for both of the active stage and long-term stage of follow-up, they will be notified that they have completed the study and they will be officially withdrawn from the study. All subjects who were challenged will be instructed to contact the study team anytime within a year post challenge if they experience a fever. This is an extra precaution for recurrent malaria. In addition, challenged subjects will not be able to donate blood for 3 years per the American Red Cross guidelines.

- 1. **Subject Dropout**

A subject may end his or her participation in the study at any time or the investigator may elect to withdraw a subject; however clinical follow-up after a challenge is important to ensure the safety of the subject, even if they no longer consent to have their information used for research purposes. Subjects who withdraw from the study are required to complete clinical follow-up visits and anti-malaria treatment for safety purposes. Since untreated malaria infection could have severe consequences including death, subjects will be sufficiently counseled for this requirement as indicated in the informed consent form and advised not to participate if they do not think they can meet this requirement. Whenever a subject is withdrawn or withdraws, the investigator will complete the study termination CRF (case report form; all CRFs can be found in Attachment P) specifying the reason the subject left the study. If a subject withdraws, the investigator will make a reasonable effort to determine the reason for the subject’s withdrawal from the study and to complete termination procedures. Telephone calls, letters, and/or e-mail correspondence are considered reasonable effort. All data collected up to the time of withdrawal will be reported and stored as all other study data. Withdrawing from the study for any reason will not impact the subject’s medical care. The subject could elect to have the remaining blood samples destroyed. At this time, there is no specific information that will be collected on the withdrawn subjects. The only exceptions are subjects with ongoing adverse events and women who become pregnant during, or within 2 months of the conclusion of the active stage of following up. If a subject becomes pregnant before any immunization or the challenge, the subject will be excluded. If a subject becomes pregnant, the subject would be encouraged to see an obstetric care provider for follow-up.

If subjects leave the study prior to the passage of 3 months post challenge and move outside of the Washington, D.C. area, they will be instructed to inform their medical care providers that they have been exposed to malaria and must have a blood smear checked whenever they develop a fever. In addition, they should request that their medical care providers contact the study physicians for advice concerning the diagnosis and treatment of malaria infection. If they leave the study prior to the passage of 3 months post challenge but remain in the area, they will be instructed to contact the study team whenever they have a fever within one year after a challenge so that a blood smear can be checked and treatment can be provided if necessary.

1. **Conduct of the Study**

Dr. Chuang is the principal investigator of this study and is responsible for the conduct of the protocol, the actions of all other investigators, the safety of the subjects, and all documentation and reporting. Associate physician investigators will be involved in screening of patients, consenting subjects, recording of solicited and unsolicited symptoms, and evaluation of adverse events. All of the clinical investigators are licensed and credentialed physicians.

Dr. Arthur Lyons is the medical monitor for this study. He is a qualified physician not associated with this protocol who is able to monitor the subjects during the conduct of the study and ensure that adequate medical care is provided for conditions that may arise during the study. He will review all serious adverse events as well as serious or severe unexpected adverse events associated with the protocol and provide an unbiased written report of the event within ten calendar days of the initial report. At a minimum, the medical monitor will comment on the outcomes of SAE and relationship of the SAE to the challenge. The medical monitor will also indicate whether he concurs with the details of the report provided by the study investigator.

A Sponsor-designated study monitor will also be assigned to this study. This study monitor from USAMMDA will conduct visits before, during and at the conclusion of the study in order to evaluate the conduct of the study.

For investigation product preparation, administration, storage, and disposition, please refer to the study specific procedures as listed in attachment D.

## Investigational Product Preparation, Storage, and Disposition

The vaccine supplies are stored at NMRC’s contract storage/distribution facility, ThermoFisher BioServices, Rockville MD, at -70˚C (+ 10˚C). Prior to each immunization day, a quantity of vaccine sufficient for that day of immunizations will be delivered by ThermoFisher BioServices to the WRAIR CTC. The vaccine should be shipped on dry ice with a temperature monitor within a validated transport temperature range. The CTC staff will record receipt of the vaccine which will be stored in a -70˚C (+ 10˚C) freezer at the Clinical Trials Center. The freezer in the Clinical Trials Center is equipped with an external temperature display for temperature monitoring and a telephonic alarm system should temperatures go outside of specified ranges. If deviations in storage temperature occur, the principal investigator will report the deviation promptly to the IND sponsor. For the Ad vaccine, since the shipping package includes dry ice, the vials will need to be sealed in Mylar bags because CO_2_ can affect the pH and thereby inactivate the adenoviral vector.

The DNA vaccine is in 2 mL glass vials with stoppers filled to 1.2 mL with the intended deliverable volume of 1 mL. It is stored as frozen liquid between -10˚C and -90˚C, and when thawed it should be clear. The DNA vials are intended for single use only, and partially used vials will not be administered to other subjects. No mixing or other processing is needed. The DNA vaccines will be administered by Biojector®; proper training on use of Biojector® will be provided prior to the first DNA dose. The trained personnel will draw 1 mL from one vial into a syringe and loaded to Biojector® prior to intramuscular administration to one arm; the same procedure will be repeated from another vial for administration to the other arm. The recommendation from Vical regarding non-adjuvanted vaccines such as our DNA vaccine, is to be used within 12 hours of thawing. However, this data has not been derived from a stability study specifically with our DNA vaccine.  As a result, we have decided to use the vaccine within 8 hours after thawing.  Any vaccine not used after thawing will be discarded and recorded as such for monitoring and accounting purposes. The empty vials and unused portions will be discarded as medical waste in a biohazard containment bag. Any unopened vials that remain at the end of the study will be returned to the storage facility or discarded at the discretion of the study sponsor.

The Ad vaccine consists of two antigens, CSP and AMA1 (0.6mL/vial at 1 x 10^11^ *pu*/mL) and study diluent supplied by the manufacturer. The diluent (FFB) is an aqueous buffered saline solution. The vaccines are vialed at a volume of 0.6 mL in a 1.5 mL polypropylene cryovial each. On the day of Ad vaccine administration, the vaccine will be prepared by appropriately trained personnel. Equal volumes of both vaccine antigens, 0.55 mL each, will be placed into a single 5 mL glass sterile vial container and mixed. Four mL of the study diluents will be added to the combined vaccine vial for a final volume of 5.1 mL, sufficient for 4 injections of 2 x 10^10^ *pu*. One mL of the mixed solution containing both CSP and AMA1 antigens (1 x 10^10^ *pu* for each antigen) will be administered intramuscularly by conventional needle. The mixed solution must be used within 4 hours after removal from the freezer. Any vaccine not used within 4 hours of thawing will be discarded and recorded as such for monitoring and accounting purposes. Once thawed, vials should not be refrozen or reused outside the recommended period. Vials of the study diluent may be stored for the duration of the study (not to exceed two years) at -10 to -45˚C. The empty vials and the unused portions of an Ad vaccine vial will be handled in the same fashion as the DNA vaccine.

- 1. **Infecting Mosquitoes with *P. falciparum* Parasites**

The malaria strain used for this challenge, *P. falciparum* strain NF54/clone 3D7, is a cultured human malaria isolate. It is susceptible to currently approved malaria treatments and has previously been used in the WRAIR/NMRC challenge model to successfully infect human subjects. Master seed lots of these parasites were developed and are stored at WRAIR/NMRC. All blood products used for production of infected mosquitoes have been commercially screened for HIV, hepatitis B and C, and syphilis.

The mosquitoes used will be laboratory born and reared *Anopheles stephensi* screened for infectious agents. This species is relatively easy to maintain in the laboratory for up to 21 days after infection with malaria, readily feeds on humans, and is able to transmit malaria to humans^38^. Mosquitoes will be infected by allowing them to feed through membranes on cultures of *P. falciparum* containing a large proportion of gametocytes. One week after infection, 20 mosquitoes will be removed and dissected to estimate the malaria infection rate. If the rate is less than 50%, attempts to infect mosquitoes will be repeated using a new batch of mosquitoes and a fresh malaria culture. Approximately 16 days after successful infection, ten mosquitoes will be removed daily and dissected to look for sporozoites in their salivary glands. If the mean sporozoite density is less than 100 per mosquito, attempts to infect mosquitoes will be repeated using a new batch of mosquitoes and a fresh malaria culture until the sporozoite density is at least 100 per mosquito or greater.

- 1. **Detailed Description of Study Phases and Visits**
     1. Screening and Enrollment Phases

Prior to the first dose of immunization, up to 23 subjects will be enrolled for the immunized group. Because of the long prime-boost interval of four months and concern of volunteer drop-out, the control group will not be recruited until three months prior to the Ad boost. All subjects enrolled in the study will be issued an emergency notification card that details their participation in the study and provides specific contact phone numbers of the investigators. Vital signs (temperature, pulse, blood pressure) are recorded at all scheduled study visits unless indicated otherwise. All efforts will be made that immunizations and challenge for all subjects will occur on the same day. However, the blood draw scheduled on the day of immunization or challenge could occur a couple of days earlier to ease potential congestion on the days of these major events. The following account of study visit numbers refers to immunized subjects only for the sake of simplicity; the corresponding study visit number for the control subjects can be found in Table 1b above. Day numbers refer to the numeric day of study duration, not study date.

| Visit 1: Subject Screening and Baseline Assessment | Day -90 to -3 |
| --- | --- |

- Briefing of the potential subject for both immunized and control groups
- Written Informed Consent (Appendices 1 or 2 for immunized and control subjects respectively), including HIV testing (Attachment E) and blood donation (Attachment F)
- Comprehension assessment (Attachment G)
- Provision of medical history by subject
- Check of inclusion and exclusion criteria
- Vital signs and physical examination
- Urine β-HCG pregnancy test for all female subjects
- Whole venous blood sample collection for screening lab tests: approximately 34 mL
  - Serology: Antibody to HIV and hepatitis C, HBsAg
  - Complete blood count with differential
  - Serum chemistry: glucose, creatinine, ALT, AST, and total bilirubin
- Electrocardiogram (EKG)

**Immunization Phase**

The first dose of the DNA and the Ad vaccines will be given in a staggered fashion as a safety precaution. Two volunteers will receive the vaccine first; the rest will receive the vaccine no earlier than 3 hours after the initial two volunteers or in the next day.

| Visit 2: **DNA immunization #1** | **Day 0 (+ 3 day)** |
| --- | --- |

- Verify inclusion/exclusion criteria
- Vital signs, history and history-directed physical examination
- Collection of urine for pregnancy tests where applicable
- Blood collection: approximately 180 mL whole venous blood for the determination of:
- Safety labs (7ml) : CBC, creatinine, AST, ALT
- Ad5 titer and HLA typing (5 mL and 20 mL respectively)
- 10 mL whole venous blood for humoral immune studies, storage and transport condition per study specific procedure.
- 125 mL whole venous blood for CMI assays, storage and transport condition per study specific procedure.
- 8 mL for microarray

The Ad5 titer will be assessed by neutralization assay at NVITAL, and is considered negative if it is less or equal to 500 and positive if higher than 500. For HLA typing, specimen from either blood or buccal smears is acceptable; assumption for using blood is made for now for calculation of blood draw volume. Of note, since Ad5 and HLA typing are only applicable to enrolled volunteers, they will be done on day 0, not at time of screening.

- Immunization: Intramuscular (IM) administration of the DNA vaccine by Biojector® via two injections, 1 mL in the deltoid muscle of each arm

NOTE: Each vaccinee will be closely observed for at least 30 minutes following immunization. After observation, blood pressure (BP), pulse, and oral temperature will be taken and solicited and unsolicited symptoms will be reviewed. Subjects will be given an emergency contact card and instructed to contact the study team immediately should they manifest any signs or symptoms they perceive as serious.

| **Visits 3-6: Post-immunization 1 follow-up (f/u) visits** | **Days 1, 2, 7 (±3 day)**  **Day 14 (±5 day)** |
| --- | --- |

- Check of elimination criteria
- Review of history and medications
- Vital signs. If Grade 3 adverse reaction (AE) is noted, the PI or other physician investigator must be notified.
- Recording of solicited and unsolicited AE, and history directed physical exam
- Blood collection:
  - **On day 7**, safety lab (7ml): CBC, creatinine, AST, ALT
  - **On day 14**, humoral immune studies (10 mL). The sample will be processed as detailed above.

| **Visit 7: DNA Immunization #2** | **Day 28 (±3 days)** |
| --- | --- |

- Check elimination criteria, contraindications/precautions and record medications
- Record any unsolicited adverse events occurring after the last vaccine dose
- Vital signs, history, directed physical examination
- Collection of urine for pregnancy tests where applicable
- Blood collection:
  - - Safety lab (7 mL): CBC, creatinine, ALT and AST
    - Humoral immune studies (10 mL), storage and transport condition per study specific procedure.
- Immunization: IM administration of the DNA vaccine by Biojector® via two injections, 1 mL in the deltoid muscle of each arm.

NOTE: Each vaccinee will be closely observed for at least 30 minutes following immunization after which blood pressure (BP), pulse, and oral temperature will be taken and solicited and unsolicited symptoms will be reviewed by the PI or the designee. Subjects will be instructed to contact the investigator immediately should they manifest any signs or symptoms they perceive as serious. Subjects will be provided with a thermometer to take the temperature once every night for 7 nights from day 0 through day 6 and provide recordings on the volunteer temperature log to the study team during the f/u visits on day 1, 2, and 7.

| **Visits 8-11: Post-immunization 2 f/u visits** | **Days 29, 30, 35 (±3 days)**  **Day 42 (±5 days)** |
| --- | --- |

- Check of elimination criteria
- Review of history and medications
- Vital signs. If Grade 3 adverse reaction (AE) is noted, the PI or other physician investigator must be notified.
- Recording of solicited and unsolicited AE, and history directed physical exam
- Blood collection:
  - **On day 35** (7 days after DNA #2), safety lab (7ml): CBC, creatinine, AST, ALT
  - **On day 42** (14 days after DNA #2), humoral immune studies (10 mL), storage and transport condition per study specific procedure.

| **Visit 12: DNA Immunization #3** | **Day 56 (±3 days)** |
| --- | --- |

- Check elimination criteria, contraindications/precautions and record medications
- Record any unsolicited adverse events occurring after the last vaccine dose
- Vital signs, history, directed physical examination
- Collection of urine for pregnancy tests where applicable
- Blood collection, approximately 25 mL whole venous blood will be collected for the determination of:
  - - Safety lab (7 mL): CBC, creatinine, ALT and AST
    - Humoral immune studies (10 mL), storage and transport condition per study specific procedure.
    - Microarray (8 mL)
- Immunization: IM administration of the DNA vaccine by Biojector® via two injections, 1 mL in the deltoid muscle of each arm

NOTE: Each vaccinee will be closely observed for at least 30 minutes following immunization after which blood pressure (BP), pulse, and oral temperature will be taken and solicited and unsolicited symptoms will be reviewed by the PI or the designee. Subjects will be instructed to contact the investigator immediately should they manifest any signs or symptoms they perceive as serious.

| **Visits 13-18: Post-immunization 3 f/u visits** | **Days 57, 58, 63 (±3 days)**  **Days 70, 84 (±5 days)**  **Day 126 (±7 days)** |
| --- | --- |

- Check of elimination criteria
- Review of history and medications
- Vital signs. If Grade 3 adverse reaction (AE) is noted, the PI or other physician investigator must be notified.
- Recording of solicited and unsolicited AE, and history directed physical exam
- Blood collection:
  - **On day 57 and 58** (days 1 & 2 after DNA #3), microarray (8ml)
  - **On day 63** (day 7 after DNA #3), safety lab (7ml): CBC, creatinine, AST, ALT
  - **On day 70** (day 14 after DNA #3), humoral immune studies (10 mL).
  - **On day 84** (day 28 after DNA #3), 142 mL of whole blood will be collected for:
    - Safety labs (7ml) : CBC, creatinine, AST, ALT
    - 10 mL whole venous blood for humoral immune studies, storage and transport condition per study specific procedure.
    - 125 mL whole venous blood for CMI assays, storage and transport condition per study specific procedure.
  - **On day 126** (day 70 after DNA #3), safety lab (7ml): CBC with diff, creatinine, AST, ALT

| **Visit 19: Ad5 Immunization** | **Day 168 (±7 days)** |
| --- | --- |

- Check elimination criteria, contraindications/precautions and record medications
- Record any unsolicited adverse events occurring after the last vaccine dose
- Vital signs, history, directed physical examination
- Collection of urine for pregnancy tests where applicable
- Blood collection: approximately 160 mL whole venous blood for the determination of:
- Safety labs (7ml) : CBC with diff, creatinine, AST, ALT
- 10 mL whole venous blood for humoral immune studies, storage and transport condition per study specific procedure.
- 125 mL whole venous blood for CMI assays, storage and transport condition per study specific procedure.
- 8 mL for microarray
- 5 mL for Ad titer
- Immunization: 1 mL of the Ad5 vaccine will be administered intramuscularly in the deltoid muscle of the non-dominant arm

NOTE: Each vaccinee will be closely observed for at least 30 minutes following immunization after which blood pressure (BP), pulse, and oral temperature will be taken and solicited and unsolicited symptoms will be reviewed by the PI or the designee. Subjects will be instructed to contact the investigator immediately should they manifest any signs or symptoms they perceive as serious.

| **Visits 20-23: Post-immunization 4 f/u visits** | **Days 169, 170, 175(±3 days)**  **Day 182 (±5 days)** |
| --- | --- |

- Check of elimination criteria
- Review of history and medications
- Vital signs. If Grade 3 adverse reaction (AE) is noted, the PI or other physician investigator must be notified.
- Recording of solicited and unsolicited AE, and history directed physical exam
- Blood collection:
  - **On days 170** (day 2 after Ad), microarray (8ml)
  - **On day 175** (day 7 after Ad), safety lab (7ml): CBC with diff, creatinine, AST, ALT
  - **Day 182:** 5 mL for Ad titer

The following are applicable for any of the f/u visits after the Ad immunization:

- - - - 1. Swabs of nasopharynx or conjunctivae or samples of urine or stool will be collected if a subject is found to have an upper respiratory infection, conjunctivitis, urinary tract infection or inflammation, or gastrointestinal illness.
        2. Cultures from any samples positive for adenovirus will be sent to GenVec, Inc to assess for the presence of specific sequences from the vaccine vector using PCR analysis

During the visit immediately prior to the challenge, subjects will be instructed that on the day of challenge not to wear cologne, perfume, or aftershave or use scented soaps as it may discourage mosquito feeding.

| **Visit 24: Challenge** | **Day 196 (± 3 days)** |
| --- | --- |

- Check of inclusion and exclusion criteria, elimination criteria and contraindications to challenge
- Review medical history and medications since previous visit
- Record pre-challenge data including oral temperature, pulse, and blood pressure
- Directed physical examination
- Urine pregnancy test for females
- Blood volume: approximately 156 mL
- 2 mL for PCR, 8 ml microarray, 5 mL Ad 5 titer
- 7 mL safety labs: CBC with diff, creatinine, AST, ALT
- 10 mL whole venous blood for humoral immune studies.

-- 125 mL whole venous blood for CMI assays.

For control volunteers, HLA typing (20 mL) will also be performed on the day of challenge.

Sporozoite Challenge: Will take place in the WRAIR insectary and the immunized and control subjects will be treated in an identical fashion. For each subject, five mosquitoes will be allowed to feed on the forearm of the subject over five minutes. These mosquitoes will be dissected to confirm they were infected. If all five are not adequately fed or infected (+2 gland grade or better according to WRAIR insectary protocol), additional mosquitoes will be allowed to feed to ensure a total of five infected mosquitoes having fed on the subject.

Post-challenge: Each subject will be closely observed for at least 30 minutes following the challenge, after which blood pressure, pulse and oral temperature measurements will be taken. Any reactions will be reviewed, promptly treated, and noted in the subject record*.* Subjects will be provided emergency notification card (Attachment R) which includes all relevant contact information in case of emergency.

Post-Challenge Phase

| **Visits 25: Post-Challenge Week 1 f/u visits** | **Days 3 Post-Challenge (± 3 days)** |
| --- | --- |

- Microarray (8 mL)

| **Visits 26-30: Post-Challenge Week 1 f/u visits** | **Days 6 to 9 Post-Challenge (± 3 days)** |
| --- | --- |

- Check of elimination criteria
- Review of history and medications
- Record oral temperature, pulse, and blood pressure
- If Grade 3 adverse reaction (AE) is noted, the PI or other physician investigator must be notified.
- Recording of solicited and unsolicited AE, and history directed physical exam
- Blood collection:
- Day 6 after challenge until volunteer has 3 consecutive negative smears after becoming parasitemic, 4 mL whole venous blood once a day plus finger or venous stick once a day for:
- Malaria smears, PCR
- Once subject becomes parasitemic, more frequent safety blood draw may be needed as determined by the investigators.

If subjects develop symptoms or found to have positive blood smears before or after the hotel phase, the subjects will be evaluated at the CTC and treated immediately based on the study treatment regimen.

| **Visit 31-40: Post-Challenge: Hotel Phase** | **Days 10 - 19 Post-Challenge (± 2 days)** |
| --- | --- |

- Beginning on the night of Day 9, participants will be required to check into a designated hotel in the general vicinity of WRAIR/NMRC every evening for approximately 10 nights. An assessment room will be designated and staffed 24 hours a day with trained personnel. Investigators will be on call and available to provide medical care 24 hours each day. Daily history and directed physical examination will be performed (to include vital signs) every morning. All subjects will be asked about the presence of malaria-specific symptoms: fever, malaise/fatigue, chills/rigors, headache, myalgias, arthralgias, nausea, vomiting, diarrhea, abdominal pain. In addition, an open-ended question “do you have any other symptoms?” will be asked to collect unsolicited adverse events. Subjects will be contacted by telephone or in person each afternoon to review the same morning assessment questions until they become positive for malaria. At any time required, the on-duty investigator will arrange for the timely production of blood smears, along with their examination and interpretation, in order to treat those subjects in whom therapy for malaria is indicated rapidly. Once a positive smear is identified and treatment is initiated, daily blood smears will continue to be obtained until three consecutive films are negative. When subjects become parasitemic, they will be treated immediately based on the study treatment regimen.
- Recording of vital signs including oral temperature, pulse, and blood pressure.
- History, directed physical exam as needed
- Solicited and unsolicited AE/SAE will be reviewed and recorded
- Blood draws with approximate amounts:
- Day 6 after challenge until volunteer has 3 consecutive negative smears after becoming parasitemic, 4 mL whole venous blood once a day plus finger or venous stick once a day for:
- Malaria smears, PCR

Once subject becomes parasitemic, more frequent safety blood draw may be needed as determined by the investigators.

- Follow-up after becoming parasitemic:
- See section with visit numbers 41 – 43.

Additional examinations may be performed should a participant exhibit any symptoms suggestive of malaria. The range of clinical tests performed will be at the discretion of the clinical investigators.

- If a participant is found to be parasitemic on microscopy (diagnostic criteria for malaria in this study):
- Antimalarial treatment will be initiated as described in section 6.6.
- After the third negative smear after treatment, the investigator may excuse the subject from nightly check-in at the hotel.

**Additional Visits: Day 20-28, post-challenge visits for subjects who have not developed malaria on day 19**

Subjects who have remained negative for malaria during the hotel stay will be seen every other day for evaluation in the Clinical Trial Center during the week (Monday through Friday -weekends and holidays may excluded, unless a visit is determined to be necessary by PI) for evaluation. If a subject does not keep a scheduled appointment, they will be contacted by telephone.

If needed, the investigator can increase or decrease the frequency of the visits depending on the subjects’ clinical condition. Additional examinations may be performed at the discretion of the investigator should a participant exhibit any symptoms suggestive of malaria. Any subject diagnosed with malaria will be treated and followed as noted in the hotel phase. Any subjects requiring these optional visits will rejoin the rest of the cohort in scheduled follow-up after Day 28.

At each visit:

- Check of elimination criteria
- Review of history and medications
- Vital signs. If Grade 3 adverse reaction (AE) is noted, the PI or other physician investigator must be notified.
- Recording of solicited and unsolicited AE, and history directed physical exam
- Blood collection
- From day 20 to 28 post challenge, 4 mL whole venous blood every other day for malaria smears, PCR
- Follow-up after becoming parasitemic: see section on visit numbers 41-43.

| **Visit 41-43: Post-Challenge f/u visits** | **Days 21, 23 (+ 1 day),**  **Day 28 (+ 3 days)** |
| --- | --- |

- Check of elimination criteria
- Review of history and medications
- Vital signs. If Grade 3 adverse reaction (AE) is noted, the PI or other physician investigator must be notified.
- Recording of solicited and unsolicited AE, and history directed physical exam
- Blood collection, on day 28,
  - safety (7 mL, CBC with differential, creatinine, AST, ALT)
  - humoral immunity (10 mL) and cellular immunity (125 mL)

If a volunteer is still not parasitemic on day 28 post challenge, due to potential severity of the *P. falciparu*m infection, the volunteer will be treated presumptively in the same fashion as above.

| **Visit 44: – Final In-Person Study visit** | **Post challenge day 84,**  **Study day 280 (± 14 days)** |
| --- | --- |

- Check of elimination criteria
- Review of history and medications
- Vital signs. If Grade 3 adverse reaction (AE) is noted, the PI or other physician investigator must be notified.
- Recording of solicited and unsolicited AE, and history directed physical exam
- Blood collection: approximately 142 mL whole venous blood for the determination of:
- Safety labs (7ml) : CBC with diff, creatinine, AST, ALT
- 10 mL whole venous blood for humoral immune studies, storage and transport condition per study specific procedure.
- 125 mL whole venous blood for CMI assays, storage and transport condition per study specific procedure.

**Visits 44+: Intervals Determined by Investigators --- Adverse Events Related Visits**

These visits only apply to subjects who still have ongoing AEs on study day 305 and require continued follow-up until stabilization of AEs. The frequency and intervals of these visits will be determined by the investigators. Subjects with ongoing AE will be followed until AE is stabilized.

- Review of medical history and medication since previous visit
- Review and recording of any AE/SAE
- Recording of vital signs including oral temperature, pulse, and blood pressure.

Long-term Follow-up Phase

| **Annual phone, email, US mail f/u** | **Days 670, 1035, 1400, 1765 (± 60 days)** |
| --- | --- |

Per FDA’s request, all the immunized subjects will be followed by phone, email, or US mail annually after the last in-person visit for a total of five years starting with the first immunization. The subjects will be asked about whether any significant change of medical history has occurred since the last contact with the study team, which will be properly reviewed and recorded. Interval illness and adverse events will be recorded in source documents but will not be included in the study database. The exception to this would be if the principal investigator (PI) identifies an adverse event that may be related to the vaccine. In the case that the subjects do not respond to initial contact, three attempts of contact is considered reasonable efforts. After the last f/u contact is established or after three failed attempts to reach the subject, the subjects will be officially dis-enrolled from the study. Any time a subject departs from the study at or prior to conclusion of the study, the reason for departure will be recorded.

- 1. **The Day of the Challenge**

On the day of challenge prior to the challenge procedure, the study staff will re-emphasize to study subjects the importance of disclosing any experienced symptoms or changes in health status. The challenge will take place in the WRAIR insectary, within a carefully controlled environment. Five mosquitoes will be placed in a pint carton with a screened top, and the subject will lightly rest their forearm on the screen for five minutes. Their arm will be covered with a towel to simulate dusk feeding conditions. After the mosquitoes have completed feeding, they will then be dissected to confirm the presence of a blood meal and determine the infectivity rate and the salivary gland score. Each subject must receive a total of five bites from mosquitoes with a minimum salivary gland score of two or more (> ten sporozoites in the salivary gland). The salivary gland score rates the mosquito’s salivary gland for the presence of sporozoites. The assessment is based on a scale from 0 to 4+ as specified in the WRAIR insectary SOP. If less than five mosquitoes take a blood meal from the subject or those that did feed have a salivary gland score of less than 2+, then the mosquitoes that did not feed or that were insufficiently infected will be replaced with new, unfed mosquitoes and the feeding procedure repeated. This procedure will be repeated until the subject receives all five qualifying bites.

Subjects will be observed for 30 minutes following completion of the sporozoite challenge in order to assess them for any evidence of acute allergic reactions related to mosquito exposure. No severe allergic reactions related to mosquito bites have been documented in a WRAIR malaria challenge. Transient local allergic reactions (itching, rash) typical of mosquito bites will likely occur at the bite sites. Any reactions will be treated with appropriate medications, if necessary.

- 1. **Determination of Parasitemia**

Parasitemia will be determined by microscopy of Giemsa-stained thick blood films (smear) according to SOP. A microscopist will examine 360 hpf for *P. falciparum* parasites. A minimum of two malaria parasites indicate a positive smear, which needs to be confirmed by an expert microscopist. If the parasites are confirmed, microscopists will immediately relay all positive smear results to the on-call physician. If a subject is symptomatic and fewer than two parasites are found in the first 360 hpf, the microscopist will examine up to 1,000 hpf to identify the second parasite. If a second parasite is found, the subject will be treated. If a second parasite is not found, even if the subject appears to be symptomatic, the subject will not be treated and other causes of symptoms will be thoroughly considered; ultimately the investigators will make the decision regarding treatment based on all the findings. If an immunized subject is treated without demonstration of parasitemia, data pertaining to him/her will not be used in the primary efficacy analysis based on positivity/negativity of thick blood films. However, other data, including those relevant to time to positive PCR will be used as appropriate. The subject will be treated based on microscopy results, not PCR results. Smear results will be correlated with qRT-PCR results from the same sample to evaluate the potential of qRT-PCR to detect parasitemia earlier than microscopy.

- 1. **Management of Subjects Post-challenge**

After the challenge, all subjects will be reminded of the symptoms of malaria and the effects of untreated malaria. Active duty subjects will receive strict instructions (whereas civilian subjects will be highly encouraged) not to travel outside of the Washington, D.C., metro area from day 10 to day 19 post-challenge unless they have already acquired malaria and have been treated. If a subject must travel, proper arrangements will be made to ensure maximum contact (e.g. numbers of mobile phone, pager, home phone, etc.) between the subject and the study team, and if needed, the subject will be withdrawn from the study and presumptive treatment with chloroquine will be provided.

The prepatent period for human *P. falciparum* infection normally ranges from 9-14 days, so each morning after the challenge from day 6 to day 19 subjects will be evaluated by a study physician for the signs and symptoms of malaria and a blood sample will be evaluated for parasitemia. It is likely that all subjects will develop blood stage malaria, so a hotel stay from day 10 to day 19 post-challenge will be required for close monitoring and rapid assessment. An investigator will be in the hotel each night during the in-hotel phase. During the day, the investigator may be off-site but will still be accessible via phone or pager, and qualified study personnel will be in the hotel 24 hours per day. The symptoms developed during the expected patency period after the challenge which are consistent with malaria infection will NOT be recorded as adverse events. The PI can make the determination that it is safe for a particular subject to spend one or more nights during the monitoring period at his or her home. Since post-challenge symptoms consistent with malaria are expected, they are not considered as adverse events. These symptoms as listed in Table 2 will be recorded in the CRF for internal monitoring purpose for comparison of all malaria challenges. The monitoring period for the malaria symptoms will be from day 6 through the third negative malaria smears for volunteers who do develop symptoms prior to day 28 post challenge, or up to day 28 post challenge at the time of empiric treatment.

Table 2. Post-challenge malaria symptoms

| **General** | Fever* (Oral Temperature) |
| --- | --- |
|  | Malaise/Fatigue |
|  | Chills/Rigor |
|  | Headache |
|  | Myalgia |
|  | Arthralgia |
|  | Nausea |
|  | Vomiting |
|  | Diarrhea |
|  | Abdominal pain |
|  | Lower back pain |
|  | Other |

N.B. . * Fever is defined as oral temperature ≥ 38°C (100.4 °F).

Treatment: Since the NF54/3D7 challenge strain of *P. falciparum* is chloroquine-sensitive, subjects who develop blood stage *P. falciparum* infection during follow-up will be treated with a standard dose of chloroquine. The standard dose is a total of 1500 mg chloroquine base given orally in divided doses: 600 mg initially, followed by 300 mg given on days 0, 1, and 2 each. If any subject has a history of inability to tolerate chloroquine (including allergic reaction), he/she would receive treatment with the alternative regimen of atovaquone-proguanil (Malarone) (to which the parasite is sensitive) provided at the standard dosage of 1 gram/400 mg (4 adult tabs) orally once per day for three days. The infection will be treated as soon as parasites are identified by thick smear. It is anticipated that treatment will be curative; recrudescence has not occurred in any of the infected subjects. Following treatment, daily blood films will continue until three consecutive smears are negative. Subjects will be permitted to check out of the hotel after three consecutive daily smears are negative. Blood smears will be saved for later re-examination.

If malaria infection does not develop within the first 19 days post challenge, the subject will be released from staying nightly at the hotel. They may be required to visit to the Clinical Trials Center for evaluations and blood smears every other day Monday through Friday on study days 20 – 28 (excluding weekends and holidays). Assessment with phlebotomy will still be required if any symptoms develop, and if the subject is diagnosed with malaria, he or she will be treated according to the regimen described above. If a volunteer is still not parasitemic on day 28 post challenge, due to potential severity of the *P. falciparu*m infection, the volunteer will be treated presumptively in the same fashion as above.

The final in-person study visit for both immunized and control subjects will occur at 3 month after the challenge. All subjects will be instructed to contact the study team if they experience a fever any time within a year post challenge. If a fever or other signs or symptoms do occur within a year following the challenge, additional blood may be drawn for smears and laboratory tests. If subjects move outside of the Washington, D.C. area, they will be instructed to inform their medical care providers that they have been exposed to malaria and must have a blood smear checked whenever they develop a fever during the ensuing one year. Subjects will also be told to request that their medical care providers contact the study physicians for advice concerning the diagnosis and treatment of malaria infection. In the rare event that a subject would need to be hospitalized, this will be done at the Walter Reed Army Medical Center, Washington DC. Per FDA’s request, all the immunized subjects will be followed by phone, email, or US mail annually after the last in-person visit for a total of five years starting with the first immunization.

Subjects will not be able to donate blood for three years after the challenge per the American Red Cross guidelines. No study results will be sent to subjects at completion of the study.

- 1. **Concomitant Medications and Treatment**

At each study visit/contact, the investigator will question the subject about any medication taken. Any drugs or vaccine administered from 30 days prior to the immunization or challenge until 30 days after the challenge will be recorded in the CRF. The trade name and/or generic name of the medication, medical indication, total daily dose, route of administration, start and end dates of treatment will be included. Any other concomitant medication administered prophylactically in anticipation of reaction to the immunizations or challenge must also be recorded in the CRF with trade name and/or generic name of the medication, total daily dose, the route of administration, and start and end dates of treatment. It will be coded as ‘Prophylactic’. If the subject takes medications with antimalarial effects during the study, he or she may be withdrawn from further participation in the study. Subjects could withhold the use of these medications until after the study period.

- 1. **Sample and Data Handling**

All screening and follow-up diagnostic laboratory testing will be performed in an approved contract clinical laboratory (Quest). Urine pregnancy tests will be performed in the WRAIR Clinical Trial Center. Additional laboratory assays may be performed on stored serum samples remaining after protocol-specified laboratory testing is completed if volunteer has agreed and signed an informed consent form. This may include clinical tests performed during the evaluation of a subject’s medical problem or additional malaria-specific immunologic evaluations as they become available. Prior to storage, the blood may be processed into components at designated WRAIR laboratories. Blood and extracted elements will be stored at the malaria serology laboratory or at other designated locations at WRAIR.

- 1. **Holding Rules**

Further immunization will be put on hold by the Principal Investigator or by the medical monitor for the following adverse events:

- 1. Local adverse events: based on investigator discretion;
  2. Systemic adverse events: if two or more subjects during previous immunization develop a vaccine-related Grade 3 adverse event lasting > 48 hours beginning within 2 days after vaccination, if the adverse event is malaise, myalgia, fatigue, nausea, fever, headache, joint pain, or other systemic adverse event potentially related to the vaccine, or there is any vaccine-related Grade 4 adverse event;
  3. Laboratory adverse events: if two or more subjects during previous immunization develop a vaccine-related Grade 3 laboratory adverse event lasting > 72 hours beginning within 2 days after the vaccination, or there is any vaccine-related Grade 4 adverse event.

Once the study is put on hold, further decision requires a thorough review of all available data concerning the Adverse Event(s) by the Medical Monitor. Discussion will be led by the Medical Monitor with the investigators and the Safety Review Committee which will consist of the Medical Monitor and the Sponsor such as the USAMMDA safety officer who must be a physician among others. Immunization will restart only if all parties agree to resume. If the holding rule is activated, all IRBs will be notified in writing of such activation as well as the release from hold as decided by the above parties. Sponsor will notify the FDA in writing within 48 hours of the determination that the holding rule criterion has been met. In addition, the Sponsor will provide a written safety report to the FDA, per CFR section 312.32, for any “serious and unexpected” adverse event.

- 1. **Stopping Rules**

**A. For individual subjects:**

a**.** Local adverse events: based on investigator discretion;

- 1. Systemic adverse events: individual subjects will be withdrawn from further vaccination if they develop a vaccine-related Grade 3 adverse event lasting > 48 hours beginning within 2 days after vaccination if the adverse event is malaise, myalgia, fatigue, nausea, fever, headache, joint pain, or other systemic adverse event potentially related to the vaccine, or there is any vaccine-related Grade 4 adverse event.

**B. For the study:**

a. Local adverse events: based on investigator discretion;

b. Systemic adverse events: vaccination of a group will be stopped if 3 of 20 subjects during previous immunization develop a vaccine-related Grade 3 adverse event lasting > 48 hours beginning within 2 days after vaccination if the adverse event is malaise, myalgia, fatigue, nausea, fever, headache, joint pain, or there is any vaccine-related Grade 4 adverse event.

Once the study is stopped, decision regarding further course of this study requires a thorough review of all available data concerning the Adverse Event(s) by the Medical Monitor. Discussion will be led by the Medical Monitor with the investigators and the Safety Review Committee which will consist of the Medical Monitor and the Sponsor such as the USAMMDA safety officer who must be a physician among others. If the stopping rule is activated, all IRBs and FDA will be notified in writing of such activation as well as further decision made by the above parties.

1. **Additional Safety Considerations**

A medical monitor will be appointed for the study. The medical monitor is a qualified physician, not associated with the protocol, who will be responsible for serving as an advocate for the medical safety of the subjects, is able to provide care to research subjects for conditions that may arise during the conduct of the study and who is able to monitor subjects during the conduct of the study.

The medical monitor is also required to ensure that AEs are assessed correctly, review all SAEs, subject deaths associated with the protocol, and ensure proper management of unanticipated problems involving risk to subjects or others and provide an unbiased written report of the event to the WRAIR IRB. At a minimum, the medical monitor should comment on the outcomes of the event or problem and, in the case of a SAE or death, comment on the relationship to participation in the study. The medical monitor should also indicate whether he or she concurs with the details of the report provided by the study investigator.

1. **Adverse Events**

The investigator is responsible for the detection and documentation of events meeting the criteria and definition of an adverse event (AE) or serious adverse event (SAE) as provided in this protocol. During the study, when there is a safety evaluation, the investigator or site staff will be responsible for detecting AEs and SAEs, as detailed in this section of the protocol. Each subject will be instructed to contact the investigator immediately should they manifest any signs or symptoms they perceive as serious.

- 1. Solicited and Unsolicited Adverse Events
     1. Adverse Event Definition

An AE is any untoward medical occurrence in a study subject, temporally associated with the immunization, whether or not considered related to the immunization. An AE can therefore be any unfavorable and unintended sign (including an abnormal laboratory finding), symptom, or disease (new or exacerbated) temporally associated with any immunization, whether or not it is considered to be study related.

Examples of an AE include:

Exacerbation of a chronic or intermittent pre-existing condition includes either an increase in frequency and/or intensity of the condition.

- New conditions detected or diagnosed after any immunization is given even though it may have been present prior to the start of the study.
- Signs, symptoms, or the clinical sequelae of a suspected overdose of a concurrent medication (overdose per se should not be reported as an AE/SAE).
- Signs, symptoms temporally associated with the immunizations.

AEs may include post-treatment events that occur as a result of protocol-mandated procedures (i.e., invasive procedures, modification of subject’s previous therapeutic regimen). Pre-existing conditions or signs and/or symptoms present in a subject prior to the start of the study (i.e. prior to the first study procedure), as well as any that are not recognized at study entry but are recognized during the study period, should be recorded in the medical history section of the subject’s source document, and are not considered AEs. Signs and symptoms that occur after informed consent is obtained, but prior to the first dose of immunization will be documented in the Medical History form within the participant's source document. Anticipated day-to-day fluctuations of pre-existing conditions, including disease under study, that do not represent a clinically significant exacerbation need not be considered adverse events. Adverse events should be documented in terms of medical diagnosis when possible. When this is not possible, the adverse event should be documented in terms of signs and symptoms observed by the investigator or reported by the participant at each study visit.

- - 1. Surveillance Period for Occurrence of Adverse Events

All adverse events occurring within 28 days following administration of each vaccine must be recorded on the Adverse Event form in the participant's CRF, irrespective of severity or whether or not they are considered vaccination-related. The only exception is that since the challenge could occur within 28 days from the Ad immunization, the symptoms developed during the expected patency period after the challenge which are consistent with malaria infection will NOT be recorded as adverse events. See Section 8.2 for instructions for recording and reporting of serious adverse events.

- - 1. Recording and Reporting adverse events

At each visit/assessment, all adverse events either observed by the investigator, reported by the participant spontaneously, or in response to a direct question will be evaluated by the investigator. Adverse events not previously documented in the study will be recorded in the Adverse Event form within the participant's CRF. The nature of each event, date and time (where appropriate) of onset, outcome, intensity, and relationship to the immunization should be established.

If a adverse event changes in frequency or intensity during a 24-hour study period, the highest intensity will be recorded on the CRF. If the adverse event recurs with a defined interval of symptom-free time (>24 hours) in between episodes, a new record of the event will be started. Ongoing adverse events should be reviewed at subsequent visits.

Events that are not alarming and may reasonably be regarded as caused by or probably caused by the immunization should be batched together and reported to the DHSP/WRAIR IRB at the time of continuing review. See Section 8.2 for instructions for reporting and recording of unanticipated problems involving risk to others and/or serious adverse events.

Non-serious and serious adverse events will be evaluated as two distinct events given their different medical nature. If an event meets the criteria to be determined “serious” (see Section 8.2.1 for definition of serious adverse event), it will be examined by the investigator to the extent to be able to determine ALL contributing factors applicable to each serious adverse event.

Clinical laboratory parameters and other abnormal assessments qualifying as adverse events and serious adverse events that are judged by the investigator to be clinically significant will be recorded as AEs or SAEs if they meet the definition of an AE, as defined in Section 8.1.1 or SAE, as defined in Section 8.2.1. Clinically significant abnormal laboratory findings or other abnormal assessments that are detected during the study or are present at baseline and significantly worsen following the start of the study will be reported as AEs or SAEs. However, clinically significant abnormal laboratory findings or other abnormal assessments that are associated with the disease being studied (in our case, the immunization), unless judged by the investigator as more severe than expected for the subject’s condition, or that are present or detected at the start of the study and do not worsen, will not be reported as AEs or SAEs. The investigator will exercise his or her medical and scientific judgment in deciding whether an abnormal laboratory finding or other abnormal assessment is clinically significant

- - 1. Solicited Adverse Events

Solicited symptoms are adverse events occurring during the seven-day follow-up period after vaccination that are inquired about by the investigator or specified designee. Source documents and CRF will be allocated for the recording of solicited symptoms. Table 3a lists the solicited local and general adverse events for the study. In addition, we have elected to also monitor the cardiac adverse events post challenge, as listed in Table 3b, and will monitor these symptoms from day of challenge through 28 days post challenge.

Table 3a. Solicited local and general adverse events

|  | **Adverse events** |
| --- | --- |
| **Local (injection site)** | Pain/tenderness |
|  | Erythema |
|  | Induration/swelling |
| **General** | Fever* (Oral Temperature) |
|  | Malaise/Fatigue |
|  | Chills/Rigor |
|  | Headache |
|  | Myalgia |
|  | Arthralgia |
|  | Nausea |
|  | Vomiting |
|  | Diarrhea |
|  | Abdominal pain |
|  | Adenopathy |

N.B. Temperature will be recorded in the evening. Should temperature measurement additionally be performed at another time of day, the highest temperature will be recorded. * Fever is defined as oral temperature ≥ 38°C (100.4 °F).

^Adenopathy is to be monitored before and after the Ad vaccine only

Table 3b. Solicited cardiac adverse events post challenge

|  | **Adverse Events** |
| --- | --- |
| **Cardiac** | Chest Pain |
|  | Shortness of breath |
|  | Palpitation |
|  | Light-headedness |
|  | Syncope |

- - 1. Unsolicited Adverse Events

Unsolicited symptoms are adverse events reported by the subjects that are different from those solicited events. Space on the source documents and CRF will be allocated for the recording of unsolicited symptoms. Should any systemic (general) signs/symptoms be reported, their relationship with the immunization will be assessed by the investigator and transcribed into the CRF.

- - 1. Assessment of Intensity

Intensity of the solicited adverse events should be assessed as described in Tables 3 and 4. Intensity for all other AEs should be assessed using the scale in Table 6, and intensity grading scales for biochemical and hematological laboratory adverse events are in Tables 7 and 8. These values are originally chosen to match the reference laboratory (QUEST) normal values, although it should be noted that these normal values may be subject to change due to lab assay variation or changes in the normal ranges; in the latter case, the determination of the intensity will be based on the new normal values.

Table 4. Intensity grading of local solicited adverse events

| **Local Reaction to Immunization** | **Mild**  **(Grade 1)** | **Moderate**  **(Grade 2)** | **Severe**  **(Grade 3)** | **Potentially Life**  **Threatening**  **(Grade 4)** |
| --- | --- | --- | --- | --- |
| Pain/tenderness | Does not interfere  with activity | Repeated use of non-narcotic pain reliever > 24 hours or interferes with activity | Any use of narcotic pain reliever or prevents daily  activity | Emergency room  (ER) visit or  hospitalization |
| Erythema/  Redness * | < 5 cm | 5.1 – 10 cm | > 10 cm | Necrosis or  exfoliative  dermatitis |
| Induration/  Swelling | < 5 cm | 5.1 – 10 | > 10 cm | Necrosis |

* In addition to grading the measured local reaction at the greatest single diameter, the measurement should be recorded as a

continuous variable.

Table 5. Intensity grading of Fever and Systemic Solicited Adverse Events

| **Fever or Systemic**  **AE** | **Mild**  **(Grade 1)** | **Moderate**  **(Grade 2)** | **Severe**  **(Grade 3)** | **Potentially Life**  **Threatening**  **(Grade 4)** |
| --- | --- | --- | --- | --- |
| Fever (°C)**  (°F)* | 38.0 – 38.4  100.4 – 101.1 | 38.5 – 38.9  101.2 – 102.0 | 39.0 – 40  102.1 – 104 | > 40  > 104 |
| Malaise | No interference  with activity | Some interference  with activity | Significant; prevents  daily activity | ER visit or  hospitalization |
| Chills/  Rigors | No interference  with activity | Some interference  with activity | Significant; prevents  daily activity | ER visit or  hospitalization |
| Headache | No interference  with activity | Repeated use of non-narcotic  pain reliever > 24 hours or  some interference with  activity | Significant; any use of  narcotic pain reliever or  prevents daily activity | ER visit or  hospitalization |
| Myalgia | No interference  with activity | Repeated use of non-narcotic  pain reliever > 24 hr or  some interference with  activity | Significant; prevents  daily activity | ER visit or  hospitalization |
| Arthralgia | No interference  with activity | Repeated use of non-narcotic  pain reliever > 24 hr or  some interference with  activity | Significant; prevents  daily activity | ER visit or  hospitalization |
| Nausea/  vomiting | No interference  with activity or  1–2 episodes  over 24 hr | Some interference with  activity or > 2 episodes/24 hr | Prevents daily activity,  or requires outpatient IV  hydration | ER visit or  hospitalization for  hypotensive shock |
| Diarrhea | 2–3 loose stools  or < 400 gms  over 24 hr | 4–5 stools or  400–800 gms/24 hr | 6 or more watery stools  or > 800gms/24 hr or  requires outpatient IV  hydration | ER visit or  hospitalization |
| Abdominal  Pain | No interference  with activity | Repeated use of non-narcotic  pain reliever > 24 hr or  some interference with  activity | Significant; prevents  daily activity | ER visit or  hospitalization |
| Adenopathy^ | No interference  with activity | some interference with  activity | Significant; prevents  daily activity | ER visit or  hospitalization |

*Oral temperature; no recent hot or cold beverages or smoking.

^Adenopathy is to be monitored before and after the Ad vaccine only.

Table 6. Intensity grading of other adverse events

| Intensity |  | Description |
| --- | --- | --- |
| 0 |  | No adverse event |
| 1 |  | An adverse event that is easily tolerated by the subject, causing minimal discomfort and not interfering with everyday activities |
| 2 |  | An adverse event that is sufficiently discomforting to interfere with normal everyday activities |
| 3 |  | An adverse event that prevents normal, everyday activities i.e., prevents attendance at work and necessitates the administration of corrective therapy |

Table 7. Intensity grading for **Serum Biochemistry** Adverse Events

| **Adverse event** | **Intensity grade** | **Intensity** |
| --- | --- | --- |
| Creatinine (Males) | Normal range | > 0.5 and < 1.7 mg/dL |
|  | 1 | ≥ 1.7 and < 1.8 mg/dL |
|  | 2 | ≥ 1.8 and < 2.0 mg/dL |
|  | 3 | ≥ 2.0 mg/dL |
| Creatinine (Females) | Normal range | > 0.5 and < 1.4 mg/dL |
|  | 1 | ≥ 1.4 and < 1.6 mg/dL |
|  | 2 | ≥ 1.6 and < 1.8 mg/dL |
|  | 3 | ≥ 1.8 mg/dL |
| Liver Function Tests (AST/ALT) | |  |
| AST -– Males | Normal range | Age 20-49: 10-40, age > 50: 10-35 |
| AST -– Females | Normal range | Age 20-44: 10 – 30, age > 45: 10-35 |
| ALT -– Male | Normal range | 9-60 |
| ALT -– Female | Normal range | 6-40 |
|  | 1 | > 1.0 and < 2.5 times the upper limit of normal |
|  | 2 | ≥2.5 times and < 4 times the upper limit of normal |
|  | 3 | ≥4 times the upper limit of normal |

Table 8. **Hematology** Adverse Event intensity grading

| **Adverse event** | Normal range | **Grade 1** | **Grade 2** | **Grade 3** |
| --- | --- | --- | --- | --- |
| Low hemoglobin (Males), gm/dL | 13.2-17.1 | < 13.2 and ≥11.5 | < 11.5 and ≥10.5 | < 10.5 |
| Low hemoglobin (Females), gm/dL | 11.7-15.5 | < 11.7 and ≥11 | < 11 and ≥10L | < 10 |
| Decrease in hemoglobin (for both males and Females), gm/dL | <1.5 | 1.5-2.5 | > 2.5-3.5 | >3.5-4.5 |
| High leukocytes count (WBC) (x10^9^cells/L) | Per lab normal | ≥ 11 and < 15 | ≥ 15 and < 20 | ≥ 20 |
| Low leukocytes count(WBC) (x10^9^cells/L) | Per lab normal | < 3.5 and ≥2.5 | < 2.5 and ≥1.5 | < 1.5 |
| Low absolute neutrophil count (ANC, x10^9^cells/L) | >1.5 | 1.0 -1.49 | 0.50-0.99 | <0.50 |
| Low lymphocyte count (x10^9^cells/L) | > 1 | 0.75 – 1 | 0.5 – 0.749 | 0.25 – 0.499 |
| High eosinophil count (x10^9^cells/L) | Per lab normal | 0.65– 1.5 | 1.501 - 50 | > 50 |
| Decrease in Platelets (x10^9^cells/L) | > 125 | < 125 and ≥ 100 | < 100 and ≥ 75 | < 75 |

- - 1. Assessment of Causality

Every effort should be made by the investigator to explain each adverse event and assess its causal relationship to the immunization. The degree of certainty with which an adverse event can be attributed to immunization (or alternative causes, e.g., natural history of the underlying diseases, concomitant therapy, etc.) will be determined by how well the event can be understood in terms of 1) one or more of the reaction of similar nature having previously been observed with the same or similar vaccines and 2) the event being temporally associated with immunization.

All solicited local (immunization site) reactions will be considered causally related to immunization in the absence of clear evidence otherwise. In some instances, solicited systemic reactions during the ten-day hotel phase follow-up period may be determined to definitively not be causally related to the immunization (i.e., headache from trauma or fatigue due to excessive exercise). Such AEs will be listed in the solicited event page of the CRF with causality entered as “unrelated.”

Causality of all other adverse events should be assessed by the investigator using the descriptions in Table 9.

Table 9. Relationship between the adverse event and the study event for causality assessment

| Causality | Description |
| --- | --- |
| Not related | No relationship to study procedure. It applies to those events for which there is irrefutable evidence that there is another etiology, such as if the subject is injured as a passenger in a car accident. |
| Possible | An association between the AE and the immunization cannot be ruled out. Although there is a temporal association, there is another possibility that appears more likely. |
| Probable | There is a clear potential relationship to the investigational procedure, including a temporal association; however, the relationship is not definitive because another possibility exists. The other possibility is less likely to be the etiology than the immunization, but it cannot be ruled out with certainty. |
| Definite | An association exists between the immunization and the event. Any potential association to other factors can be ruled out. |

For serious or unexpected adverse events, efforts will be made to determine whether the AE is related to any or all of the study procedures which include the challenge and the treatment for malaria infection. The causality will be assessed based on the following categories and only when study procedures are involved, the degree of association (possible/probable /definite) will need to be assessed:

- - DNA vaccine
  - Ad5 vaccine
  - Malaria Challenge
  - Treatment
       1. Chloroquine
       2. Other, please specify _________
  - Undefined
  - Unrelated to study procedure
  - Possible
  - Probable
  - Definite.
  - Unrelated
    1. Adverse Event Follow-up

Investigators should follow-up non-serious adverse events until the subject completes the study, or until the AE stabilizes or resolves. Clinically significant laboratory abnormalities, as well as any adverse event, will be followed up until they have returned to normal, have a satisfactory explanation, or appropriate referral has been provided. Non-serious adverse events will be reported in compilation in annual study reports. Unanticipated and/or serious adverse events related to participation in the study will be promptly reported as described in Section 8.2.

- 1. **Serious and Unexpected Adverse Events**
     1. Definition of a Serious Adverse Event

A serious adverse event (SAE) is any untoward medical occurrence that results in any of the following outcomes:

1. results in death,

b. is a life-threatening adverse experience,

c. an important medical event,

d. hospitalization or prolongation of existing hospitalization,

e. results in a persistent or significant disability/incapacity, or

f. is a congenital anomaly/birth defect in the offspring of a study subject.

Although not considered as an adverse event, hospitalization for either elective surgery related to a pre-existing condition that did not increase in severity or frequency following initiation of the study, or for routine clinical procedures (including hospitalization for “social” reasons) that are not the result of an adverse event, must be recorded in the source documents. If the hospitalization arises from a pre-existing condition, or was planned prior to the initiation of the study, the condition that led to hospitalization should be recorded in the Medical History form of the source documents. If the hospitalization arises from a pre-existing condition, and the hospitalization was planned after the initiation of the study, the condition that led to hospitalization should be recorded in the adverse event page of the CRF. In both cases, the condition that led to hospitalization should be recorded, and the relationship to the immunization will be recorded as “Not related”.

Medical or scientific judgment should be exercised in deciding whether reporting is appropriate in other situations, such as important medical events that may not be immediately life-threatening or result in death or hospitalization, but may jeopardize the subject or may require medical or surgical intervention to prevent one of the other outcomes listed in the above definition. These should also be considered serious.

Definition of an unexpected adverse event is any adverse test procedure experience, the specificity or severity of which is not consistent with the general investigational plan.

- - 1. Surveillance Period for Occurrence of Serious Adverse Events

All serious adverse events occurring within the study period will be recorded and reported during the active stage of follow-up. During the long-term stage of follow-up over four years, all SAEs will be recorded but only related SAEs will be reported.

- - 1. Reporting a Serious or Unexpected Adverse Event

Serious Adverse Events will be reported in a prospective manner during the period starting from the day of the first immunization to each subject and ending with the last study visit. Unanticipated problems involving risk to subjects or others and serious adverse events deemed to be related or possibly related to participation in the study and all subject deaths should be promptly reported by the investigator to WRAIR IRB through the Director, Division of Human Subjects Protection, Walter Reed Army Institute of Research within 48 hours (two calendar days) of becoming aware of the event. The contact information is listed below. WRAIR IRB will in turn inform USAMRMC ORP HRPO and NMRC IRB. Efforts will be made to notify the Sponsor within 48 hours as well; however, the Sponsor does allow up to 72 hours for notification. The Sponsor will in turn report to FDA according to applicable timeline.

The investigator will document all available information regarding the serious adverse event in a written report or the SAE CRF. The investigator should not wait to receive additional information to fully document the event before notifying the Sponsor or DHSP/WRAIR IRB of a serious adverse event. This initial notification should give at minimum, sufficient information to permit identification of:

- The reporter
- The subject identification number, date of birth, gender and ethnicity
- Study procedure
- Date of vaccine administration
- Adverse events (date of onset, signs/symptoms and severity)
- Action taken
- Concomitant medications (dose, route, duration)

As new information is obtained, updated reports should be submitted, and if available, should include copies of relevant hospital case records, autopsy reports, and other documents where applicable.

In the event that a serious adverse event is determined to be related to the immunization, no further immunization will be administered until a written report has been submitted to the DHSP/WRAIR IRB, and the investigators have conferred with the Sponsor and Medical Monitor.

During the 4-year long-term follow-up stage, if an instance of congenital abnormality in offspring is brought to the attention of the investigator at any time after cessation of immunization AND suspected to be related to immunization, it should be reported to the Study Contacts for Serious Adverse Event Reporting.

Investigators should follow-up subjects with serious adverse events until the event has resolved, subsided, stabilized, disappeared, is otherwise explained, or the subject/patient is lost to follow-up. Outcome should be assessed as:

1 = Recovered

2 = Recovered with sequelae

3 = Ongoing at subject study conclusion (active phase)

4 = Died

5 = Unknown

The medical monitor is required to review all unanticipated problems involving risk to subjects or others and serious adverse events and provide an unbiased written report of the event.  At a minimum, the medical monitor should comment on the outcomes of SAE or the unexpected event and comment on the relationship to participation in the study.  The medical monitor should also indicate whether he/she concurs with the details of the report provided by the study investigator.

All information should be sent promptly to the contacts listed below:

| DHSP/WRAIR IRB:  Division of Human Subjects Protection (DHSP)  Walter Reed Army Institute of Research  503 Robert Grant Ave  Silver Spring, MD 20910-7500  Tel: 301-319-9940  Fax: 301-319-9961  Electronic mail (preferred method): [wrairdhsp@amedd.army.mil](mailto:wrairdhsp@amedd.army.mil) |
| --- |
| Sponsor’s Representative Contact Information for Reporting Serious Adverse Events  USAMRMC  Division of Regulated Activities and Compliance  ATTN: MCMR-UMR 1430 Veterans Drive Ft. Detrick, MD 21702-9232 Tel: 301-619-0317 Fax: 301-619-0197  Email: USAMRMCREGULATORYAFFAIRS @amedd.army.mil |
| Human Subject Research Review Board (HSRRB)  Human Research Protection Office (HRPO)  Office of Research Protections (ORP)  United States Army Medical Research and Materiel Command (USAMRMC)  Fort Detrick, Maryland  Phone: 301-619-6197  Fax: 301-619-7803  Email: [hsrrb@amedd.army.mil](mailto:hsrrb@amedd.army.mil) |
| NMRC IRB  Naval Medical Research Center Institutional Review Board  Chairman of IRB, CDR David Fryauff,  500 Robert Grant Ave  Silver Spring, MD 20910-7500  Tel: 301-319-7276  Fax: 301-319-7277  david.fryauff@med.navy.mil |
| Independent Medical Monitor  Arthur Lyons, MD, PhD, MC, USA  Chief, Clinical Research Unit  Division of Viral Diseases  Walter Reed Army Institute of Research  503 Robert Grant Ave.  Silver Spring, MD 20910  301-319-9021 |

- 1. **Pregnancy**

Subjects will be instructed to notify the investigator if they became pregnant either during the study or within 30 days of the study completion of the active stage of study. Subjects who become pregnant during the study period may be asked to complete other study procedures to include blood draws for immunogenicity and safety and review of symptoms/physical exams, at the discretion of the Principal Investigator. The Medical Monitor will be notified of the pregnancy and can recommend if additional safety evaluations are needed. Pregnancy is not considered an adverse event, it will be reported in the same way as study deviation. IRBs will be notified once the study team learns of the pregnancy, the follow-up report will be submitted in the next continuing report. A pregnancy will be followed to term by the Principal Investigator. Any premature terminations will be reported, and the health status of the mother and child including date of delivery and the child’s gender and weight will be reported to DHSP/WRAIR IRB after delivery.

- 1. **Treatment of Adverse Events**

The principal investigator will ensure each subject receives appropriate treatment of any AE related to immunization or the challenge.

1. **Data Evaluation**
   1. **Data Inclusion Criteria**

Analysis will be primarily per protocol; intention to treat will be analysis secondarily. Immunogenicity will be assessed only in those subjects who were immunized, and protective efficacy will be assessed only in those subjects who were immunized and challenged. Drop-outs prior to immunization will not be included in those analyses. Data collected from subjects enrolled in the study will be analyzed unless the subject missed three or more out the five immediate follow-up visits associated with an immunization. If a subject withdrew from the study, only data collected to the point of withdrawal will be included. In the case of spurious data, if the integrity of the data cannot be positively ascertained, the study design team will decide whether to keep the data or not. Non-analyzable data will be documented in the deviations. A case report form (CRF) has been produced for this study, and a database designed based on the CRF will be generated at the WRAIR CTC.

- - 1. Cohort for analysis of safety

The analysis of safety/reactogenicity will include all enrolled subjects and will include subjects for whom data are available for the analysis of safety/reactogenicity. Thus, the total analysis of safety will include all subjects with at least one vaccine administration documented, and who have sufficient data to perform an analysis of safety.

- - 1. Cohort for analysis of immunogenicity

The analysis of immunogenicity will include all subjects for whom differential treatment effect on immunogenicity is likely (i.e., those meeting all eligibility criteria, complying with the procedures defined in the protocol) and will include subjects for whom assay results are available for antibodies or for cellular immunity against the study vaccine antigen component after at least one immunization.

- 1. **Analysis of Demographics**

Demographic characteristics (age, sex, and race) of each study cohort will be tabulated. The mean age (plus range and standard deviation) by sex of the enrolled subjects, as a whole and per group, will be calculated.

- 1. **Analysis of Primary Endpoints**
     1. Analysis of Safety and Tolerability

The vaccine will be considered safe and well-tolerated if there are no severe or serious AEs related to vaccine administration or if any severe events are relatively benign (e.g. erythema meeting criteria for severe due to its dimensions but not significantly affecting the activities of daily living for the subject) and brief in duration (e.g. less than 48 hours). The overall percentage of subjects with at least one local AE (solicited or unsolicited) or with at least one general AE (solicited or unsolicited) during the seven-day follow-up period after immunization, will be tabulated. In addition, the incidence, intensity, and relationship of individual solicited symptoms over the seven-day follow-up period will be calculated for the entire study group as well as for the Ad5+ and Ad5-subgroups. The number of subjects with at least one report of an unsolicited adverse event reported up to 14 days after immunization will also be tabulated for the entire study group and for the subgroups. The intensity and temporal relationship of the unsolicited symptoms to immunization will also be assessed.

- - 1. Analysis of Efficacy

Vaccine efficacy will be assessed two to four weeks after the challenge and will be based on the number of subjects in the immunized group and the number of subjects in the control group with malaria. Protective efficacy will be calculated as shown below:

| Relative Risk = | (# positive in immunized group)/(# challenged in immunized group) |
| --- | --- |
|  | (# positive in control group)/(# challenged in control group) |

Efficacy = 1- Relative Risk, 95% confidence interval for relative risk will be calculated.

If immunized subjects are not completely protected against malaria infection, meaning that the disease onset is delayed or the disease in general is milder than the unimmunized persons, immunized and control subjects will be ranked according to time of onset of parasitemia and log rank test will be performed to look for delays in parasitemia induced by immunization.

For discrete variables (e.g. number of responders, the number of positive assays, the number of individuals protected against challenge), the chi-squared test or Fisher’s exact test will be used (two-tailed, uncorrected for chi-squared), except when the cell value is five or less, in which case only Fisher’s exact test will be used (two-tailed). For days to parasitemia, subjects will be rank-ordered and a non-parametric test suitable for unpaired groups (e.g., Mann Whitney) will be employed (one-tailed when comparing a vaccine group to controls). In addition, days to parasitemia will be assessed using a Cox Proportional Hazards model and will be displayed using Kaplan-Meier plots.

- 1. **Analysis of Secondary Endpoints**
     1. Analysis of Immunogenicity

Immunogenicity will be assessed by the number of responders, the magnitude of response, and the number of positive assays. Measurements with putative normal distributions expressed as means of continuous data (e.g., magnitude of immune responses) will be assessed using the Student’s t test (two-tailed), paired if pre-immunization values are compared with post immunization values, and unpaired if comparisons are made between groups. For discrete variables with normal distributions (e.g. number of responders, the number of positive assays), the chi-squared test or Fisher’s exact test will be used (two-tailed, uncorrected for chi-squared), unless the cell value is five or less, in which case only Fisher’s exact test will be used (two-tailed). Immunological comparisons between vaccine groups and infectivity controls will be one-tailed.

Each PBMC or serum/plasma sample will be assayed by ELISpot or ELISA assays in triplicate or triplicate. For each triplicate or triplicate, outliers will be rejected if any single triplicate (or triplicate) value contributes more than 50% of the standard deviation of the triplicate (or triplicate) and if its value is three-fold greater or less than the average of the remaining two (or three) values. After removing outliers, the mean spot forming cells (SFCs, ELISpot), optical density (OD) (ELISA, at each serum dilution), or fluorescent signal (flow) obtained in negative control wells/tubes (PBS or malaria-naïve sera) will be subtracted from the value of each well/tube. Negative counts, if any are generated by this background subtraction, will be converted to zero. The mean and standard deviation of the test sample will then be calculated. Antibody levels will be log-transformed before analysis. Previous experience with ELISpot, ELISA, and flow-based assays suggests that the total counts will be normally distributed; if they are not, then non-parametric methods will be used for the comparisons.

For growth inhibition assays (GIA) growth inhibition will be calculated from final parasitemia as:

Inhibition = (control-test)/control, where control is the final parasitemia with pre-immune serum. Selected positive sera and controls will be retested and titered out under four conditions:

- 3D7 static;

- 3D7 suspension

- FVO static

- FVO suspension.

(Using the heterologous FVO parasite in GIA will give an indication of possible cross-protection.)

GIA results will be reported in a tabular manner as the percent growth inhibition seen at a given serum dilution compared with a negative control serum, such as a pre-immune serum from the same subject.

Immunological outcomes expressed as means (e.g., magnitude of responses, titer of antibodies as determined by endpoint dilution, OD values, concentration of cytokines as determined by reference to positive standard controls) will be compared between groups using the Student’s t-test (two-tailed), paired if pre-immunization values are compared with post-immunization values, unpaired if comparisons are made between groups. Those outcomes expressed as proportions (prevalence of antibodies, frequency of cytokine responses, frequency of positive assays) will be compared between groups by chi-^2^ test. In all analyses, p<0.05 will be considered statistically significant. True difference in response proportions relative to negative controls will be also calculated (95% CI) for flow cytometry data: if the confidence interval is entirely above 0.05%, the response is positive; if the confidence interval is entirely below 0.05%, the response is negative; if the confidence interval overlaps with 0.05%, the response is indeterminate. Assay sensitivity, specificity, and positive and negative predictive values (+ 95% CI) (with respect to protective immunity) will be established using standard formulae.

Humoral Responses

For IFAT, seroconversion will be defined as a four-fold rise, as compared to the pre-immune serum, against intact *P. falciparum* sporozoites or blood stage parasites by IFAT. For ELISA, seroconversion will be defined as a mean optical density of ELISA assays (conducted in triplicates) against recombinant protein or synthetic peptide antigens at a specified (e.g., 1:100 or 1:200) dilution of serum that is greater than the mean plus two standard deviations of the mean of triplicate pre-immunization serum at the same dilution. A serum will be considered to be GIA positive if, compared with a negative control serum, the growth inhibition value is 10% or more and with a two-tailed students’s t test p value of less than 0.05. Response to immunizations will be calculated relative to each individual subject’s pre-immune results for each assay.

Cellular Responses

A positive response for ELISpot will have the following three characteristics: (1) a statistically significant difference between the number of spot forming cells in triplicate test wells and triplicate control wells; (2) at least a doubling of spot forming cells in test wells relative to control wells; and (3) a difference of at least five spots between test and control wells. Samples will be considered positive by intracellular cytokine staining assays if they have the following three characteristics: (1) the response to test antigens is greater than 0.01%; (2) the response to test antigens is greater than two-fold compared to the background responses to the control protein and PBS-treated samples; and (3) there is a true difference in response proportions (95% confidence interval). For both assays, responses to immunizations will be calculated relative to pre-immune specimens.

Immunogenicity will be assessed by the number of responders, the magnitude of response, and the number of positive assays.

- - 1. Subgroup Analysis

A subgroup analysis based on the Ad5 serology status will be conducted to assess if there were significant differences for safety/tolerability, immunogenicity and efficacy between the Ad5+ and Ad5- groups. If the sample size is too small for p-value or confidence interval calculation, comments will be made based on the general trend.

- 1. **Final Report**

A final report will be created encompassing the safety and immunogenicity data collected during the entire trial period. The results will be determined using cleaned data, reported by group and will not identify individuals. There will be no interim report.

- 1. **Future Plans**

If this vaccine regimen involving DNA-Ad vaccines demonstrates significant protection (>30%), evaluation in a multi-component vaccine regimen will be initiated. Progression to a Phase 2b study to assess protection among endemic population will be initiated.

1. **Administrative Matters**

To comply with Good Clinical Practice important administrative obligations relating to investigator responsibilities, monitoring, archiving data, audits, confidentiality and publications must be fulfilled. See Attachment B for details.

- 1. **Publication Policy**

It is anticipated that the results of this study will be presented to the scientific community via oral presentations and written publications. Any proposed publication or presentation will be first submitted and agreed to by any involved author(s) prior to publication/presentation. It will also be reviewed and approved by the relevant authorities at NMRC/WRAIR prior to public submission.

1. **References**

1. Breman, J.G., M.S. Alilio, and A. Mills, *Conquering the intolerable burden of malaria: what's new, what's needed: a summary.* Am J Trop Med Hyg, 2004. **71**(2 Suppl): p. 1-15.

2. Susi, B., et al., *Rapid diagnostic test for Plasmodium falciparum in 32 Marines medically evacuated from Liberia with a febrile illness.* Ann Intern Med, 2005. **142**(6): p. 476-7.

3. Meis, J.F., et al., *Plasmodium falciparum: studies on mature exoerythrocytic forms in the liver of the chimpanzee, Pan troglodytes.* Exp Parasitol, 1990. **70**(1): p. 1-11.

4. Yoshida, N., et al., *Hybridoma produces protective antibodies directed against the sporozoite stage of malaria parasite.* Science, 1980. **207**(4426): p. 71-3.

5. Herrington, D., et al., *Successful immunization of humans with irradiated malaria sporozoites: humoral and cellular responses of the protected individuals.* Am J Trop Med Hyg, 1991. **45**(5): p. 539-47.

6. Malik, A., et al., *Human cytotoxic T lymphocytes against the Plasmodium falciparum circumsporozoite protein.* Proc Natl Acad Sci U S A, 1991. **88**(8): p. 3300-4.

7. Zevering, Y., et al., *Major population differences in T cell response to a malaria sporozoite vaccine candidate.* Int Immunol, 1990. **2**(10): p. 945-55.

8. Doolan, D.L., et al., *Cytotoxic T lymphocyte (CTL) low-responsiveness to the Plasmodium falciparum circumsporozoite protein in naturally-exposed endemic populations: analysis of human CTL response to most known variants.* Int Immunol, 1993. **5**(1): p. 37-46.

9. Doolan, D.L. and S.L. Hoffman, *Pre-erythrocytic-stage immune effector mechanisms in Plasmodium spp. infections.* Philos Trans R Soc Lond B Biol Sci, 1997. **352**(1359): p. 1361-7.

10. Doolan, D.L., Hoffman, S.L., *The complexity of protective immunity against liver-stage malaria.* J. Immunol., 2000. **165**(3): p. 1453-62.

11. Stoute, J.A., et al., *A preliminary evaluation of a recombinant circumsporozoite protein vaccine against Plasmodium falciparum malaria. RTS,S Malaria Vaccine Evaluation Group.* N Engl J Med, 1997. **336**(2): p. 86-91.

12. Stoute, J.A., et al., *Long-term efficacy and immune responses following immunization with the RTS,S malaria vaccine.* J Infect Dis, 1998. **178**(4): p. 1139-44.

13. Kester, K.E., et al., *Efficacy of recombinant circumsporozoite protein vaccine regimens against experimental Plasmodium falciparum malaria.* J Infect Dis, 2001. **183**(4): p. 640-7.

14. Doherty, J.F., et al., *A phase I safety and immunogenicity trial with the candidate malaria vaccine RTS,S/SBAS2 in semi-immune adults in The Gambia.* Am J Trop Med Hyg, 1999. **61**(6): p. 865-8.

15. Bojang, K.A., et al., *Efficacy of RTS,S/AS02 malaria vaccine against Plasmodium falciparum infection in semi-immune adult men in The Gambia: a randomised trial.* Lancet, 2001. **358**(9297): p. 1927-34.

16. Alonso, P.L., et al., *Efficacy of the RTS,S/AS02A vaccine against Plasmodium falciparum infection and disease in young African children: randomised controlled trial.* Lancet, 2004. **364**(9443): p. 1411-20.

17. Alonso, P.L., et al., *Duration of protection with RTS,S/AS02A malaria vaccine in prevention of Plasmodium falciparum disease in Mozambican children: single-blind extended follow-up of a randomised controlled trial.* Lancet, 2005. **366**(9502): p. 2012-8.

18. Aponte, J.J., et al., *Safety of the RTS,S/AS02D candidate malaria vaccine in infants living in a highly endemic area of Mozambique: a double blind randomised controlled phase I/IIb trial.* Lancet, 2007. **370**(9598): p. 1543-51.

19. Sacarlal, J., et al., *Safety of the RTS,S/AS02A malaria vaccine in Mozambican children during a Phase IIb trial.* Vaccine, 2008. **26**(2): p. 174-184.

20. Silvie, O., et al., *A role for apical membrane antigen 1 during invasion of hepatocytes by Plasmodium falciparum sporozoites.* J Biol Chem, 2004. **279**(10): p. 9490-6.

21. Narum, D.L. and A.W. Thomas, *Differential localization of full-length and processed forms of PF83/AMA-1 an apical membrane antigen of Plasmodium falciparum merozoites.* Mol Biochem Parasitol, 1994. **67**(1): p. 59-68.

22. Healer, J., et al., *Independent translocation of two micronemal proteins in developing Plasmodium falciparum merozoites.* Infect Immun, 2002. **70**(10): p. 5751-8.

23. Duraisingh, M.T., T. Triglia, and A.F. Cowman, *Negative selection of Plasmodium falciparum reveals targeted gene deletion by double crossover recombination.* Int J Parasitol, 2002. **32**(1): p. 81-9.

24. Malkin, E.M., et al., *Phase 1 clinical trial of apical membrane antigen 1: an asexual blood-stage vaccine for Plasmodium falciparum malaria.* Infect Immun, 2005. **73**(6): p. 3677-85.

25. Polhemus, M.E., et al., *Phase I dose escalation safety and immunogenicity trial of Plasmodium falciparum apical membrane protein (AMA-1) FMP2.1, adjuvanted with AS02A, in malaria-naive adults at the Walter Reed Army Institute of Research.* Vaccine, 2007. **25**(21): p. 4203-12.

26. Thera, M.A., et al., *Safety and immunogenicity of an AMA-1 malaria vaccine in Malian adults: results of a phase 1 randomized controlled trial.* PLoS ONE, 2008. **3**(1): p. e1465.

27. Doolan, D.L. and S.L. Hoffman, *DNA-based vaccines against malaria: status and promise of the Multi-Stage Malaria DNA Vaccine Operation.* Int J Parasitol, 2001. **31**(8): p. 753-62.

28. Catanzaro, A.T., et al., *Phase I clinical evaluation of a six-plasmid multiclade HIV-1 DNA candidate vaccine.* Vaccine, 2007. **25**(20): p. 4085-92.

29. Wang, R., et al., *Induction of CD4(+) T cell-dependent CD8(+) type 1 responses in humans by a malaria DNA vaccine.* Proc Natl Acad Sci U S A, 2001. **98**(19): p. 10817-22.

30. Epstein, J.E., et al., *Safety, tolerability, and lack of antibody responses after administration of a PfCSP DNA malaria vaccine via needle or needle-free jet injection, and comparison of intramuscular and combination intramuscular/intradermal routes.* Hum Gene Ther, 2002. **13**(13): p. 1551-60.

31. Wang, R., et al., *Boosting of DNA vaccine-elicited gamma interferon responses in humans by exposure to malaria parasites.* Infect Immun, 2005. **73**(5): p. 2863-72.

32. Tavel, J.A., et al., *Safety and immunogenicity of a Gag-Pol candidate HIV-1 DNA vaccine administered by a needle-free device in HIV-1-seronegative subjects.* J Acquir Immune Defic Syndr, 2007. **44**(5): p. 601-5.

33. Graham, B.S., et al., *Phase 1 safety and immunogenicity evaluation of a multiclade HIV-1 DNA candidate vaccine.* J Infect Dis, 2006. **194**(12): p. 1650-60.

34. Miao, J., et al., *Immune responses in mice induced by prime-boost schemes of the Plasmodium falciparum apical membrane antigen 1 (PfAMA1)-based DNA, protein and recombinant modified vaccinia Ankara vaccines.* Vaccine, 2006. **24**(37-39): p. 6187-98.

35. Wang, R., et al., *Induction of antigen-specific cytotoxic T lymphocytes in humans by a malaria DNA vaccine.* Science, 1998. **282**(5388): p. 476-80.

36. Dunachie, S.J., et al., *A DNA prime-modified vaccinia virus ankara boost vaccine encoding thrombospondin-related adhesion protein but not circumsporozoite protein partially protects healthy malaria-naive adults against Plasmodium falciparum sporozoite challenge.* Infect Immun, 2006. **74**(10): p. 5933-42.

37. Chinchilla, M., et al., *Enhanced immunity to Plasmodium falciparum circumsporozoite protein using Salmonella Typhi expressing PfCSP and a PfCSP-encoding DNA vaccine in a heterologous prime-boost strategy.* Infect Immun, 2007.

38. Dunachie, S.J., et al., *A clinical trial of prime-boost immunisation with the candidate malaria vaccines RTS,S/AS02A and MVA-CS.* Vaccine, 2006. **24**(15): p. 2850-9.

39. Rodrigues, E.G., et al., *Interferon-gamma-independent CD8+ T cell-mediated protective anti-malaria immunity elicited by recombinant adenovirus.* Parasite Immunol, 2000. **22**(3): p. 157-60.

40. Rodrigues, E.G., et al., *Single immunizing dose of recombinant adenovirus efficiently induces CD8+ T cell-mediated protective immunity against malaria.* J Immunol, 1997. **158**(3): p. 1268-74.

41. Gilbert, S.C., et al., *Enhanced CD8 T cell immunogenicity and protective efficacy in a mouse malaria model using a recombinant adenoviral vaccine in heterologous prime-boost immunisation regimes.* Vaccine, 2002. **20**(7-8): p. 1039-45.

42. Bruna-Romero, O., et al., *Complete, long-lasting protection against malaria of mice primed and boosted with two distinct viral vectors expressing the same plasmodial antigen.* Proc Natl Acad Sci U S A, 2001. **98**(20): p. 11491-6.

43. Bruna-Romero, O., et al., *Enhanced protective immunity against malaria by vaccination with a recombinant adenovirus encoding the circumsporozoite protein of Plasmodium lacking the GPI-anchoring motif.* Vaccine, 2004. **22**(27-28): p. 3575-84.

44. Stewart, V.A., et al., *Priming with an adenovirus 35-circumsporozoite protein (CS) vaccine followed by RTS,S/AS01B boosting significantly improves immunogenicity to Plasmodium falciparum CS compared to that with either malaria vaccine alone.* Infect Immun, 2007. **75**(5): p. 2283-90.

45. Reyes-Sandoval, A., J.T. Harty, and S.M. Todryk, *Viral vector vaccines make memory T cells against malaria.* Immunology, 2007. **121**(2): p. 158-65.

46. Rodrigues, E.G., et al., *Efficient induction of protective anti-malaria immunity by recombinant adenovirus.* Vaccine, 1998. **16**(19): p. 1812-7.

47. Sallusto, F., et al., *Two subsets of memory T lymphocytes with distinct homing potentials and effector functions.* Nature, 1999. **401**(6754): p. 708-12.

48. Masopust, D., et al., *Preferential localization of effector memory cells in nonlymphoid tissue.* Science, 2001. **291**(5512): p. 2413-7.

49. Tough, D.F., *Deciphering the relationship between central and effector memory CD8+ T cells.* Trends Immunol, 2003. **24**(8): p. 404-7.

50. Wherry, E.J., et al., *Lineage relationship and protective immunity of memory CD8 T cell subsets.* Nat Immunol, 2003. **4**(3): p. 225-34.

51. Le, T.P., et al., *Safety, tolerability and humoral immune responses after intramuscular administration of a malaria DNA vaccine to healthy adult volunteers.* Vaccine, 2000. **18**(18): p. 1893-901.

52. van Belle, G., *Statistical Rules of Thumb*. 1st ed. 2002, New York: Wiley-Interscience. 49-50.

53. Gaziano, T.A., et al., *Laboratory-based versus non-laboratory-based method for assessment of cardiovascular disease risk: the NHANES I Follow-up Study cohort.* Lancet, 2008. **371**(9616): p. 923-31.
